# Dysregulation of astrocytic DNAJC6 contributes to sporadic Parkinson’s disease pathogenesis

**DOI:** 10.1172/JCI194989

**Published:** 2026-04-09

**Authors:** Wahyu Handoko Wibowo Darsono, Yeongran Hwang, Erica Valencia, Leonardo Tejo Gunawan, Seung Jae Hyeon, Hoon Ryu, Thor D. Stein, Mi-Yoon Chang, Noviana Wulansari, Sang-Hun Lee

**Affiliations:** 1 Department of Biochemistry and Molecular Biology, College of Medicine,; 2 Hanyang Biomedical Research Institute, and; 3 Graduate School of Biomedical Science and Engineering, Hanyang University, Seoul, South Korea.; 4 Brain Science Institute, Korea Institute of Science and Technology, Seoul, South Korea.; 5 Boston University Alzheimer’s Disease Center and Department of Neurology, Boston University Chobanian & Avedisian School of Medicine, Boston, Massachusetts, USA.; 6 Department of Premedicine, College of Medicine, and; 7 Hanyang Institute of Bioscience and Biotechnology, Hanyang University, Seoul, South Korea.

**Keywords:** Clinical Research, Neuroscience, Parkinson disease

## Abstract

Loss-of-function mutations in DNAJC6, encoding the cochaperone auxilin (HSP40 family), cause familial juvenile-onset Parkinson’s disease (PD). Given the chaperone role of DNAJC6 in cellular homeostasis in adult neurons, we hypothesized that DNAJC6 dysfunction may not be limited to juvenile-onset disorders but could also be associated with adult-onset brain diseases. Here, we show that DNAJC6 expression is significantly downregulated in postmortem substantia nigra tissues and transcriptomic datasets from patients with late-onset sporadic PD. Consistently, human pluripotent stem cell–derived midbrain cultures exhibited reduced DNAJC6 expression under multiple PD-associated conditions. Mechanistically, DNAJC6 loss resulted from impaired transcription mediated by the midbrain-specific factors NURR1/FOXA2 and reduced protein stability regulated by LRRK2. Beyond neurons, DNAJC6 was robustly expressed in astrocytes and similarly downregulated in sporadic PD contexts. Astrocytic DNAJC6 deficiency impaired phagocytic, autolysosomal, and mitochondrial functions while promoting a proinflammatory phenotype, thereby exacerbating neurodegenerative pathology. Importantly, epigenetic restoration of DNAJC6 in neurons and astrocytes using a CRISPRa-AAV9 system in the substantia nigra of an α-synuclein–induced PD mouse model alleviated behavioral deficits and neuropathology. These findings provide evidence that DNAJC6 dysregulation is associated with pathogenic processes in sporadic PD and suggest that targeting neuronal and astrocytic DNAJC6 could represent a potential disease-modifying strategy.

## Abstract 2

pathogenic role of DNAJC6 in sporadic PD and highlight the therapeutic potential of targeting neuronal and astrocytic DNAJC6 dysregulation in PD

## Introduction

Parkinson’s disease (PD), the most common neurodegenerative movement disorder, is characterized by progressive loss of dopamine (DA) neurons in the substantia nigra (SN) of the midbrain. Intraneuronal α-synuclein (α-syn) aggregates, mitochondrial dysfunction, and oxidative stress contribute to midbrain DA (mDA) neuron degeneration. Analyses of PD-associated genes (PARK genes) further revealed impairments in vesicular trafficking and endolysosomal processes leading to accumulation of toxic proteins and damaged organelles without proper degradation (reviewed in ref. 1). In addition to cell-autonomous mechanisms, mDA neuron degeneration is triggered and amplified by neuroinflammation and pathological glia, including astrocytes and microglia. Recent genome-wide and RNA-seq studies highlighted gliocentric pathological changes in neurodegenerative disorders, reinforcing the substantial contribution of glia to disease pathogenesis (2–6).

DNAJC6 (DNAJ/HSP40 homolog, subfamily C member 6; MIM 608375), which encodes auxilin, is a PD gene (PARK19) associated with loss-of-function mutations in familial juvenile parkinsonism (hereafter, DNAJC6 refers to both the gene and the protein [auxilin]) (7–9). We recently identified a pathogenic mechanism involving neurodevelopmental defects in mDA neuron precursor cells carrying PD-specific DNAJC6 loss-of-function mutations (10), providing insight into early disease onset. Beyond development, DNAJC6 regulates key functions in postmitotic neurons, including synaptic recycling, endocytosis, vesicular trafficking, and autolysosomal clearance (7, 10–12). These findings suggest that DNAJC6 dysfunction may contribute not only to juvenile-onset PD but also to adult-onset brain disorders. Given its roles in mitochondrial, endosomal, and autolysosomal homeostasis across multiple cell types, DNAJC6 is likely important for nonneuronal glial physiology as well. Consequently, glial DNAJC6 dysfunction may contribute to adult-onset brain disease pathogenesis.

In this study, we show that DNAJC6 is downregulated in brains of patients with late-onset sporadic PD and in multiple PD-related contexts, and we investigate mechanisms underlying this reduction. Although DNAJC6 has often been described as predominantly neuronal, we detected substantial expression in astrocytes. Astrocytic DNAJC6 was also downregulated in sporadic PD, contributing to disease pathogenesis. Finally, we demonstrate that restoring DNAJC6 expression may represent a therapeutic strategy in an α-syn–induced PD mouse model.

## Results

### Downregulation of DNAJC6 in the brains of patients with sporadic PD.

**Figure 1:**
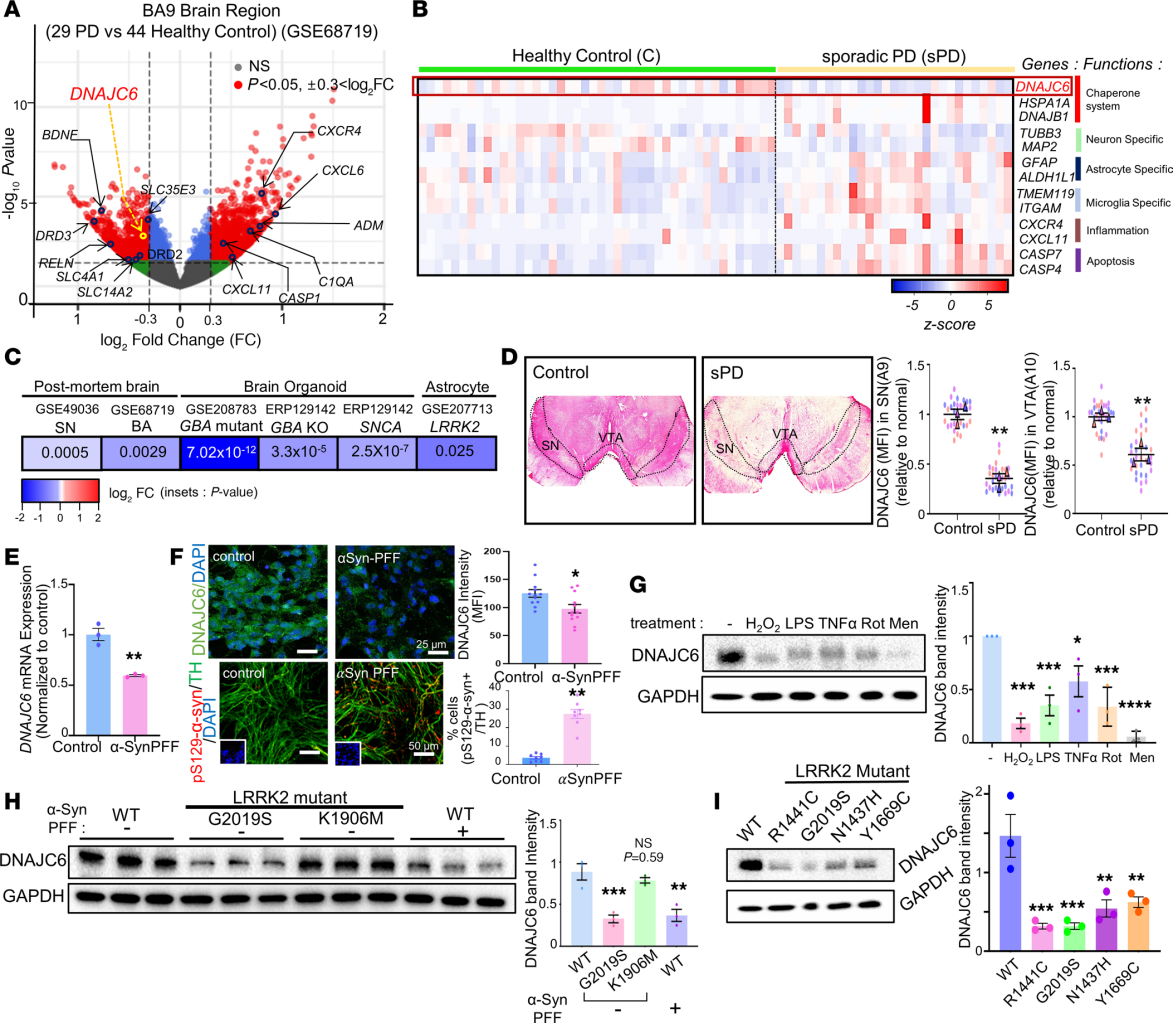
Downregulation of DNAJC6 expression in sporadic PD contexts. (**A**) Scatterplot showing up- and downregulated genes in the transcriptomes of postmortem brains (Brodmann area 9 [BA9]) from 29 patients with sporadic PD versus 44 acting as controls (GSE68719). (**B**) Heatmap illustrating gene expression profiles of interest, highlighting the downregulation of DNAJC6 in PD patients’ brains compared with controls (GSE68719). (**C**) DNAJC6 expression levels across multiple transcriptomic datasets from sporadic PD brains and PD patient–derived cells harboring PD-associated gene variants (GBA, SNCA, and LRRK2). Data are presented as log_2_ FC of DNAJC6 expression in PD versus controls, with statistical significance indicated by *P* values. (**D**) Protein levels of DNAJC6 in the midbrain SN and ventral tegmental area (VTA) of patients with sporadic PD (sPD) compared with age-matched controls. Original magnification, ×1. A total of 30 cells were counted (10 cells/case; control, *N* = 3; PD patient, *N* = 3); **P < 0.01; nested 2-tailed *t* test. (**E**–**G**) Downregulation of DNAJC6 under various in vitro conditions mimicking sporadic PD contexts. Human midbrain neuron/astrocyte cultures (derived from hESCs, H9) were exposed to PD-associated toxins, including α-syn PFFs (2 μg/mL), H_2_O_2_ (200 μM), LPS (1 μg/mL), TNF-α (20 ng/mL), rotenone (100 nM), and menadione (20 μM) for 48 h before analysis. α-Syn pathology was assessed by percent pS129-α-syn^+^/TH^+^ cells (**F**, lower panel). α-Syn PFF treatment was applied under in vitro conditions mimicking sporadic PD (**E** and **F**; detailed analysis is shown in Supplemental Figure 12). Scale bars in **F**: 25 μm (top), 50 μm (bottom). (**H** and **I**) Analysis of human midbrain cultures derived from PD-iPSCs carrying LRRK2 variants (and WT iPSCs). DNAJC6 expression was assessed via qPCR (**E**), MFI in ICC (**F**), and WB (**G**–**I**) analyses. *n* = 3~4 independent experiments (**E** and **G**–**I**), *n* = 12 (**F**, % DNAJC6 intensity) or 8 (**F**, % p-S129 α-syn) independent cultures; *P < 0.05, **P < 0.01, ***P < 0.001, ****P < 0.0001; unpaired 2-tailed *t* test (**E** and **F**) or 1-way ANOVA followed by Dunnett’s test (**G**–**I**).

The established roles of DNAJC6 in postmitotic adult neurons (7, 10–12) led us to hypothesize that its dysfunction may also contribute to late-onset sporadic PD and PD with other genetic backgrounds. To test this, we analyzed transcriptome datasets from patients with sporadic PD and age-matched controls. In postmortem brain samples (Brodmann area 9; GSE68719) from 29 patients with PD and 44 patients acting as controls, 1,184 genes were upregulated (log_2_ fold change [FC] > 0.3, *P* > 0.05) and 1,110 were downregulated (Figure 1A). Upregulated genes were enriched in Gene Ontology categories related to neuroinflammation and glial activation, whereas downregulated genes were associated with neuronal and synaptic functions, including DA secretion, synaptic transmission, and vesicle exocytosis (Supplemental Figure 1A; supplemental material available online with this article; https://doi.org/10.1172/JCI194989DS1). Notably, DNAJC6 was among the significantly downregulated genes (log_2_ FC = −0.35, *P* = 0.0029) (Figure 1, A–C). Reduced DNAJC6 mRNA expression was also observed in SN samples from patients with advanced-stage sporadic PD (Braak stages 5–8; GSE49036) (Figure 1C). IHC analysis on postmortem human brain samples validated the decrease of DNAJC6 protein levels in the midbrain SNs of patients with sporadic PD compared with age-matched subjects treated as control (Figure 1D). Significant DNAJC6 downregulation was further detected in brain organoids derived from sporadic PD patient induced pluripotent stem cells (iPSCs) carrying GBA N370S (GSE208783), GBA deletion (ERP129142), and α-syn (SNCA) overexpression (ERP129142), as well as in astrocytes derived from LRRK2 G2019S PD patients (GSE207713) (Figure 1C). Across these datasets, DNAJC6 reduction was accompanied by enrichment of Gene Ontology and KEGG pathways related to apoptosis, cellular senescence, inflammatory response, and NF-κB signaling, along with consistent downregulation of pathways involved in dopaminergic synapse, synapse assembly, axon guidance, nervous system development, and calcium signaling (Supplemental Figure 1B).

GAK (auxilin-2), reported to be compensatorily upregulated in DNAJC6-KO mice (11), was modestly increased in PD brains (GSE68719; log_2_ FC = 0.21, *P* = 0.005) (Supplemental Figure 2A). In contrast, synaptojanin 1 (SYNJ1; PARK20), which also regulates clathrin-mediated endocytosis (CME), was downregulated. GAK upregulation accompanied by SYNJ1 downregulation was also observed in 2 additional datasets analyzed in Figure 1C (GSE208783 and ERP129142) (Supplemental Figure 2A). Several HSP40 cochaperones (DNAJC10, DNAJC18, DNAJC22, and DNAJB7) were significantly reduced in PD brains, whereas DNAJB1 was increased. HSP70 family members (HSPA6, HSPA1A, HSPA1B, and HSPA1L) and small heat shock proteins (HSPB1, HSPB2, and HSPB9) showed a trend toward upregulation (Supplemental Figure 2B). Although chaperone gene expression changes were also observed in other PD datasets, the specific genes affected varied (Supplemental Figure 2B).

To investigate mechanisms underlying DNAJC6 downregulation, we generated human midbrain cultures containing mDA neurons and astrocytes from human embryonic stem cells (hESCs) (H9) (Supplemental Figure 3A) (13) and exposed them to PD-related stressors. Treatment with α-syn preformed fibrils (PFFs) induced robust α-syn pathology, marked by pS129-α-syn^+^ puncta (Figure 1F). DNAJC6 mRNA and protein levels were markedly reduced following α-syn PFF treatment (Figure 1, E and F). DNAJC6 protein levels also declined after exposure to oxidative (H_2_O_2_), inflammatory (LPS and TNF-α), and mitochondrial (rotenone and menadione) stressors (Figure 1G). These reductions were accompanied by varying degrees of neuronal and synaptic loss (Supplemental Figure 4, A and B), suggesting that DNAJC6 downregulation represents a convergent response to diverse PD-associated insults and may contribute to disease progression. Finally, DNAJC6 protein levels were significantly lower in midbrain cultures derived from human iPSCs (hiPSCs) carrying pathogenic LRRK2 variants (G2019S, R1441C, N1437H, and Y1669C) compared with isogenic WT or kinase-dead LRRK2 K1906M controls (Figure 1, H and I). Collectively, these findings support the downregulation of DNAJC6 as a feature of late-onset sporadic PD.

### Mechanisms for DNAJC6 downregulation in the PD context.

**Figure 2:**
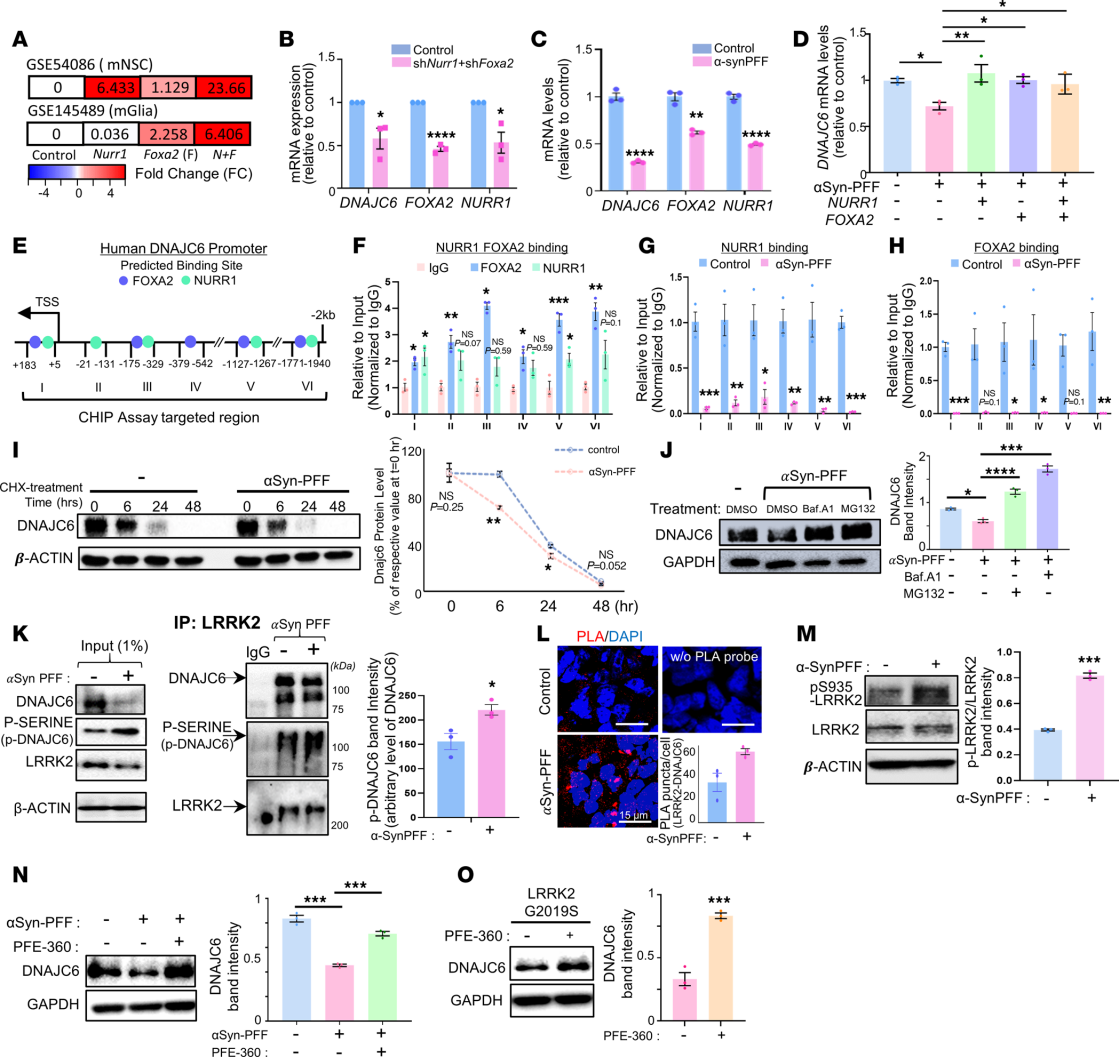
Mechanisms underlying the loss of DNAJC6 in PD environments: impaired Nurr1- and Foxa2-mediated transcription and LRRK2-induced protein instability. (**A**) DNAJC6 expression levels in mouse NSC (GSE54086) and glia cultures (GSE145489) transduced with Nurr1 (N) and Foxa2 (F) compared with mock controls (C). Data are presented as fold changes of N + F/C (indicated within bars) with corresponding bar color intensity. (**B**) Downregulation of DNAJC6 mRNA levels by shRNA-mediated KD of Nurr1 and Foxa2 in mouse primary midbrain neuron + astrocyte cultures, as measured by qPCR. (**C**) qPCR-based determination of DNAJC6, Nurr1, and Foxa2 mRNA expression levels in hESC-derived midbrain cultures treated with α-syn PFF. (**D**) Rescue of DNAJC6 downregulation in α-syn PFF-treated human midbrain cultures by forced expression of Nurr1 and Foxa2, as measured by qPCR. (**E**) Schematic representation of predicted Nurr1 and Foxa2 binding sites on the human DNAJC6 promoter. TSS, transcription start site. (**F**) ChIP-qPCR analysis showing Nurr1 and Foxa2 protein binding at DNAJC6 promoter regions identified in **E**. (**G** and **H**) ChIP-qPCR analysis demonstrating reduced Nurr1 and Foxa2 recruitment to the DNAJC6 promoter in human midbrain cultures treated with α-syn PFF. (**I**) DNAJC6 protein stability assay in α-syn PFF-treated and untreated human midbrain cultures. Representative WB images and quantification of DNAJC6 levels normalized to β-actin over 48 h following cycloheximide (CHX) treatment (50 μg/mL). (**J**) Effects of autophagy (Bafilomycin A1) and proteasome (MG132) inhibitors on DNAJC6 protein stability in α-syn PFF-treated human midbrain cultures. (**K** and **L**) Physical interaction between DNAJC6 and LRRK2 proteins demonstrated by co-IP (**K**) and proximity ligation assay (PLA; **L**) in α-syn PFF-treated and untreated human midbrain cultures. (**K**) Co-IP analysis showing LRRK2-mediated serine phosphorylation of DNAJC6. Proteins from human midbrain cultures, either treated or untreated with α-syn PFF, were immunoprecipitated using an anti-LRRK2 antibody, followed by immunoblotting with an anti-DNAJC6 antibody. Left blot: 1% input of whole-cell lysate. Right blot: immunoblot analysis of co-IP proteins probed for DNAJC6 and Pan-phosphoserine (P-SERINE). The DNAJC6 and phosphorylated DNAJC6 bands are indicated by arrows. Normal IgG was used as a negative control to confirm binding specificity. The LRRK2 IP blot demonstrates the successful enrichment of the protein. Scale bars: 15 μm. (**M**) Increased LRRK2 kinase activity in response to α-syn PFF treatment, estimated by LRRK2 autophosphorylation at Ser935. (**N** and **O**) Rescue of DNAJC6 protein decline by LRRK2 inhibition (PFE-360) in α-syn PFF-treated human midbrain cultures (**N**) and in midbrain cultures derived from LRRK2 G2019S-hiPSCs (**O**). *n* = 3 independent reactions (**B**–**H**), *n* = 3 independent experiments (**I**–**O**); *P < 0.05, **P < 0.01, ***P < 0.0001, ****P < 0.0001; unpaired 2-tailed *t* test (**B**, **C**, **F**–**H**), 1-way ANOVA followed by Tukey’s post hoc analysis (**D**, **J**, and **N**), or paired 2-tailed *t* test (**I**, **K**–**M**, and **O**).

The midbrain transcription factors NURR1 (NR4A2) and FOXA2 are essential for mDA neuron and glial function (14–16), but their expression is highly susceptible to loss in PD contexts (16–19). In our microarray data (GSE54086 and GSE145489) examining NURR1- and FOXA2-induced gene expression changes in primary mouse neural stem cell (mNSC) and glial cultures, DNAJC6 was among the top genes upregulated by forced expression of Nurr1 and Foxa2 (Figure 2A and Supplemental Figure 5). Knockdown (KD) of NR4A2 and FOXA2 in primary mouse midbrain cultures resulted in a significant reduction of DNAJC6 mRNA levels (Figure 2B). This effect was observed without a significant change in apoptotic cell death (control: 1.58% ± 0.2% vs. shNurr1 + shFoxa2: 1.50% ± 0.13%; *P* = 0.76, 2-tailed *t* test), ruling out neuronal loss as the primary cause of DNAJC6 downregulation and supporting a cell-autonomous regulatory mechanism. In midbrain cultures derived from human pluripotent stem cells (hPSCs), α-syn PFF treatment reduced NR4A2 and FOXA2 expression along with DNAJC6 mRNA and protein levels (Figure 2C and Supplemental Figure 6, A–C). Forced expression of Nurr1 and Foxa2 reversed these effects (Figure 2D and Supplemental Figure 6C). ChIP revealed enrichment of Nurr1 and Foxa2 at multiple regions of the human DNAJC6 promoter (Figure 2, E and F), which was significantly diminished after α-syn PFF treatment (Figure 2, G and H). These findings indicate that impaired Nurr1/Foxa2-mediated transcription drives DNAJC6 downregulation in the α-syn PD context. Consistent with previous reports showing mitochondrial and oxidative stress–induced suppression of Nurr1 and Foxa2 (16, 20, 21), we observed similar reductions in midbrain cultures exposed to mitochondrial, oxidative, and inflammatory toxins — conditions that also decreased DNAJC6 expression (Supplemental Figure 4C). Thus, disruption of the Nurr1/Foxa2 transcriptional program may represent a common mechanism underlying DNAJC6 suppression in PD.

Beyond transcriptional regulation, DNAJC6 protein stability was reduced in α-syn–treated cultures, as shown by cycloheximide assays (Figure 2I). The α-syn–mediated decrease in DNAJC6 protein was blocked by Bafilomycin A1 (autophagy inhibitor) or MG132 (proteasome inhibitor) (Figure 2J), indicating involvement of both degradation pathways. Since protein stability is often regulated by posttranslational modifications, and LRRK2 has been reported to phosphorylate DNAJC6 at Ser627 (22), we examined their interaction. Co-IP and proximity ligation assays confirmed a physical interaction between LRRK2 and DNAJC6 (Figure 2, K and L). As reported (23), α-syn PFF treatment increased LRRK2 kinase activity, reflected by elevated autophosphorylation at Ser935 (Figure 2M) and enhanced serine phosphorylation of DNAJC6 (Figure 2K). This phosphorylation was absent in midbrain cultures derived from hiPSCs carrying the kinase-dead LRRK2 K1906M variant (Supplemental Figure 7). Treatment with the LRRK2 inhibitor PFE-360 restored DNAJC6 protein levels in α-syn–treated cultures (Figure 2N) and in LRRK2 G2019S mutant hiPSC-derived cultures (Figure 2O). Together, these results suggest that DNAJC6 downregulation in PD arises from both impaired Nurr1/Foxa2-dependent transcription and reduced protein stability driven by LRRK2 hyperactivity.

### DNAJC6 expression in astrocytes and its downregulation in a PD context.

**Figure 3:**
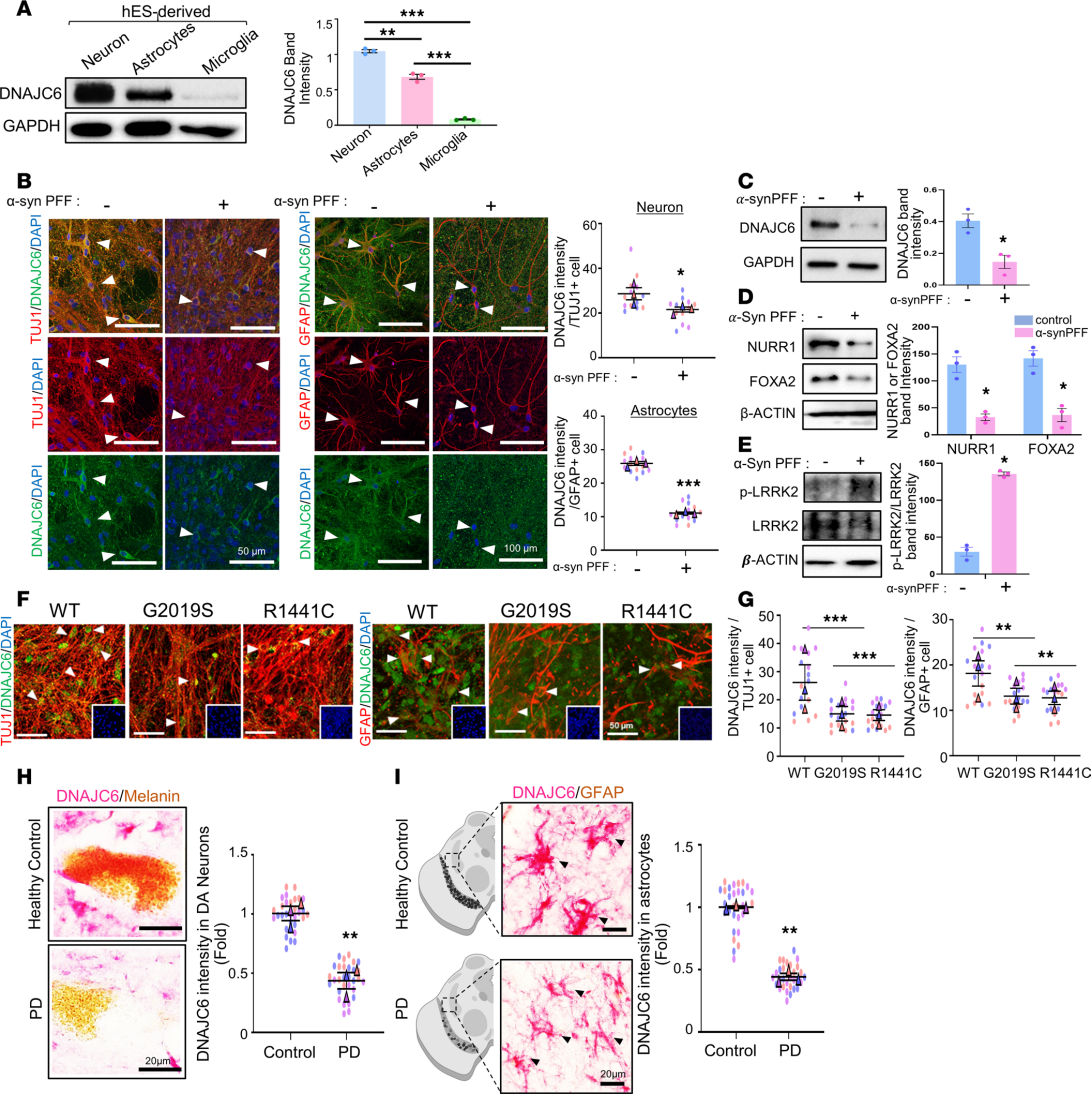
Decline of DNAJC6 levels in astrocytes within the context of PD. (**A**) WB analysis showing substantial DNAJC6 protein expression in neurons and astrocytes but not in microglia, all derived from hESCs. *n* = 3 independent replicates; **P < 0.01, ***P < 0.001; 1-way ANOVA. (**B**) Decreased DNAJC6 levels in neurons and astrocytes within mixed neuron–astrocyte human midbrain cultures treated with α-syn PFF. Arrowheads indicate representative neurons (TUBB3^+^) and astrocytes (GFAP^+^) costained with DNAJC6. The DNAJC6 expression levels in these cell types were estimated by measuring the MFI using LAS image analysis (Leica). A total of 14 neurons or 15 astrocytes were counted (4–5 cells/culture; *N* = 3 independent cultures); *P < 0.05, ***P < 0.001; nested 2-tailed *t* test. Scale bars: 50 μm (left), 100 μm (right). (**C** and **D**) Downregulation of DNAJC6 (**C**), and Nurr1 and Foxa2 (**D**) in human midbrain astrocyte cultures (derived from hESCs) following α-syn PFF treatment, as assessed by WB. *n* = 3 independent experiments; *P < 0.05; unpaired 2-tailed *t* test. (**E**) α-Syn PFF treatment effect on astrocytic LRRK2 activity. LRRK2 kinase activity was estimated by LRRK2 autophosphorylation at Ser935. *n* = 3 independent experiments; *P < 0.05; paired 2-tailed *t* test. (**F** and **G**) Reduced DNAJC6 expression in midbrain-type neurons and astrocytes carrying LRRK2 mutations. *n* = 18 cells were counted (6 cells/culture; *N* = 3 independent cultures); **P < 0.01, ***P < 0.001; nested 1-way ANOVA. Scale bars: 50 μm. (**H** and **I**) Reduced DNAJC6 immunoreactivity in DA neurons (melanin^+^ neurons) (**H**) and astrocytes (**I**) of the SN in sporadic PD patients (*N* = 3) compared with control (*N* = 3). A total of 30 cells were counted (10 cells/case); **P < 0.01; nested 2-tailed *t* test. Scale bars: 20 μm.

DNAJC6 has been identified as a neuron-specific gene involved in synaptic transmission, autolysosomal clearance, and cell survival (10, 11). However, substantial DNAJC6 expression was detected in midbrain-type astrocytes derived from hESCs (Figure 3, A–D, and Supplemental Figure 8), whereas expression was minimal in hESC-derived microglia (Figure 3A and Supplemental Figure 8). These cell type–specific patterns were supported by data from The Human Protein Atlas (proteinatlas.org). In mixed human neuron–astrocyte cultures treated with α-syn PFF, DNAJC6 expression was reduced in both βIII-tubulin^+^ (TUBB3^+^) neurons and GFAP^+^ astrocytes (Figure 3B). The reduction of astrocytic DNAJC6 was further confirmed in purified midbrain-type astrocyte cultures (Figure 3C).

Importantly, NURR1, FOXA2, and LRRK2 are also expressed in midbrain astrocytes (15, 24), where they have been implicated in critical pathological processes relevant to PD (15, 16, 24–26). In our astrocyte cultures, α-syn PFF treatment decreased NURR1 and FOXA2 expression while increasing LRRK2 activity (Figure 3, D and E), suggesting that α-syn–induced DNAJC6 downregulation in astrocytes is mediated by reduced NURR1/FOXA2 expression and enhanced LRRK2 activity, as shown in Figure 2.

DNAJC6 levels were also significantly lower in neurons and astrocytes derived from hiPSCs carrying PD-associated LRRK2 G2019S and R1441C variants compared with WT controls (Figure 3, F and G). Consistently, transcriptome analysis (GSE207713) showed significant DNAJC6 downregulation in LRRK2 G2019S hiPSC-derived astrocytes (Figure 1C and Supplemental Figure 9A). This reduction was accompanied by enrichment of genes associated with DNAJC6-related pathways, including endocytosis, lysosomal transport, and WNT signaling (Supplemental Figure 9B), a pathway regulated by DNAJC6 (10).

Finally, DNAJC6 expression was detected in melanin^+^ human mDA neurons and astrocytes in postmortem SN samples, with clear downregulation in PD brains (Figure 3, H and I). Together, these results show that DNAJC6 levels decline in astrocytes in PD, prompting further investigation into the pathophysiological role of astrocytic DNAJC6 in disease progression.

### Role of DNAJC6 in astrocytic endocytosis, lysosomal protein trafficking, and function.

**Figure 4:**
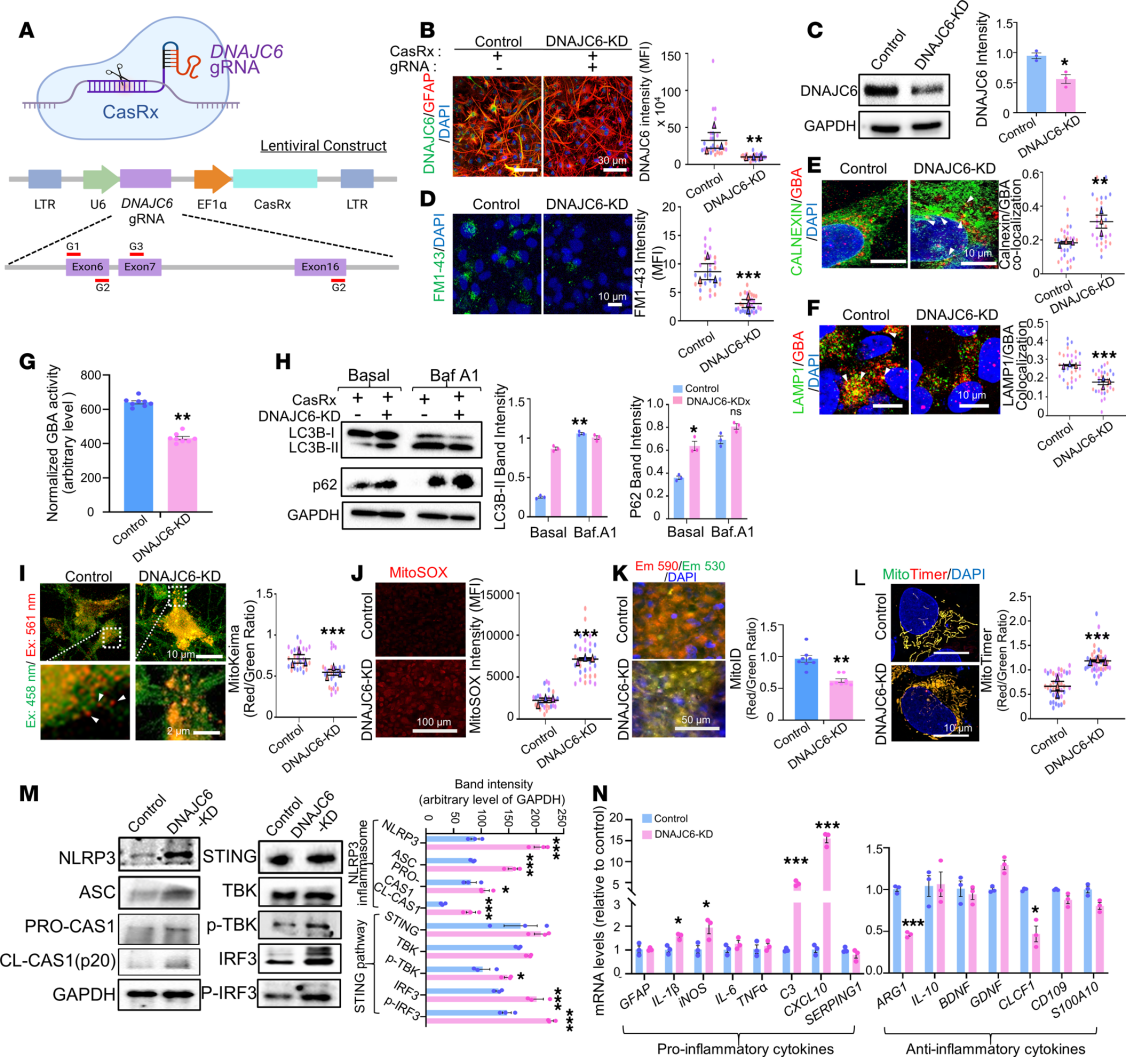
DNAJC6 KD in astrocytes promotes a proinflammatory phenotype through disrupted vesicular trafficking, endolysosomal dysfunction, and mitochondrial damage. (**A**) Schematic of CRISPR/CasRx system with the vector constructs used for DNAJC6 KD. (**B** and **C**) Validation of CasRx-mediated DNAJC6 KD efficiency in hESC-derived midbrain astrocyte cultures assessed by ICC (**B**) and WB (**C**) analysis. Scale bars: 30 μm. (**D**) Clathrin-mediated endocytic capacity determined by the uptake of FM1-43 dye. Scale bar: 10 μm. (**E** and **F**) Trafficking of glucocerebrosidase A (GBA) proteins from the ER/Golgi to lysosomes, evaluated by colocalization of GBA^+^ puncta with the ER marker Calnexin (**E**) and the lysosomal marker LAMP1 (**F**). Arrowheads point to the GBA^+^/Calnexin^+^ (**E**) or LAMP1^+^/GBA^+^ (**F**) puncta. Scale bars: 10 μm. (**G**) GBA enzyme activities in lysosomal fractions. (**H**) Autolysosomal clearance analyzed by WB. (**I** and **J**) Mitophagy analyzed by MitoKeima (**I**; arrowheads indicate red mt-Keima in lysosomes) and MitoSox (**J**). Scale bars: 10 μm (**I**, top), 2 μm (**I**, bottom), 100 μm (**J**). (**K**) Mitochondrial membrane potential assessed by MitoID. Scale bar: 50 μm. (**L**) MitoTimer (mitochondrial biogenesis) assays. Scale bars: 100 μm. (**M**) WB analysis of key components involved in the cGAS/STING and NLRP3 inflammasome pathways. (**N**) qPCR analysis of proinflammatory and antiinflammatory/neurotrophic cytokines. All experiments were conducted in hESC-derived astrocyte cultures with DNAJC6 KD (CasRx + gRNAs) or control cultures without KD (CasRx alone). Ten cells were counted for each experiment, *N* = 3 independent experiments (**B**, **D**–**F**, and **I**–**L**), *n* = 8 (**G**) or 3 (**C**, **H**, **M**, and **N**) independent experiments; *P < 0.05, **P < 0.01, ***P < 0.001; nested 2-tailed *t* test (**B**, **D**–**F**, and **I**–**L**), unpaired 2-tailed *t* test (**N**), or paired 2-tailed *t* test (**C**, **G**, **H**, and **M**).

To examine the role of DNAJC6 in astrocyte homeostasis, we knocked down DNAJC6 in midbrain-type astrocytes derived from H9 hESCs using the CRISPR-Rx system (Figure 4A). This RNA-targeting approach provides higher efficiency and fewer off-target effects than conventional siRNA or shRNA methods. Effective KD by the CasRx-DNAJC6 lentiviral system was confirmed in astrocyte cultures (Figure 4, B and C).

Consistent with DNAJC6’s role in CME (27), FM1-43 dye uptake assays showed reduced endocytic capacity in CasRx-mediated DNAJC6-KD astrocytes compared with mock-transduced controls (Figure 4D). As lysosomal protein trafficking also depends on clathrin-mediated pathways, we assessed glucocerebrosidase 1 (GBA1, GCase), a lysosomal enzyme linked to α-synucleinopathy and PD. In DNAJC6-KD astrocytes, GBA1 was significantly enriched in the ER (Calnexin^+^) (Figure 4E), while GBA1^+^ puncta in lysosomes (LAMP1^+^) were reduced (Figure 4F). This was accompanied by decreased GBA enzymatic activity in the lysosomal fraction (Figure 4G), indicating impaired ER/Golgi-to-lysosome trafficking.

In the basal condition, we found an increase in the autophagosome components LC3II and p62 protein levels in the DNAJC6-KD astrocytes compared with the mock-transduced control astrocytes (Figure 4H), indicating increased initial autophagosome formation or decreased autolysosomal degradation at the late autophagy stage. The LC3II and p62 protein levels increased in the presence of Bafilomycin A1, an inhibitor of autolysosomal degradation at the late stage of autophagy, in the control cultures, but not significantly (or slightly increased) in the DNAJC6-KD astrocyte cultures (Figure 4H), indicating a blockade at the late stage of autolysosomal degradation. Together, these findings demonstrate that astrocytic DNAJC6 supports endocytosis and lysosomal enzyme trafficking, thereby contributing to proper autolysosomal clearance.

### Knocking down DNAJC6 in astrocytes caused mitochondrial damage that led to proinflammatory phenotype changes.

Given the roles of astrocytic DNAJC6 in lysosomal trafficking and autophagy, we examined its involvement in mitophagy, a key mitochondrial quality control process. The MitoKeima assay showed impaired progression of mitophagy to the late lysosomal degradation stage in DNAJC6-KD astrocytes, reflected by a reduced red/green puncta ratio (Figure 4I). In contrast, levels of PINK1 and Parkin in mitochondrial fractions, which initiate mitophagy, were unchanged (Supplemental Figure 10). Together with the lysosomal defects observed in Figure 4, E–H, these findings suggest that DNAJC6 primarily regulates downstream lysosomal degradation stages of mitophagy.

Consistent with defective mitophagy, DNAJC6-KD astrocytes exhibited increased mitochondrial ROS (MitoSox) (Figure 4J) and decreased mitochondrial membrane potential (MitoID) (Figure 4K). Using MitoTimer — a DsRed mutant fused to a mitochondrial targeting sequence that shifts from green (new/healthy) to red (oxidized/damaged) fluorescence (28) — we observed higher red/green ratios in KD astrocytes, confirming mitochondrial damage (Figure 4L).

Mitochondrial stress in glia can activate inflammatory pathways (29–31), including the NLRP3 inflammasome and STING pathway by detecting mtDNAs in the cytosol released from the damaged mitochondria (32, 33). In DNAJC6-KD astrocyte cultures, NLRP3 inflammasome activation was evidenced by increased levels of its components (NLRP3, ASC, and procaspase-1) and its product, cleaved caspase-1 (Figure 4M). Notably, the downstream signaling molecules of the STING pathway — TBK1 and IRF3 — were activated in KD astrocytes (Figure 4N), yet this activation occurred without changes in the upstream molecules cGAS and STING, which are typically triggered by cytosolic mtDNA detection. MAVS from damaged mitochondria can directly activate the TBK1/IRF3 inflammatory pathways through its interaction with TBK1 (34, 35). In the DNAJC6-KD astrocytes, MAVS activation was manifested by its increased aggregation (Supplemental Figure 11, B and C), while no increase of mtDNA in the cytosol was detected (Supplemental Figure 11A), indicating the activation of MAVS, but not mtDNA-activated cGAS-STING, is responsible for the activated TBK1/IRF3 inflammatory pathway.

Consistent with this inflammatory activation, DNAJC6-KD astrocytes displayed a proinflammatory transcriptional profile, including upregulation of IL1B, NOS2, IL6, CXCL10, and C3, and downregulation of antiinflammatory genes ARG1, CLCF1, and S100A10 (Figure 4N).

### DNAJC6 KD in astrocytes aggravates α-syn–induced neurodegeneration in vitro.

To investigate the impact of astrocyte-specific DNAJC6 KD on PD-related pathologies, we used an in vitro human PD model (36, 37). In this system, mDA neurons overexpressing α-syn were cocultured with hESC-derived astrocytes and microglia and exposed to α-syn PFFs (Supplemental Figure 12A). The model recapitulated key features of α-synucleinopathy and mDA neurodegeneration, including insoluble pathological α-syn aggregates detected by Western blot (WB) of Triton X-100–insoluble fractions (Supplemental Figure 12B) and immunocytochemistry (ICC) for Thioflavin S^+^ and pS129-α-syn^+^ aggregates (Supplemental Figure 12, C and D). As neuronal α-synucleinopathy involves clustering of α-syn oligomers in synaptic vesicles (38–40), α-syn PFF treatment induced clumping of Synapsin-1^+^ (SYN1^+^) vesicles along tyrosine hydroxylase^+^ (TH^+^) neuronal fibers (Supplemental Figure 12E). The PD model did not alter astrocyte numbers (Supplemental Figure 12F).

**Figure 5:**
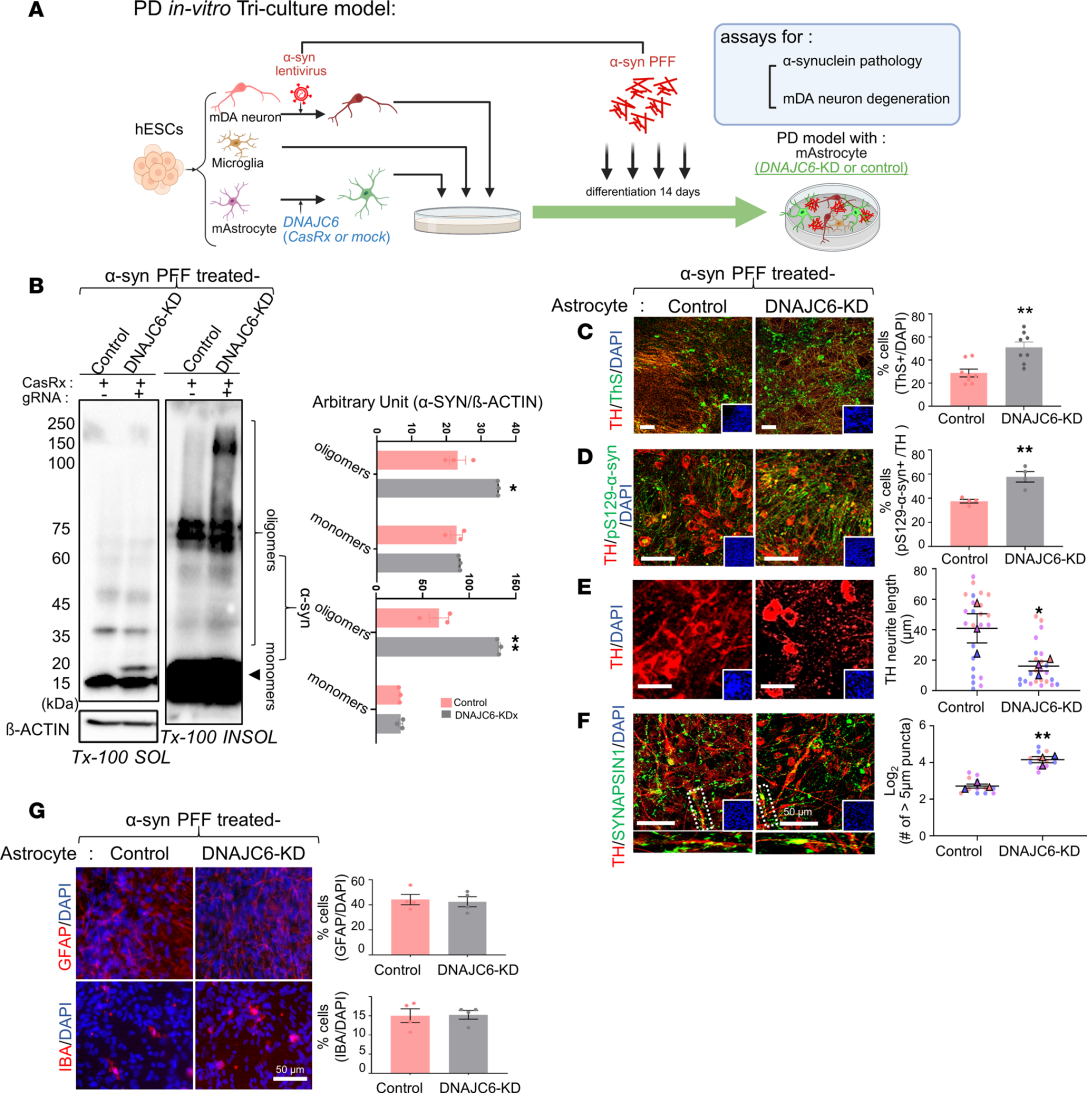
DNAJC6 KD in astrocytes exacerbates α-syn pathology in the PD triculture model. (**A**) Schematic outlining the experimental procedure. mDA neurons overexpressing α-syn were cocultured with astrocytes and microglia and exposed to α-syn PFFs. CasRx-mediated DNAJC6-KD astrocytes or control astrocytes (mock transduced without the gRNA) were included in the PD culture model. (**B**) The pathological effect of DNAJC6-KD astrocytes was determined by analyzing the levels of high-molecular-weight α-syn aggregates in Triton X-100–soluble and –insoluble (SDS soluble) fractions. (**C** and **D**) Misfolded α-syn aggregation propensity assessed by the percentage of Thioflavin S/TH-costained cells (**C**) and the percentage of pS129-α-syn^+^/TH^+^ cells (**D**). (**E**) mDA neurite degeneration assessed by TH^+^ fiber shortening. (**F**) Synaptic degeneration assessed by the number of abnormal synaptic vesicle clusters (>5 μm SYN1^+^ clumps). Scale bars: 50 μm (**C**–**F**). (**G**) The viability of astrocytes and microglia was assessed by quantifying GFAP^+^ or AIF1^+^/DAPI cells. Scale bar: 50 μm. *n* = 3 (**B**), 8 (**C**), or 4 (**D** and **G**) independent experiments or 4–8 cells/experiment (*N* = 3 independent experiments; **E** and **F**); *P < 0.05, **P < 0.001; paired 2-tailed *t* test (**B**), unpaired 2-tailed *t* test (**C**, **D**, and **G**), or nested 2-tailed *t* test (**E** and **F**).

To assess astrocytic DNAJC6 function, CasRx-mediated DNAJC6-KD astrocytes (or mock-transduced controls) were incorporated into the PD triculture system (Figure 5A). Cultures containing DNAJC6-KD astrocytes exhibited significantly increased high-molecular-weight α-syn aggregates in the Triton-insoluble fraction (WB) (Figure 5B) and more neurons harboring pathological α-syn aggregates (Thioflavin S^+^ and pS129-α-syn^+^/TH^+^) (ICC) compared with controls (Figure 5, C and D). Astrocytic DNAJC6 KD further worsened α-syn–induced mDA degeneration, as evidenced by TH^+^ neurite shortening (Figure 5E) and enhanced synaptic vesicle clustering (Figure 5F). Although glial cells are typically resistant to cell loss, they often become functionally impaired under pathological conditions. Consistently, astrocyte and microglia numbers remained unchanged under α-syn and DNAJC6-KD conditions (Figure 5G), indicating that mDA neurodegeneration results from glial functional alterations rather than reduced glial abundance.

Next, we dissected the contribution of astrocytic DNAJC6 by examining its effects on DA neurons and microglia separately. Similar, though comparatively weaker, effects of astrocytic DNAJC6 KD were observed in mDA neuron–astrocyte cocultures lacking microglia (Supplemental Figure 13). When hESC-derived microglia were cocultured with DNAJC6-KD astrocytes, we observed a marked increase in IL-1β and p21 (CDKN1A) expression — representing a proinflammatory cytokine and senescence marker, respectively — in the microglial cells (Supplemental Figure 14A), as well as elevated IL-1β and TNF mRNA levels in the microglia–astrocyte cocultures compared with those cocultured with control astrocytes (Supplemental Figure 14B). Furthermore, treatment of microglia with conditioned media collected from DNAJC6-KD astrocytes similarly increased the expression of proinflammatory and senescence markers (Supplemental Figure 14C). Together, these findings suggest that the astrocytic DNAJC6-dependent effects observed in the triculture system arise from both direct protection of DA neurons and indirect protection via suppression of microglial inflammatory activation.

### Therapeutic effects of astrocytic or neuronal CRISPRa-DNAJC6 expression in in vitro PD models.

**Figure 6:**
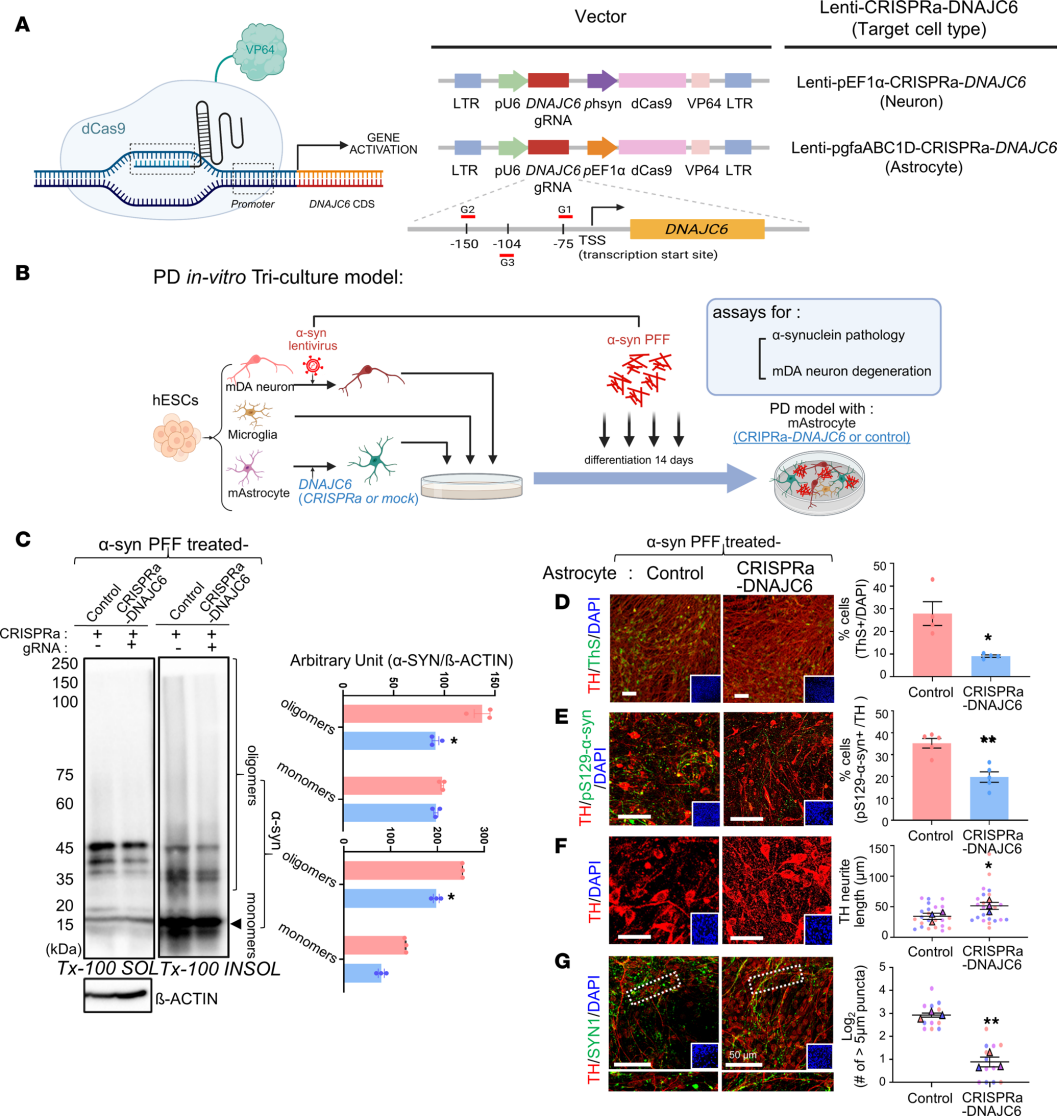
Astrocytic CRISPRa-DNAJC6 expression ameliorates α-syn-PD pathologies in an in vitro PD model. (**A** and **B**) Schematic of the dCas9-VP64 transactivator-mediated CRISPRa-DNAJC6 overexpression system (**A**) and the experimental procedure (**B**). (**C**–**E**) Pathological misfolded α-syn aggregation analyzed by α-syn WB using Triton X-100–soluble and –insoluble fractions (**C**) and ICC for the percentage of Thioflavin S–stained cells (**D**) and pS129-α-syn^+^/TH^+^ cells (**E**). (**F** and **G**) mDA neuron degeneration assessed by TH^+^ neurite length (**F**) and synaptic vesicle clustering, measured as the number of SYN1^+^ clumps > 5 μm (**G**). Scale bars: 50 μm (**D**–**G**). *n* = 3 (**C**), 4 (**D**), or 6 (**E**) independent experiments or 4–8 cells/experiment (*N* = 3 independent experiments; **F** and **G**); *P < 0.05, **P < 0.001; paired 2-tailed *t* test (**C**), unpaired 2-tailed *t* test (**D** and **E**), or nested 2-tailed *t* test (**F** and **G**).

The functions of DNAJC6 suggest that restoring its expression may represent a therapeutic strategy for PD. However, supraphysiologic expression using a pEF1α-driven lentiviral system (Supplemental Figure 15, A and B) induced cell death (Supplemental Figure 15C). To achieve physiologic upregulation, we applied a CRISPRa approach to activate the endogenous human DNAJC6 promoter via dCas9-VP64 (Figure 6A). This strategy highlights the potential of CRISPR/Cas9-based epigenetic regulation as a next-generation gene therapy approach with reduced side effects (41, 42).

Compared with control α-syn-PD tricultures (astrocytes transduced with dCas9-VP64 alone without DNAJC6 gRNA constructs) (Figure 6, A and B), pathological α-syn aggregates were significantly reduced in tricultures containing astrocytes expressing CRISPRa-activated DNAJC6, as shown by WB (Figure 6C) and ICC (Figure 6, D and E). Moreover, TH^+^ mDA neurite loss and SYN1^+^ synaptic clustering observed under α-syn pathology were markedly attenuated in cultures with CRISPRa-DNAJC6 astrocytes (Figure 6, F and G).

Beyond astrocytes, DNAJC6 is essential for neuronal homeostasis (7, 10–12), yet neuron-specific DNAJC6 expression is reduced under PD conditions (Figure 3), indicating that neuronal restoration may also be required. Accordingly, α-syn-PD tricultures were generated with either CRISPRa-DNAJC6–expressing neurons or mock-transduced controls (Supplemental Figure 16A). Neuron-specific CRISPRa-DNAJC6 significantly rescued α-synucleinopathy and mDA neuron/synaptic degeneration (Supplemental Figure 16, B–E). Combined overexpression in both neurons and astrocytes showed a trend toward greater reduction of α-syn aggregation than single–cell type targeting, although this did not reach statistical significance (Supplemental Figure 17).

### CRISPRa-mediated activation of DNAJC6-treated PD symptoms and pathologies in α-syn PD mouse models.

**Figure 7:**
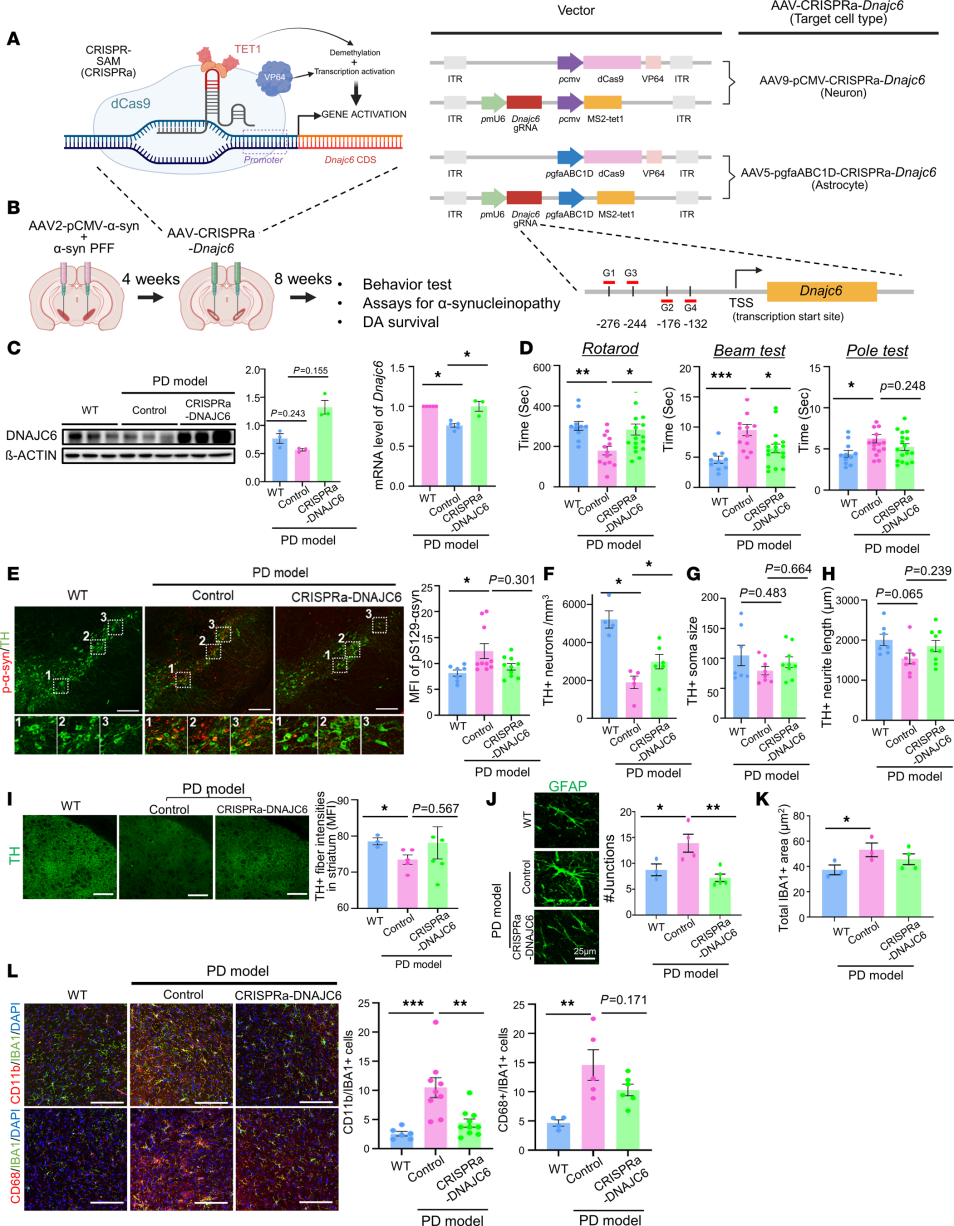
DNAJC6 overexpression by CRISPR-SAM (a CRISPRa system) rescues motor deficits and pathological changes in the α-syn-PD mouse model. (**A**) Schematic of the CRISPRa system, illustrating sgRNA-guided DNAJC6 upregulation via demethylase (TET1) and dCas9 fused with the transcriptional activator VP64, delivered using astrocyte- and neuron-targeting AAV vectors. (**B**) Experimental timeline depicting the in vivo study design. α-Syn-PD models were generated by infecting the midbrain SN with AAV2- or AAV9-α-syn and α-syn PFF, followed by an injection of AAV9 carrying CRISPRa-DNAJC6 1 month later. (**C**) WB-based determination of DNAJC6 protein expression (left) or mRNA expression of DNAJC6 (right) in the mouse SNs (*n* = 3–4 independent experiments). (**D**) Behavioral assessments were conducted 2 months after AAV-CRISPRa-DNAJC6 administration. Rotarod, beam, and pole tests were performed (*n* = 3–4 independent experiments/animal). (**E**) Representative immunofluorescence images of dopaminergic neurons in the SN, stained for TH (green) and phosphorylated α-syn (pS129 α-syn, red), comparing WT, PD model, and CRISPRa-AAV-DNAJC6–treated PD model mice. The graph shows the percentage of pS129 α-syn^+^ neurons and TH^+^ neurons in the SN in WT, PD model, and PD model with CRISPRa (*n* = 3–6 independent experiments/animal). (**F**–**H**) Quantification of TH^+^ neuron number (**F**), soma size (**G**), and neurite length (**H**). (**I**) TH^+^ immunoreactivity in the striatum. Quantification of TH intensity is shown in the graph. (**J** and **K**) Morphometric analysis of astrocytes (**J**) and microglia (**K**). More detailed analysis is shown in Supplemental Figure 18. (**L**) Representative image of microglia immunostained with IBA1 (green), CD11b (red), and CD68 (red) in SN in WT, PD model, and PD model with CRISPRa. Quantification of AIF1^+^ microglia colocalized with activation markers, ITGAM or CD68 in SN in WT, PD model, and PD model with CRISPRa. *N* = 3–9 animals/group. *P < 0.05, **P < 0.01, ***P < 0.001; 1-way ANOVA, with Bonferroni’s post hoc analysis. Scale bars: 100 μm (**E**, **I**, and **L**), 25 μm (**J**).

Building on our in vitro findings, we examined whether epigenetic activation of DNAJC6 could mitigate pathology and behavioral deficits in an α-syn–induced PD mouse model. Human α-syn PFFs together with adeno-associated virus-mediated (AAV-mediated) overexpressing human SNCA were injected into the SN (43, 44). Due to species differences in DNAJC6 promoters, we redesigned the CRISPRa system with gRNAs targeting the mouse DNAJC6 promoter (Figure 7A). To enhance activation efficiency, we used the CRISPR-based synergistic activation mediator (SAM) system (dCas9-VP64, MS2-TET1, and gRNAs) (Figure 7A). Because DNAJC6 is required for homeostasis in both astrocytes and neurons and is downregulated in PD, we targeted both cell types. AAV5-pgfaABC1D efficiently transduced astrocytes, whereas AAV9-CMV preferentially targeted neurons in the midbrain (45). Accordingly, AAV5-pgfaABC1D-CRISPRa-DNAJC6 and AAV9-pCMV-CRISPRa-DNAJC6 were stereotaxically injected into the SN of α-syn-PD mice (Figure 7B). Reduced DNAJC6 expression in PD mice was restored by AAV-CRISPRa-DNAJC6, as confirmed by qPCR and WB (Figure 7C). Cell type–specific expression could not be verified due to the lack of an IHC-compatible mouse DNAJC6 antibody.

Behavioral assessments performed 2 months after CRISPRa injection (3 months after α-syn-AAV) (Figure 7D) showed significant improvement in rotarod and beam tests, with a trend toward improvement in the pole test.

The midbrain SN of α-syn–injected PD mice was manifested by α-syn aggregates accumulated in mDA neurons (measured as fluorescence intensity of pS129-α-syn in TH^+^ cells). However, these aggregates were significantly reduced in the SN of PD mice treated with AAV-CRISPRa-DNAJC6 (Figure 7E). Compared with untreated mice (5,286 ± 480 cells, *n* = 4), TH^+^ mDA neurons in the SN of α-syn-PD mice were markedly reduced to 1,897 ± 336 cells (*n* = 5) (Figure 7F). However, epigenetic activation of DNAJC6 using the CRISPRa system significantly mitigated mDA neuronal loss, increasing the number of TH^+^ neurons to 2,986 ± 355 cells (*n* = 6, *P* < 0.05). In addition, surviving TH^+^ mDA neurons in the SN of PD mice exhibited small cell bodies (Figure 7G) with blunted and fragmented neurites (Figure 7H), indicative of neurodegeneration. In contrast, mDA neurons in the SN of PD mice treated with CRISPRa-DNAJC6 appeared healthier, displaying larger cell bodies and extended neurite outgrowths. Consistently, AAV-CRISPRa-DNAJC6 administration substantially preserved nigrostriatal dopaminergic innervation, as evidenced by the increased TH^+^ fiber intensity in the striatum (Figure 7I).

Astrocytes and microglia in α-syn-PD SN showed hypertrophic, proinflammatory morphologies (Figure 7, J and K, and Supplemental Figure 18), with elevated ITGAM and CD68 expression in AIF1^+^ microglia (Figure 7L). These inflammatory changes were markedly reduced by AAV-CRISPRa-DNAJC6 treatment (Figure 7, J–L, and Supplemental Figure 18). Thus, restoring a healthier midbrain environment — through reduced synucleinopathy and neuroinflammation — likely underlies the preservation of mDA neurons and improved motor function.

Importantly, CRISPRa-mediated DNAJC6 overexpression in WT mice did not alter behavior or SN histology (Supplemental Figure 19), supporting the safety of this approach. Collectively, these findings support the therapeutic potential of CRISPRa-based epigenetic activation of DNAJC6 in sporadic PD.

## Discussion

DNAJC6, harboring loss-of-function mutations in juvenile-onset PD, is a brain-specific HSP40 cochaperone essential for CME. Our recent work showed that early-onset phenotypes are linked to neurodevelopmental defects, where impaired DNAJC6-dependent endocytosis in neural precursors disrupted WNT signaling required for mDA neuron development (10). Beyond development, DNAJC6-mediated clathrin uncoating is critical for synaptic vesicle recycling and lysosomal trafficking in adult neurons — processes central to PD pathogenesis (46–48). However, DNAJC6-KO mouse studies have yielded inconsistent results, ranging from absent PD phenotypes (7, 11, 49) to clear PD-like manifestations (50, 51). Notably, Ng et al. (50) observed pronounced PD-associated manifestations early in life, resembling juvenile-onset parkinsonism previously reported in families with DNAJC6 mutations (7–9). In contrast, Vidyadhara et al. (51) documented progressive PD symptoms and pathologies in older DNAJC6-KO mice, resembling the course of sporadic PD. Considering the potential roles of DNAJC6 in adult neuronal cells and the recent animal data, we hypothesize that DNAJC6 dysfunction not only pertains to juvenile PD but likely encompasses a pathogenic mechanism for late age–onset sporadic PD.

We demonstrate that DNAJC6 is downregulated in postmortem sporadic PD brains and in multiple in vitro PD models. Importantly, DNAJC6 is highly expressed in astrocytes and similarly reduced in PD contexts. Astrocytes provide essential neurotrophic and homeostatic support but can adopt proinflammatory phenotypes in disease (52–55). We show that astrocytic DNAJC6 is required for cellular homeostasis: its KD impaired endocytosis and lysosomal trafficking, disrupted autophagy/mitophagy, induced mitochondrial damage, and activated MAVS/TBK1/IRF3/NLRP3 inflammatory signaling. Consequently, astrocytes acquired a neurotoxic phenotype that promoted mDA degeneration in coculture. These findings align with previous reports linking astrocytic autophagy defects and mitochondrial dysfunction to neuroinflammation and neurodegeneration (56–58).

Mechanistically, reduced Nurr1/Foxa2-mediated transcription underlies DNAJC6 downregulation in sporadic PD. Nurr1 and Foxa2 are essential for mDA neuron survival and also mitigate glial inflammation (14–17, 59–62), yet are diminished in toxic PD environments (16–19). We show that Nurr1/Foxa2 directly bind the DNAJC6 promoter, and their reduced recruitment under α-syn pathology decreases DNAJC6 transcription. Additionally, consistent with prior work (22), LRRK2 mutations promoted DNAJC6 degradation via autolysosomal and proteasomal pathways. Nurr1/Foxa2 loss and LRRK2 hyperactivation converge on autophagy, mitochondrial dysfunction, and inflammation (14–16, 60, 63, 64); thus, our data suggest that DNAJC6 may act as a key downstream mediator of these PD-linked pathways.

Finally, restoring and enhancing DNAJC6 expression effectively alleviated PD symptoms and pathologies in both α-syn–induced cell cultures and mouse models. These benefits were achieved by activating the endogenous DNAJC6 promoter via CRISPRa. Notably, traditional overexpression proved harmful, highlighting the risk of supraphysiological gene levels in current gene therapies. Thus, maintaining expression within physiological limits is crucial for safety and efficacy. This can be managed by fine-tuning AAV vector titers and incorporating negative feedback mechanisms such as transcriptional repressors or microRNAs that suppress excess expression. Among these, miR-3120 is a strong candidate, targeting conserved sites in the DNAJC6 3′ UTR (65). Coupling CRISPRa activation with a miR-3120–based feedback loop could establish a self-regulating system that maintains near-physiological protein levels.

Our findings also suggest that other CME-related proteins may exert neuroprotective effects. GAK (Auxilin-2) is upregulated in DNAJC6-KO mice and prevents PD-like phenotypes (11). Supporting this and consistent with astrocytic DNAJC6 functions in this study, recent *Drosophila* studies show GAK functions in glia to clear α-syn aggregates via enhanced autophagy and lysosomal activity (66, 67). SYNJ1, another key CME regulator and phosphoinositide phosphatase, is implicated in early-onset PD through loss-of-function mutations (68); SYNJ1-deficient mice exhibit dopaminergic axonopathy that parallels or synergizes with DNAJC6 loss (50), highlighting functional convergence within CME pathways. Transcriptomic analyses (Supplemental Figure 2) also indicate altered expression of several chaperones in PD, some of which may interact with DNAJC6. However, in our in vitro and in vivo α-syn-PD models, no significant changes in chaperone expression were observed, except for DNAJC6 (Figure 2C and Supplemental Figure 20). Furthermore, restoring DNAJC6 expression in these PD models did not alter the significant expression of other chaperone proteins, indicating its rescue effect is largely independent of broader chaperone modulation.

Notably, DNAJC6 dysregulation occurs not only under PD-specific conditions (e.g., α-syn aggregation and LRRK2 hyperactivation) but also in response to general neurotoxic insults such as mitochondrial dysfunction, oxidative stress, and inflammation (Figure 1G), suggesting its role extends beyond PD. Consistently, proteomic studies in Alzheimer’s disease also report chaperone dysregulation (69, 70), including significant DNAJC6 downregulation across multiple brain regions (70). These findings support a broader pathological relevance of DNAJC6 dysfunction across neurodegenerative diseases, although the mechanistic contributions remain to be elucidated.

In summary, we provide evidence that DNAJC6, a central CME regulator, may be an important mediator of sporadic PD pathogenesis and a promising therapeutic target. Astrocytic DNAJC6 dysregulation emerged as a key driver of disease mechanisms. Future studies should explore cooperative chaperone networks in neurons and astrocytes to uncover additional therapeutic targets for PD and related neurodegenerative diseases.

## Methods

Additional details may be found in Supplemental Methods.

### Sex as a biological variable.

Midbrain tissues for IHC analysis were obtained from male and female patients with sporadic PD, along with age-matched control subjects (Supplemental Table 5). All animal experiments were conducted using female mice. Females were selected to minimize aggression, stress-related variability, and injury associated with group housing and longitudinal behavioral testing in this α-syn PD model. The study was not designed or powered to evaluate sex-specific effects. Although PD affects both sexes, the molecular mechanisms examined here are not known to be restricted to sex, and we expect the principal findings to be broadly relevant.

### hESC and hiPSC culture.

hPSCs were maintained feeder-free on Matrigel (BD Biosciences) in mTeSR1 (Stemcell Technologies) with doxycycline (1 μg/mL) (71), with daily medium changes. Cell lines are listed in Supplemental Table 1. Cells were passaged every 3–6 days (1:3–1:6) using Accutase (Stemcell Technologies). ROCK inhibitor Y-276232 (10 μM) was applied for 24 h after passaging or thawing. H9 hESCs were from WiCell; LRRK2 isogenic iPSCs were from Mark Cookson (National Institute on Aging Bethesda, Maryland, USA) (72). Mycoplasma testing was performed using the e-Myco PCR kit (LiliF).

### Preparation of DA neuron- and astrocyte-enriched cultures from hPSCs.

hPSCs were differentiated into ventral midbrain organoids, and organoid-derived NSCs were isolated as described (13). Organoid-derived NSCs were expanded in basic FGF (10 ng/mL), EGF (10 ng/mL), and ascorbic acid (200 μM). Early-passage NSCs (P1–P2) were terminally differentiated into DA neuron–enriched cultures in N2 medium containing brain-derived neurotrophic factor (BDNF), glial cell line–derived neurotrophic factor (GDNF) (10 ng/mL each), ascorbic acid (200 μM), and dibutyryl cAMP (db-cAMP) (500 μM). Late-passage NSCs (P15–P22; 150- to 200-day expansion) were differentiated into astrocytes by mitogen withdrawal for 15 days (36).

### Derivation of microglia from hESCs.

Microglia were generated via an hESC-macrophage organoid protocol (73). H9 hESCs were seeded (10,000 cells/well) in low-attachment plates with BMP4, VEGF-A, SCF, and ROCK inhibitor Y276232 (10 μM) on day 0. Y276232 was used only for the first 24 h, and the medium was changed every day until day 3. From day 4, 18–20 organoids were moved to 1 well of an ultra-low-binding 6-well plate with hematopoietic medium (Xvixo-15, LONZA) containing GlutaMAX (Gibco) and β-mercaptoethanol with cytokines (SCF, M-CSF, IL-3, and FLT3). Macrophage progenitors (days 11–17) were collected and differentiated into microglia in N2 medium supplemented with M-CSF and IL-34 for ≥2 weeks.

### Triple culture of neurons, astrocytes, and microglia (in vitro α-syn PD model).

mDA neurogenic NSCs (passages P2–P4), astrogenic NSCs (P8–P10), and microglia-like cells — each derived from hPSCs, including hESCs or hiPSCs — were plated in a 2:1:1 ratio onto poly-l-ornithine– and fibronectin-coated plates or glass coverslips. Cells were maintained in N2 expansion medium supplemented with 10 ng/mL basic FGF, 10 ng/mL EGF, and 200 μM ascorbic acid for 4 days. Terminal differentiation was induced by switching to differentiation medium containing N2, 10 ng/mL BDNF, 10 ng/mL GDNF, 100 ng/mL IL-34, 10 ng/mL macrophage CSF (M-CSF), 200 μM ascorbic acid, and 25 μM db-cAMP for 2–3 weeks, yielding a triculture of mDA neurons, astrocytes, and microglia. For in vitro PD modeling, mDA neurogenic NSCs were transduced with lentiviruses expressing human α-syn (pEF1α-α-syn) before coculture, following the same protocol. The resulting tricultures, containing α-syn–overexpressing neurons, astrocytes, and microglia, were exposed to 2 μg/mL α-syn PFFs for 14 days. Immunocytochemical analysis was performed as detailed in Supplemental Methods. Immunofluorescence staining was imaged using a confocal microscope (LSM900, Zeiss) at the Biospecimen-Multiomics Digital Bioanalysis Core Facility of Hanyang University.

### Virus production.

Lentiviruses and AAVs (serotype 2, 5, or 9) expressing genes of interest under the control of the EF1α promoter (pEF1α), neuron-specific SYN1 promoter (pSYNI), or astrocyte-specific GFAP promoter (pgfaABC1D) were generated as previously described (74). Vectors are listed in Supplemental Table S2.

### DNAJC6 KD using CRISPR/CasRx.

Lentiviral vectors for knocking down human DNAJC6 were modified from the lenti-EF1a-dCas9-KRAB-Puro vector obtained from Addgene (plasmid 99372), with the puromycin cassette replaced by copGFP. The copGFP protein was simultaneously expressed with dCas9 via a self-cleaving 2A peptide (T2A). The sgRNA was designed to target the DNA region from −50 to 300 bp relative to the transcription start site of the *DNAJC6* gene using the GPP web tool (https://portals.broadinstitute.org/gpp/public/analysis-tools/sgrna-design-crisprai). sgRNA cassettes were combined with the U6 promoter by utilizing overlap-extension PCR, with addition of *Nhe*I and *Asc*I restriction sites at 5′ and 3′ and cloned upstream of EF1-dCAS9-KRAB-CopGFP to generate a U6-hsgDNAJC6- EF1-dCAS9-KRAB-CopGFP vector.

### Preparation of human α-syn PFFs.

Recombinant human α-syn and α-syn PFFs were prepared following the methods previously described (75). Briefly, 5 mg/mL of monomeric α-syn was incubated at 37°C with continuous agitation at 100*g* for 7 days, sonicated on ice for 3–5 seconds at 3 W using a VC 505 ultrasonic processor (Sonics & Materials), and stored at −80°C until used as α-syn PFF. The formation of α-syn fibrils was confirmed using the thioflavin T binding assay and transmission electron microscopy (Zeiss).

### Preparation of PD mouse models.

PD model mice were generated by the combined treatment with AAV2 or AAV9 expressing human SNCA (pSNCA-α-syn-AAV2 or -AAV9) and α-syn PFFs (43). To this end, mice (female, 2.5 months old) were anesthetized using Zoletil 50 (0.1 mg/kg, Virbac) mixed with Rompun (93.28–l g/kg, Elanco), and α-syn PFF (2 μL, 5mg/mL in PBS) and α-syn-AAV (2 μL, 2.1 × 10^12^ GC/mL) were injected bilaterally into the SN (3.3 mm posterior to bregma; ±1.2 mm lateral to midline; –4.6 mm ventral to dura) (the same volume of PBS injected for control). The infusion was performed at a rate of 0.25 μL per minute. The needle (26 gauge) was left in the injection site for 25–30 minutes after completion of each injection and then removed slowly.

### DNAJC6 overexpression in the SN of PD mice.

One month after α-syn AAV+PFF injection, the mixtures of the AAV5 carrying pgfaABC1D-DNAJC6-CRISPRa (for targeting astrocytes, 4 × 10^13^ GC/mL, 1 μL) and AAV9 carrying pCMV-DNAJC6-CRISPRa (for targeting neurons, 8 × 10^13^ GC/mL, 1 μL) (or PBS as control) were stereotaxically injected into the SN of the α-syn-PD mice. Three months after α-syn injection, the gain-of-function effect of DNAJC6 was evaluated by a series of behavioral tests.

### Motor behavior assays.

To evaluate PD-associated motor dysfunction, the following behavioral assays were performed using 3 trials after 2–3 days of training.

For the rotarod test, motor coordination and balance were measured by placing the animal on a rotating rod with accelerating rotation (4–44 rpm speed, 300 s). The time the animal stayed on the rod was measured. If the animal succeeded for the full test, it was given the maximum score (300 s, 300 score).

For the beam test, motor coordination and balance were analyzed by placing the animal on a beam (square, 80 cm in length, 12 mm in diameter, 50 cm above the ground) and measuring the time required to traverse it. If the animal fell from the beam, it was given the highest value of the week.

For the pole test, animals were placed on top of a vertical pole (50 cm long wooden pole, 1 cm in diameter) with their heads downward. The time taken to orient downward was measured in 3 trials. If the animal fell or slipped from the pole, it was given the highest value of the week.

### Statistics.

In the culture wells (coverslips), immunostained and DAPI-stained cells were counted in 9–20 random areas using an eyepiece grid at a magnification of ×200 or ×400, and total positive cells (or percentages) in a well were calculated. Mouse brains were cryosectioned, and every 5 sections were stained and counted for immunopositive cells out of DAPI. An Abercrombie correction factor was applied. In the figures, data are expressed as mean ± SEM. To check whether the data were normally distributed, a Shapiro-Wilk test was performed. For comparing 2 groups, a 2-tailed *t* test was used when the data were normally distributed, and Mann-Whitney *U* test was used if the data were not normally distributed. For comparing more than 2 groups, 1-way ANOVA was used when the data were normally distributed, and Brown-Forsythe test or Welch’s test was used if the data were not normally distributed. Post hoc comparisons were performed with Bonferroni’s, Tukey’s, or Dunn’s test. *P* values of less than 0.05 were considered significant. The sample size and the statistical tests used for each figure are described in the figure legends. Statistical tests were conducted using GraphPad Prism software version 9.

### Study approval.

Animal experiments were approved by the IACUC of the College of Medicine, Hanyang University (approval numbers 2021-0086A, 2021-0215A, 2022-0085A, and 2024-0035A). Experiments adhered to NIH guidelines.

### Data availability.

Publicly available RNA-seq datasets analyzed in this study are available in the Gene Expression Omnibus (GEO) under accession numbers GSE49036, GSE68719, GSE208783, and GSE207713 and in the European Nucleotide Archive under accession number ERP129142. Publicly available microarray datasets are available in GEO under accession numbers GSE54086 and GSE145489. Values for all data points in graphs are reported in the Supporting Data Values file.

## Author contributions

WHWD, YH, and NW performed in vivo experiments and data analyses. WHWD, YH, EV, and LTG performed cell culture experiments and data analysis. WHWD and NW performed scRNA-seq data analysis. SHL wrote the manuscript. SJH, HR, and TDS analyzed brain samples from patients with PD. MYC revised the manuscript. MYC and SHL conceptualized, designed, and supervised the study.

## Conflict of interest

The authors have declared that no conflict of interest exists.

## Funding support

- Korea-US Collaborative Research Fund (KUCRF) grant RS-2024-00468036 (to SHL and MYC).
- National Research Foundation of Korea under the Ministry of Science and ICT, South Korea, grants RS-2023-00216127 (to SHL and MYC), RS-2024-00350753 (to SHL), RS-2022-NR069738 (to MYC), RS-2020-NR046283 (to SHL), and 2022R1A2C3013138 (to HR).

## Supplementary Material

Supplemental data

Unedited blot and gel images

Supporting data values

## Version 1 04/09/2026

In-Press Preview

## Version 2 06/01/2026

Electronic publication
