# Supplementary material for: Dysregulation of astrocytic DNAJC6 contributes to sporadic Parkinson’s disease pathogenesis
Source: J Clin Invest. 2026 Apr 9;136(11):e194989. doi: 10.1172/JCI194989 (PMC13221221; doi:10.1172/JCI194989)
Supplement: Supplemental data [file jci-136-194989-s008.pdf]

Supplementary Materials and Methods

Chromatin immunoprecipitation-quantitative PCR (ChIP-qPCR)

NURR1 and FOXA2 binding sites in the human DNAJC6 promoter were identified using the Jaspar database (http://jaspar.genereg.net) and an 80% score threshold. Predicted NURR1 and FOXA2 binding sites were coupled with phylogenetic footprinting to eliminate spurious predictions with specified position weight matrix (PWM) settings. ChIP assays were carried out as previously described (1). Briefly, chromatin was sheared to an average of 200–500 base pairs (bp) using a sonicator (Bioruptor-BMS, Seoul, Korea) and immunoprecipitated using the antibodies listed in Supplementary Table S3. Immunoprecipitated DNA fragments were collected using magnetic beads (Invitrogen), purified, and subjected to real-time PCR using the primers listed in Supplementary Table S4. The comparative cycle threshold method was used to quantify the results. Data were normalized to the input DNA.

Endosomal uptake assay using FM 1-43 styryl dye

Cells were initially rinsed and then exposed to HBSS supplemented with Ca2+ for 10 minutes at 37°C. Subsequently, the buffer was removed, and the cells were treated with HBSS containing Ca2+ and 60 mM KCl, supplemented with 5 μg/mL FM 1-43FX styryl dye (Thermo Fisher), for 1 minute at 37°C. Following this, cells were further incubated in HBSS with Ca2+ and 5 μg/mL FM 1-43FX styryl dye for an additional 15 minutes at 37°C, swiftly washed twice with PBS, and left to incubate overnight at 4 °C. The following day, cell fixation was carried out using 3% PFA for 20 minutes, followed by mounting in Vectashield medium containing DAPI (Vector Laboratories, West Grove, PA). Imaging was performed utilizing a confocal microscope.

Auto-lysosomal assays

Auto-lysosomal activity was determined by WB-based determination of the protein levels for LC3 and p62 in the absence and presence of the lysosomal inhibitor Bafilomycin A (Selleck Chem. LLC, Houston, TX). Bafilomycin A was applied for 48hr after 7 days of differentiation.

Mitochondrial Assay

MitoTimer analysis was performed by transducing cells with lentiviruses carrying the mitotimer vector (mitochondrial-targeting sequences of the human cytochrome *c* oxidase subunit VIII gene fused with a timer-reporter gene (DsRed1-E5)) (Addgene #52659). Mitophagy analysis using MitoKeima was performed by transducing cells with lentivirus carrying mitochondrial-targeting sequence of the human cytochrome c oxidase subunit VIII (Cox8) gene fused with coral-derived protein Keima (2). Mitochondria ROS was evaluated using MitoSOX Red (Invitrogen, M36008) as described previously(1).

GBA Lysosomal Activity Assay

Lysosomal GBA activity was measured as described previously (1) with slight modifications. Cells were collected in ice-cold lysosome isolation buffer, homogenized through a 27-gauge syringe, and centrifuged at 1,000 × g for 10 min (4 °C). The post-nuclear supernatant was combined with enrichment buffer and centrifuged to obtain lysosomal pellets. 10 µg lysosomal protein was incubated with 1 mM 4-MUG in assay buffer (0.25% sodium taurocholate, 0.1 M sodium citrate, pH 5.6) at 37 °C for 2 h. Reactions were stopped with glycine buffer (pH 10) and fluorescence recorded (Ex 355 nm/Em 460 nm). CBE-sensitive activity was determined after 24 h pre-incubation with 50 µM conduritol-B-epoxide, and relative GBA1 activity calculated by subtracting CBE-treated from untreated samples.

Cytosolic mitochondrial DNA (mtDNA) determination

Isolation and quantification of mitochondrial DNA in cytosolic extract and whole cell extract (WCE) were performed as previously described (3). Measure the DNA concentration of each fraction. Purity tests of each fraction were done by performing western blot of each fraction against GAPDH (cytosolic extract), TFAM (mitochondrial extract), and Lamin (nuclear extract). If fractions are free of cross-contamination as determined by western blot analysis, 2 ng/µL in nuclease-free water were subjected to qPCR analysis. Calculation of mtDNA abundance relative to nuclear DNA were performed using the delta delta Cq (ΔΔCq) method.

Detection of MAVS Aggregation

MAVS aggregation was analyzed using semi-denaturing detergent agarose gel electrophoresis (SDD-AGE), as previously described (4). Briefly, mitochondrial extracts were resuspended in 1×

sample buffer (0.5× TBE, 10% glycerol, 2% SDS, and 0.0025% bromophenol blue) and loaded onto a vertical 1.5% agarose gel. Electrophoresis was performed at 100 V for 35 minutes at 4°C using running buffer (1× TBE, 0.1% SDS). Proteins were then transferred to an Immobilon membrane (Millipore) for immunoblotting with a MAVS antibody (Supplementary Table S3). Additionally, MAVS aggregates were detected via immunofluorescence staining using the MAVS antibody (Supplementary Table S3), and MAVS-stained puncta were quantified.

Western blot analysis

Total cellular proteins were extracted in a RIPA buffer containing protease inhibitor (Roche) and phosphatase inhibitor cocktails (Sigma). Mitochondrial proteins were prepared as previously described (3). To detect aggregate forms of α-syn, protein was extracted from cultured cells using a 1% Triton X-100/PBS buffer containing a protease inhibitor (Roche) and phosphatase inhibitor cocktails (Sigma). After centrifugation (16,000 g, 10 min), the Triton X-100 supernatant was collected (Triton X-100-soluble). The pellet was dissolved in 1% SDS sample buffer and then briefly sonicated (Triton X-100-insoluble protein, SDS-soluble protein). Protein samples (15–40 μg of protein) were electrophoresed on SDS-PAGE gels (6–20% gradient), transferred to a nitrocellulose membrane, blocked with 5% BSA/PBS, and then incubated with primary antibodies (overnight , 4℃) (listed in Supplementary Table S3). The next day, the secondary antibodies treatment occurred for 1 h. Signals were captured with ChemiDoc (Bio-Rad Laboratories) and quantification was performed using ImageJ (NIH, Bethesda, MD).

For immunoprecipitation, cell lysates from the triple *in-vitro* PD model were used. Protein concentration was determined using a BCA assay and normalized to 2 mg/ml prior to the IP experiments. Each sample was incubated with an anti-LRRK2 (ab181386; Abcam) antibody and the Co-immunoprecipitation (Co-IP) was performed using the Thermo Scientific Pierce Crosslink IP/Co-IP Kit (Cat. # 88,805) following the manufacturer's protocol.

Proximity ligation assay (PLA)

Protein interactions were detected using the Duolink® PLA kit (Merck) following manufacturer protocols and analyzed by confocal microscopy.

RNA extraction, reverse transcription, and qRT-PCR

Total RNAs were isolated using TRIzol Reagent (Invitrogen), and reverse-transcribed using the SuperScript III kit (Invitrogen) to make cDNA. Transcript abundance was determined by qRT-PCR using SYBR Green PCR mix (Bio-Rad) and performed using CFX Connect Real-Time System (Bio-Rad). The ΔΔCq method was used to normalize expression levels of each gene to that of GAPDH. Primers used are listed in Supplementary Table S4.

Immunofluorescence staining for cultured cells and mouse brain sections

Cultured cells were fixed with 4% paraformaldehyde (PFA) in PBS for 20 min, washed, and blocked in 0.3% Triton X-100 with 1% BSA for 40 min. Mice were anesthetized and perfused transcardially with 4% PFA. Brains were removed and immersed in 30% sucrose in PBS until sank, frozen in Tissue-Tek (Sakura Finetek), and sliced (30 μm thick) on a freezing microtome (Leica). The culture cells and brain sections were incubated with primary antibodies at 4°C overnight, followed by incubation with secondary antibodies for 1 h at room temperature. Primary and secondary antibody information is shown in Supplementary Table S3. The stained cells /sections were mounted with Vectashield (Vector Laboratories, Burlingame, CA, USA) and DAPI mounting solution. Images were obtained using an epifluorescence microscope (Leica) or confocal microscope (Leica TCS SP5 confocal).

Analysis of Astrocyte and Microglia Morphology

Morphological analysis was performed using Fiji (v1.54). Maximum-intensity Z-projections were thresholded to create binary masks for each cell, from which cell area and circularity were measured. Skeletonized images were analyzed with the Analyze Skeleton plugin to quantify branching morphology. For each mouse, a minimum of 15 cells was analyzed.

Immunostaining of Human Postmortem Brain Tissue

Postmortem brain samples from normal and Parkinson's disease (PD) subjects were generously provided by the Boston University Alzheimer's Disease Research Center (BUADRC) (more information for in Supplementary Table S5). The brain tissues were embedded in paraffin and sectioned coronally at a thickness of 20 μm. Sections were deparaffinized using three consecutive xylene baths (5 minutes each) and then rehydrated through a graded ethanol series (100%, 95%,

80%, and 60%, each for 5 minutes). To quench endogenous peroxidase activity, sections were incubated with 3% hydrogen peroxide in TBS for 15 minutes at room temperature. To detect DNAJC6 expression, sections were blocked with 5% goat serum in PBS for 1 hour, followed by incubation with a DNAJC6 antibody (1:50 dilution, Proteintech; 21941-1-AP) for 48 hours. After three PBS washes, sections were incubated with the ImmPRESS-AP anti-rabbit IgG (alkaline phosphatase) polymer detection reagent (Vector Laboratories, MP-5402) for 30 minutes at room temperature. DNAJC6 signals were visualized using the Vector Red alkaline phosphatase substrate kit (Vector Laboratories). The slides were then washed three times with PBS and processed using the Vector ABC Kit (Vector Laboratories, Burlingame, CA, USA). To assess DNAJC6 expression in astrocytes, sequential immunostaining for GFAP and DNAJC6 was performed. Sections were first blocked with 2.5% normal horse serum (Vector Laboratories) for 1 hour and incubated with a GFAP antibody(1:200 dilution, AB554) for 24 hours. After three PBS washes, the GFAP immunoreactive signals were developed using the Vector ABC Kit and DAB chromogen (Thermo Fisher Scientific, Meridian, Rockford, IL, USA). Following GFAP staining, DNAJC6 immunostaining was performed on the same sections using the protocol described above. Double-stained tissue slides were then sequentially dehydrated through an ethanol series (60%, 80%, 90%, and 100%, each for 5 minutes), immersed in xylene baths (two times, 5 minutes each), and mounted for imaging.

References

1. Wulansari N, Darsono WHW, Woo HJ, Chang MY, Kim J, Bae EJ, et al. Neurodevelopmental defects and neurodegenerative phenotypes in human brain organoids carrying Parkinson's disease-linked DNAJC6 mutations. *Sci Adv.* 2021;7(8).
2. Sun N, Malide D, Liu J, Rovira, II, Combs CA, and Finkel T. A fluorescence-based imaging method to measure in vitro and in vivo mitophagy using mt-Keima. *Nature protocols.* 2017;12(8):1576-87.
3. Bryant JD, Lei Y, VanPortfliet JJ, Winters AD, and West AP. Assessing Mitochondrial DNA Release into the Cytosol and Subsequent Activation of Innate Immune-related Pathways in Mammalian Cells. *Curr Protoc.* 2022;2(2):e372.
4. Hou F, Sun L, Zheng H, Skaug B, Jiang QX, and Chen ZJ. MAVS forms functional prion-like aggregates to activate and propagate antiviral innate immune response. *Cell.* 2011;146(3):448-61.

Supplementary Tables

Table S1. Human ESC and Human iPSC used in this study

Name	Cell Lines Identifier	Mutation	Establishing Institute
H9 hESC	WA09	-	WiCell
WT iPSC	-	-	Dr. Mark Cookson's Lab (National Institute on Aging)
LRRK2 G2019S PL2A9	NIAi001-C	G2019S homozygous mutation in LRRK2 gene	Dr. Mark Cookson's Lab (National Institute on Aging)
LRRK2 R1441C PL2F5	NIAi001-G	R144C homozygous mutation in LRRK2 gene	Dr. Mark Cookson's Lab (National Institute on Aging)
LRRK2 N1437H PL3D7	NIAi001-I	N1437H homozygous mutation in LRRK2 gene	Dr. Mark Cookson's Lab (National Institute on Aging)
LRRK2 K1960M PL2H7	NIAi001-K	K1960M homozygous mutation in LRRK2 gene	Dr. Mark Cookson's Lab (National Institute on Aging)

Table S2. Virus list used in this study

<table>
<tr><th>Virus type</th><th>Vector</th><th>Promoter</th><th>Target gene</th><th>Note1</th><th>Note2</th></tr>
<tr><td rowspan="8">Lentivirus</td><td>pEF1α- human α-synuclein</td><td>EF1α</td><td>human α-synuclein</td><td></td><td>Conventional overexpression</td></tr>
<tr><td>pEF1α-DNAJC6gRNA1,2</td><td rowspan="3">EF1α</td><td rowspan="3">DNAJC6</td><td>DNAJC6 gRNA1,2</td><td rowspan="3">CRISPR-knockdown</td></tr>
<tr><td>pEF1α-DNAJC6gRNA3,4</td><td>DNAJC6 gRNA3,4</td></tr>
<tr><td>pEF1α-Cas13d (Cas-RX)</td><td>Cas13d (Cas-RX)</td></tr>
<tr><td>pEF1α-DNAJC6gRNA1,2-dCas-VP64</td><td rowspan="2">EF1α</td><td rowspan="2">DNAJC6</td><td>DNAJC6 gRNA1,2 dCas-VP64</td><td rowspan="2">CRISPR-overexpression</td></tr>
<tr><td>pEF1α-DNAJC6gRNA3.4-dCas-VP64</td><td>DNAJC6 gRNA3,4 dCas-VP64</td></tr>
<tr><td>pSynapsin-DNAJC6gRNA1,2-dCas-VP64</td><td rowspan="2">Synapsin</td><td rowspan="2">DNAJC6</td><td>DNAJC 6gRNA1,2 dCas-VP64</td><td rowspan="2">CRISPR-overexpression</td></tr>
<tr><td>pSynapsin-DNAJC6gRNA1,2-dCas-VP64</td><td>DNAJC6 gRNA3,4 dCas-VP64</td></tr>
</table>

<table>
<tr><td>AAV2</td><td>pSynapsin-human α-synuclein</td><td>Synapsin</td><td>human α-synuclein</td><td></td><td>Conventional overexpression</td></tr>
<tr><td rowspan="5">AAV9</td><td>pSynapsin-human α-synuclein</td><td>Synapsin</td><td>human α-synuclein</td><td></td><td>Conventional overexpression</td></tr>
<tr><td>pCMV-DNAJC6gRNA1,2</td><td rowspan="4">CMV</td><td rowspan="4">DNAJC6</td><td>DNAJC6gRNA1,2</td><td rowspan="4">CRISPR-overexpression (CRISPR-SAM)</td></tr>
<tr><td>pCMV-DNAJC6gRNA3,4</td><td>DNAJC6gRNA3,4</td></tr>
<tr><td>pCMV-dCas-VP64</td><td>dCas-VP64</td></tr>
<tr><td>pCMV-MS2-TET1</td><td>MS2-TET1</td></tr>
<tr><td rowspan="4">AAV5</td><td>pgfaABC1D-DNAJC6gRNA1,2</td><td rowspan="4">gfaABC1D</td><td rowspan="4">DNAJC6</td><td>DNAJC6gRNA1,2</td><td rowspan="4">CRISPR-overexpression system (CRISPR-SAM)</td></tr>
<tr><td>pgfaABC1D-DNAJC6gRNA3,4</td><td>DNAJC6gRNA3,4</td></tr>
<tr><td>pgfaABC1D-dCas-VP64</td><td>dCas-VP64</td></tr>
<tr><td>pgfaABC1D-MS2-TET1</td><td>MS2-TET1</td></tr>
</table>

Table S3. Antibody list used in this study

Target	# Catalog	Company	Working conc.
**Primary Antibody for immunostaining**			
DNAJC6	21941-1-AP	proteintech	1:50
NURR1	PP-N1404-0C	R&D system	1:1000
FOXA2	sc-374376	Santa-cruz	1:1000
LAMP1	ab25630	abcam	1:200
TUJ1	801202	biolegend	1:1000
Calnecxin	adi-spa-860-d	Enzo life sciences	1:500
phospho-synuclein (phospho S129)	825701	biolegend	1:20,000
TH	P40101-150	Pel-Freez	1:1000
GFAP	AB5541	Millipore	1:1000
MAP2	M1406	Millipore	1:1000
Iba1	019-19741	Wako	1:500
GBA	ab55080	abcam	1:500
Synapsin1	Ab1543	abcam	1:1000
CD11b	CBL1512	Millipore	1:200
CD68	MAB1435	Millipore	1:200
**Secondary Antibody for immunostaining**			
Alexa Fluor 488 goat anti-rabbit	A11008	Thermo Scientific	1:1000
Alexa Fluor 488 goat anti-mouse	A11001	Thermo Scientific	1:1000

Alexa Fluor 546 donkey anti-rabbit	A10040	Thermo Scientific	1:1000
Alexa Fluor 546 goat anti-mouse	A11003	Thermo Scientific	1:1000
Alexa Fluor 647 goat anti-chicken	A21449	Thermo Scientific	1:1000
**Primary Antibody for western blot**			
DNAJC6	HPA031182	Atlas-antibodies	1:1000
GAPDH	sc-47724	Santa-cruz	1:1000
LRRK2 (phospho S935)	ab133450	Abcam	1:1000
LRRK2	ab181386	Abcam	1:1000
β-actin	MA1-140	invitrogen	1:50,000
LC3b	nb100-2220	novusbio	1:1000
P62	pm045	MBL	1:3000
TBK1	#3504	Cell Signaling	1:1000
Phspho-TBK1	#5483	Cell Signaling	1:1000
IRF3	#4302	Cell Signaling	1:1000
Phospho-IRF3	#29047	Cell Signaling	1:1000
STING	ab92605	abcam	1:1000
cGAS	#31659	Cell Signaling	1:1000
NLRP3	ag-20b-0014-c100	AdipoGen	1:1000
ASC	13833s	Cell Signaling	1:1000
Caspase-1	AG-20B-0042-C100	AdipoGen	1:1000
MAVS	Sc-166583	Santa-cruz	1:1000
**Secondary Antibody for immunostaining**			
Anti-rabbit IgG, HRP	#7074	Cell Signaling	1:1000
Anti-mouse IgG, HRP	#7076	Cell Signaling	1:1000

Table S4. Primer sequences used in this study

Target	Primer (Forward)	Primer (Reverse)
**qPCR Primer for Human Genes**		
GAPDH	TTGAGGTCAATGAAGGGGTC	GAGGTGAAGGTCGGAGTCA
DNAJC6	AGGCATTACGGACAAAGTGG	TTTTGCTGGGCTTCTTGACT
FOXA2	TACGACGACATGTTCATGGAGC	TATGCTGGGAGCGGTGAAGA
GFAP	CAGGAAGCAGATGAAGCC	GACGCCATTGCCTCATAC
IL1b	CAGAAGTACCTGAGCTCGCC	GGTCGGAGATTCGTAGCTGG
INOS	CAATGTGGAGAAAGCCCCCT	GCTGCCCCAGTTTTTGATCC
IL6	GGTCGGAGATTCGTAGCTGG	ACATGTCTCCTTTCTCAGGGC
TNFa	GGCGTGGAGCTGAGAGATAA	GCTCTTGATGGCAGAGAGGA
C3	AAGTCCTCGTTGTCCGTTCC	TGCGAACAGCCACAGTTTTG

CXCL10	CTGCCATTCTGATTTGCTGCC	ACACGTGGACAAAATTGGCTT
SERPING1	TCTCCTAACACTACCCCGCA	GCTGCTGCACTTCAAAGACC
ARG1	TTCACACCAGCTACTGGCAC	CGAGCAAGTCCGAAACAAGC
IL10	AAGACCCAGACATCAAGGCG	ACTCATGGCTTTGTAGATGCCT
BDNF	TGAGGACCAGAAAGTTCGGC	CGCCGTTACCCACTCACTAA
GDNF	GAAGTTATGGGATGTCGTGGCT	TCCTCTGGCATATTTGAGTCACTG
CLCF1	GACTCGTGGGGGATGTTAGC	AGTCTGGCTCGTTGAAAGGG
S100A1	TTCGCTGGGGATAAAGGCTAC	AGCTCTGGAAGCCCACTTTG
CD109	GAACACTGCCCTTCACAGGT	CTGGGTACGTCCGGTTACAC
LCN2	GACCCGCAAAAGATGTATGCC	GCTGGTTGTAGTTGGTGCTC
STEAP4	AAGTCGGCAGGTGTTTGTGT	ATTGGAAATAGCTGCAGGGGG
OSMR	TGTAAGCAAGCCAGAAGGGAC	GCATACTTGGGGCTTTTGGG
PSMB8	GCCTTTAGATGACACGACCCT	TCAATCACCTTGTTCACCCGT
AMIGO2	CTTCCATGCCAATGCACCAC	CCACAGGCGACAGGTGTAAT
PTX3	TGCCTGCATTTGGGTCAAAG	GCCGCCAGTTCACCATTTAC
TMEM119	TTCTTCCGCCAGTACGTGAT	TCTGTCGGGGACCTCACTG
**qPCR Primer for Mouse Genes**		
Gapdh	AACTTTGGCATTGTGGAAGG	ACACATTGGGGGTAGGAACA
Dnajc6	CAGGCAGGCTCCAAGTCTAC	CAGTCCAATTCCTGGTCGCT
**Primer for ChIP-qPCR**		
NURR1/FOXA2 on *DNAJC6* (I)	AGTCGAAGCAGTGAGCAAGG	TCTCCCACTCTCCTTCCCTG
NURR1/FOXA2 on *DNAJC6* (II)	CGAGCAGTGATTGTGGGAGT	GGACATTCTTTGGCTTGCCC
NURR1/FOXA2 on *DNAJC6* (III)	AACCAGATGTACAAATAAGTGGGC	GTCCATGGGAAAAGAATGTGGTC
NURR1/FOXA2 on *DNAJC6* (IV)	CTTAGCTGGGAAGTCTAGGTCC	ATGCATGTCATTGTGTTTACAAAGC
NURR1/FOXA2 on *DNAJC6* (V)	CTGTAAAAGAGGACGGGCAACA	GGTATATGCATGTGTGTGGCTAAA
NURR1/FOXA2 on *DNAJC6* (VI)	AATGAGCCCAAGGTACGTGAAT	AGGCTGCACTCTCATTTTGCTA
**Primer for mtDNA Copy Number**		
KCJN10	GCGCAAAAGCCTCCTCATT	CCTTCCTTGGTTTGGTGGG
D-LOOP	CATAAAGCCTAAATAGCCCACACG	CCGTGAGTGGTTAATAGGGTGATA

Table S5. Postmortem brain tissue used in this study

Phenotype	Age	Sex	Lewy body	Motor symptom	Non-motor symptom	Pin#
Normal #1	83	Female	No	-	-	20637
Normal #2	83	Female	No	-	-	20685
Normal #3	69	Male	No	-	-	10640
PD #1	87	Male	Yes-diffuse/neocortex	Yes	Not available	21220
PD #2	88	Feale	Yes-diffuse/neocortex	Yes	Not available	20838
PD #3	82	Male	Yes-limbic/transitional	Yes	Not available	20848

Supplementary Figure legends

Suppl. Fig. S1. Gene Ontology (GO) and KEGG pathway analyses of differentially expressed genes (DEGs) in the datasets shown in Fig. 1C.

(A) GO analysis of DEGs from dataset GSE68719, the primary dataset used in this study.

(B) Upregulated and downregulated KEGG pathways identified in additional PD-associated transcriptome datasets (GSE49036, GSE208783, ERP129142, and GSE207713).

Suppl. Fig. S2. Expression patterns of chaperone genes in the PD-associated datasets shown in Fig. 1C.

(A) Expression levels of the selected chaperones DNAJC6, GAK, SYNJ1, and BAG3 in PD conditions compared with WT controls across the transcriptome datasets GSE49036, GSE208783, ERP129142 (GBA), ERP129142 (SNCA), and GSE207713.

(B) Scatterplots showing additional chaperone genes that are significantly upregulated or downregulated in PD compared with WT controls ($p<0.05$).

Upregulated and downregulated values (A) and genes (B) in PD are highlighted with green and yellow boxes, respectively.

Suppl. Fig. S3. Protocol summary for generating human brain cell cultures from human pluripotent stem cells (hPSCs).

(A, B) Generation of midbrain cultures enriched with midbrain-type dopamine (mDA) neurons and astrocytes (mAST). hPSCs were differentiated into midbrain-like organoids in 3D culture. Neural stem/precursor cells (NSCs) were isolated from these organoids and expanded in 2D culture using NSC proliferation media across multiple passages. Early-passage NSCs (Passage 1-3, P1–3) predominantly differentiated into neurons, including mDA neurons, while late-passage NSCs (P8–10) primarily produced mAST. Midbrain cultures enriched for neurons, mixed neurons/astrocytes, or astrocytes were prepared using NSCs from passages P1–3, P4–6, and P8–10, respectively. The regional and cell-type identities of the cultures were validated by immunocytochemistry for midbrain DA neuronal markers (e.g. NURR1, FOXA2, EN1, TH), and astrocyte-specific markers (e.g., GFAP, S100β, AQP4).

(C–E) Derivation of microglia-like cells from hPSC-derived yolk sac (hematopoietic) organoids.
(C) Representative images of Iba1+, TMEM119+, and CX3CR1+ microglia-like cells.
(D) Confirmation of microglial phenotypic identity through qPCR analysis of microglia-specific markers.
(E) Functional validation of microglia-like cells by demonstrating upregulation of pro-inflammatory genes in response to stimuli such as LPS and IL-4.

Suppl. Fig. S4. Quantification of the numbers of DA neurons (A), synapses (B), and NURR1 and FOXA2 expression (C) in the toxin-treated cultures used in Fig. 1G.
NURR1 and FOXA2 expression in (C) was assessed by mean fluorescence intensity (MFI) using ImageJ. * $p > 0.05$, one-way ANOVA followed by dunnett's test.
n = 6-9 cultures for (A); 60-120 TH^{+} neurites (from 6-9 cultures) for (B); 40-90 cells (from 6-9 cultures) for (C).

Suppl. Fig. S5. Rank-ordered data of Nurr1- and Foxa2-induced gene expression changes in primary mouse NSC (mNSC) and glial (mGlia) cultures (microarray datasets GSE54086 and GSE145489).

Suppl. Fig. S6. Treatment with α-syn PFF in hPSC-derived human midbrain cultures led to downregulation of the midbrain-specific transcription factors NURR1 and FOXA2 (A-B). Expression was assessed by the percentage of immunoreactive cells among total (DAPI+) cells (A) or by the mean fluorescence intensity (MFI) of each cell (B). (C) Immunoblot analysis showing the recovery of α-syn PFF treatment-induced DNAJC6 protein loss upon forced expression of NURR1 and FOXA2 in hESC-derived midbrain cultures. *$p<0.05$, **$p<0.01$, unpaired t-test (A) or one-way ANOVA followed by Tukey's post hoc analysis (B). NS, Not Significant.

Suppl. Fig. S7. Co-IP assay showing that the LRRK2 K1906M variant, a non-pathogenic kinase-dead form, does not induce serine phosphorylation of DNAJC6 proteins. This

experiment serves as a negative control for the WT LRRK2-mediated serine phosphorylation of DNAJC6 shown in Fig. 2K.

Suppl. Fig. S8. The expression level of DNAJC6 was analyzed in human neurons, astrocytes and microglia cultures derived from hESCs. The expression levels in each cell type were determined using immunocytochemical (A) and qPCR analyses (B). *$p<0.05$, **$p<0.01$, ***$p<0.0001$; n= 32 cells (A) and 3 independent experiments (B) NS, Not Significant.; One-Way ANOVA followed by Dunn's test (A) or Tukey's test (B).

Suppl. Fig. S9. (A) Scatterplot analysis of differentially expressed genes (DEGs) in astrocytes derived from isogenic LRRK2G2019S-hiPSCs compared to WT hiPSC-astrocytes (GSE207713). The arrow indicates the position of DNAJC6 among the downregulated DEGs. (B) Gene ontology (GO) analysis of downregulated DEGs in LRRK2G2019S astrocytes compared to WT astrocytes.

Supple Fig. S10. PINK1 and Parkin levels in the mitochondrial fractions analyzed by WB. PINK1 and Parkin levels in the mitochondrial fractions analyzed by immunoblot (left blots) and the protein levels were analyzed (right).

Suppl. Fig. S11. Determination of mitochondrial DNA (mtDNA) levels and MAVS activation
(A) Detection of cytosolic mitochondrial DNA (mtDNA).
(B, C) MAVS activation assessed through Western blot for aggregated MAVS protein levels (B) and immunocytochemistry (C). *$p<0.05$, NS, Not Significant, n=3~4.

Supple Fig. S12. The *in vitro* human PD tri-culture model used in this study. (A) Schematic overview of the procedure for generating the in vitro human PD tri-culture model. (B) Pathological misfolded α-syn aggregation by α-syn-Westen Blot (WB) using Triton X-100-soluble and insoluble fractions.

(C, D) Misfolded α-syn aggregation propensity measured by the percentage of Thioflavin S-stained cells (C) and % pS129-α-syn+/TH+ cells (D).
(E) Synaptic degeneration assessed by the number of abnormal synaptic vesicle clusters (>5μm synapsin+ clumps).
(F) Astrocyte viability assessed by % GFAP+ cells of DAPI+ cells
*$p<0.05$, **$p<0.001$; Paired t-test (B), Unpaired t-test (C).

Suppl. Fig. S13. Astrocytic DNAJC6 knockdown (KD) effects in mDA neuron–astrocyte dual co-cultures (lacking microglia). (A) WB analysis for detecting pathological a-syn aggregates. (B) % Thioflavin S-stained cells. (C) % pS129-α-syn+/TH+ cells. (D) Synaptic degeneration assessed by the number of abnormal synaptic vesicle clusters (>5μm synapsin+ clumps). *$p<0.05$; Paired t-test (A), Unpaired t-test (B-D).

Suppl. Fig. S14. Astrocytic DNAJC6-KD effects on microglia. (A, B) DNAJC6-KD astrocytes (or control astrocytes) were co-cultured with human microglia in the presence of a-syn PFF. Pro-inflammatory and senescence phenotype of the microglia were assessed by % Iba1+ microglia expressing IL1β and P21(A) and mRNA expression levels of pro-inflammatory cytokines (B). (C) Medium was conditioned in the cultures for DNAJC6-KD astrocytes (or control astrocytes) and the conditioned media (CM) were treated to a-syn PFF-treated microglia cultures. The percentage of Iba1+ microglia expressing IL1β and P21 were assessed. *$p<0.05$; Unpaired t-test.

Suppl. Fig. S15. Comparison of DNAJC6 expression levels and cell toxicity induced by conventional universal promoter-driven and CRISPRa-mediated lentiviral transduction.
(A) Schematic representation of the vector constructs.
(B) DNAJC6 exogenous expression levels quantified by qPCR in human neuron- and astrocyte-enriched cultures derived from hESCs.
(C) Cellular toxicity comparison, measured as the percentage of EtHD+ dead cells.
*$p<0.05$, **$p<0.01$, ***$p<0.0001$, NS, Not Significant; n=3, Unpaired t-test

Suppl. Fig. S16. Therapeutic effects of neuron-specific CRISPRa-DNAJC6 on α-syn-PD pathologies in an *in vitro* model.

(A) Schematic representation of the experimental procedure (B).

(B–E) Pathological misfolded α-syn aggregation analyzed by α-syn Western blot (WB) using Triton X-100-soluble and insoluble fractions (B) and immunocytochemistry (ICC) for the percentage of Thioflavin S-stained cells (C) and pS129-α-syn+/TH+ cells (D) and synaptic vesicle clustering, quantified as the number of synapsin I+ clumps >5 μm (E). $*p < 0.05$, $**p < 0.001$; paired t-test (B) or unpaired t-test (C–E).

Suppl. Fig. S17. Effects of DNAJC6 overexpression in both neuronal and astrocytic cells on α-syn aggregation and synaptic degeneration in DA neurons, compared with overexpression in either cell type alone.

(A) Percentage of Thioflavin S–positive cells.

(B) Percentage of pS129–α-syn^+/TH^+ cells.

(C) Synaptic vesicle clustering, quantified as the number of synapsin I^+ clumps >5 μm.

$*p<0.05$ compared to PD-control, Two-way ANOVA followed by Tukey's analysis.

Suppl. Fig. S18. Morphometric analysis of astrocytes (A) and microglia (B) in the midbrain SNs of WT and α-syn PD mice with CRISPRa-mediated DNAJC6 overexpression.

Representative images of $GFAP^+$ astrocytes (A) and $Iba1^+$ microglia (B) are shown alongside quantitative assessments of morphometric parameters.

A total of 40-70 astrocytes (A) and 30-60 microglia (B) were analyzed from 3-5 mice.

$*p<0.05$, $****p<0.0001$, One-way ANOVA followed by Bonferroni post hoc analysis.

Suppl. Fig. S19. CRISPRa-mediated overexpression of DNAJC6 in WT mice did not affect behavior (A) or SN histology (B), supporting the safety of this approach.

Suppl. Fig. S20. Expression levels of chaperone genes in in vitro and in vivo α-syn-PD models with or without DNAJC6 rescue.

qPCR analysis was performed to assess the expression of various chaperones in α-syn-treated hPSC-derived midbrain neuron–astrocyte cocultures (A) and in α-syn-treated mouse midbrain

tissues (B), representing the in vitro and in vivo PD models, respectively. The effects of CRISPRa-mediated DNAJC6 rescue on chaperone expression were also evaluated.

All data were not significantly different among WT, PD, and PD+CRISPRa, One-way ANOVA followed by Bonferroni post hoc analysis.

Figure S1.

A

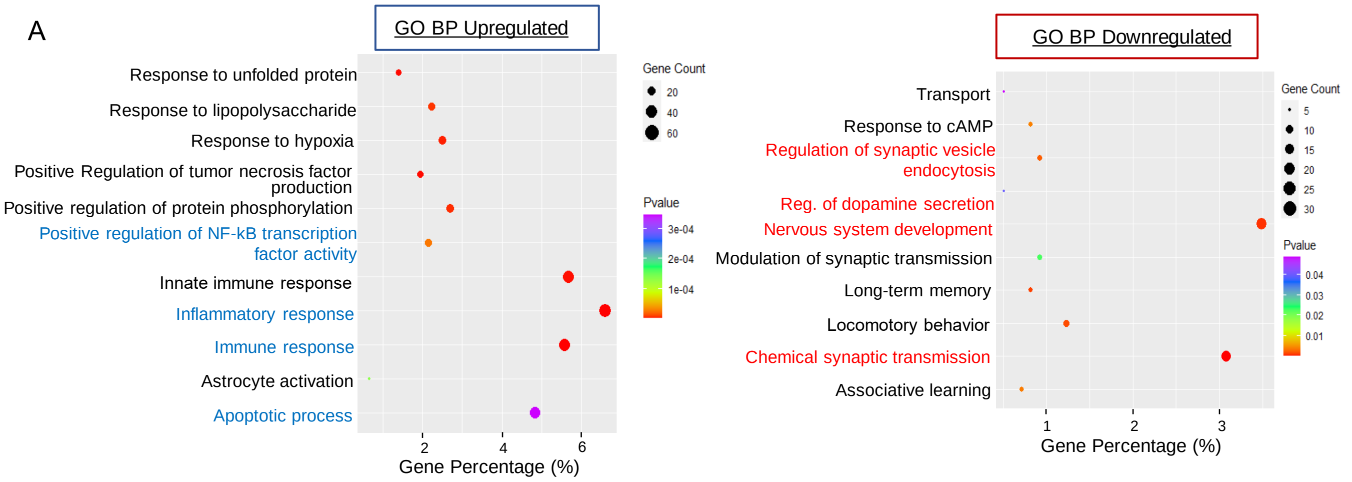

GO BP Upregulated
Response to unfolded protein
Response to lipopolysaccharide
Response to hypoxia
Positive Regulation of tumor necrosis factor production
Positive regulation of protein phosphorylation
Positive regulation of NF-kB transcription factor activity
Innate immune response
Inflammatory response
Immune response
Astrocyte activation
Apoptotic process
Gene Percentage (%)
Gene Count
Pvalue
GO BP Downregulated
Transport
Response to cAMP
Regulation of synaptic vesicle endocytosis
Reg. of dopamine secretion
Nervous system development
Modulation of synaptic transmission
Long-term memory
Locomotory behavior
Chemical synaptic transmission
Associative learning
Gene Percentage (%)
Gene Count
Pvalue

B

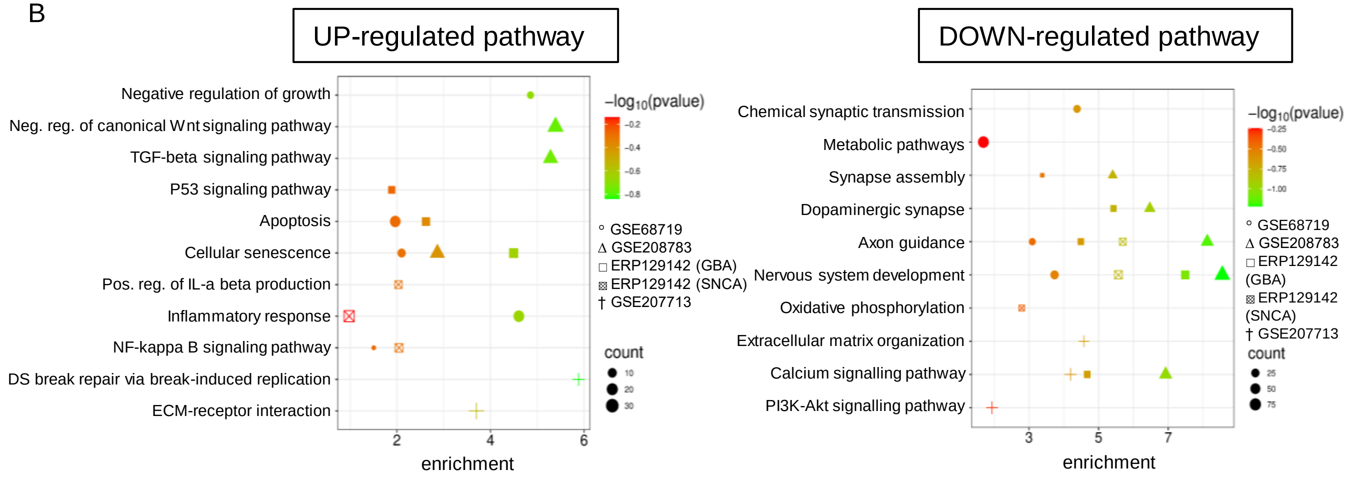

UP-regulated pathway
Negative regulation of growth
Neg. reg. of canonical Wnt signaling pathway
TGF-beta signaling pathway
P53 signaling pathway
Apoptosis
Cellular senescence
Pos. reg. of IL-a beta production
Inflammatory response
NF-kappa B signaling pathway
DS break repair via break-induced replication
ECM-receptor interaction
enrichment
−log10(pvalue)
° GSE68719
Δ GSE208783
□ ERP129142 (GBA)
⊠ ERP129142 (SNCA)
† GSE207713
count
DOWN-regulated pathway
Chemical synaptic transmission
Metabolic pathways
Synapse assembly
Dopaminergic synapse
Axon guidance
Nervous system development
Oxidative phosphorylation
Extracellular matrix organization
Calcium signalling pathway
PI3K-Akt signalling pathway
enrichment
−log10(pvalue)
° GSE68719
Δ GSE208783
□ ERP129142 (GBA)
⊠ ERP129142 (SNCA)
† GSE207713
count

Figure S2.

A

Upregulated
Down-regulated

DNAJC6	log2FC	p value	GAK	log2FC	p value	SYNJ1	log2FC	p value	BAG3	log2FC	p value
GSE68719	-0.35	0.002	GSE68719	0.214794	0.005435	GSE68719	-0.211579222	0.063291	GSE68719	1.02947	6.13E-07
GSE208783	-2.138319482	4.76E-12	GSE208783	0.658784	1.79E-05	GSE208783	-0.849841168	4.15E-09	GSE208783	0.280243	0.017912
ERP GBA	-0.4	0.001778	ERP GBA	0.1	0.708612	ERP GBA	-0.1	0.575821	ERP GBA	0.2	0.538202
ERP SNCA	-0.3	1.15E-06	ERP SNCA	1.1	1.47E-23	ERP SNCA	-0.5	3.42E-10	ERP SNCA	-0.3	0.063122
GSE207713	-0.79814742	0.023461	GSE207713	0.060424	0.530486	GSE207713	0.086985892	0.774664	GSE207713	-0.48985	0.084142

B

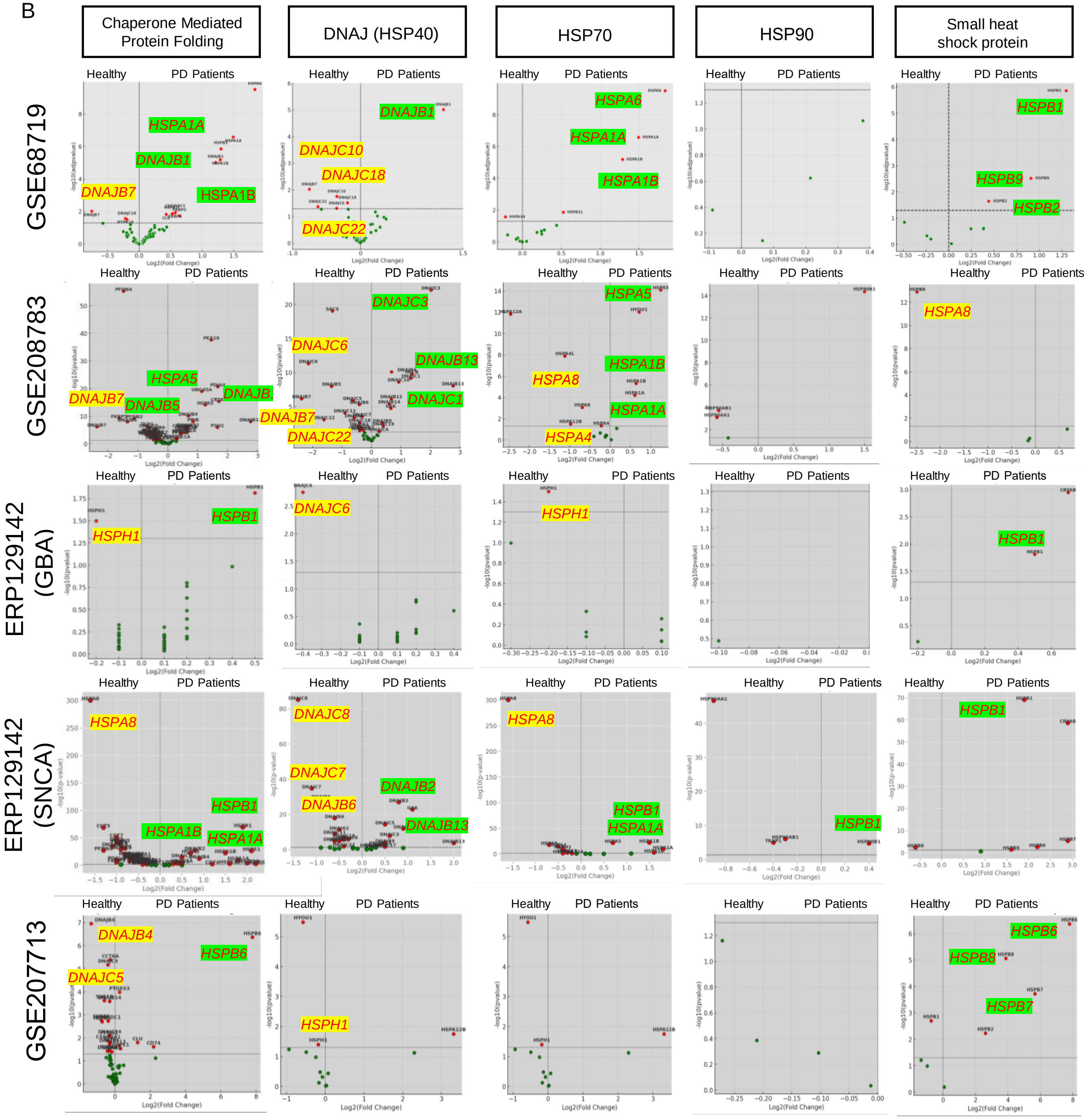

Figure S3.

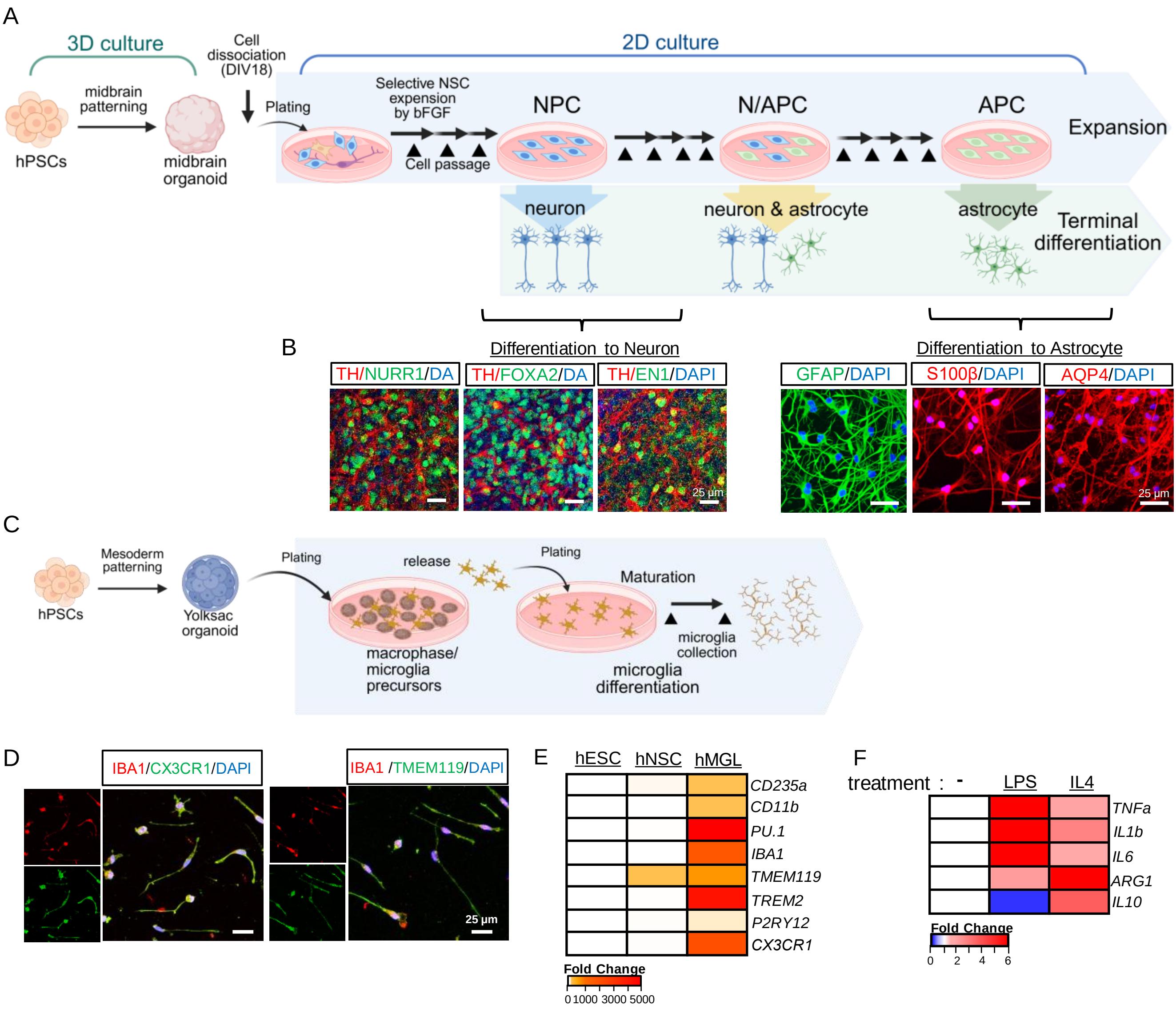

A
3D culture
Cell dissociation (DIV18)
2D culture
hPSCs
midbrain patterning
midbrain organoid
Plating
Selective NSC expension by bFGF
Cell passage
NPC
N/APC
APC
Expansion
neuron
neuron & astrocyte
astrocyte
Terminal differentiation
B
Differentiation to Neuron
TH/NURR1/DA
TH/FOXA2/DA
TH/EN1/DAPI
25 μm
Differentiation to Astrocyte
GFAP/DAPI
S100β/DAPI
AQP4/DAPI
25 μm
C
hPSCs
Mesoderm patterning
Yolksac organoid
Plating
release
Plating
Maturation
macrophage/ microglia precursors
microglia collection
microglia differentiation
D
IBA1/CX3CR1/DAPI
IBA1 /TMEM119/DAPI
25 μm
E
hESC
hNSC
hMGL
CD235a
CD11b
PU.1
IBA1
TMEM119
TREM2
P2RY12
CX3CR1
Fold Change
0 1000 3000 5000
F
treatment : -
LPS
IL4
TNFa
IL1b
IL6
ARG1
IL10
Fold Change
0 2 4 6

Figure S4.

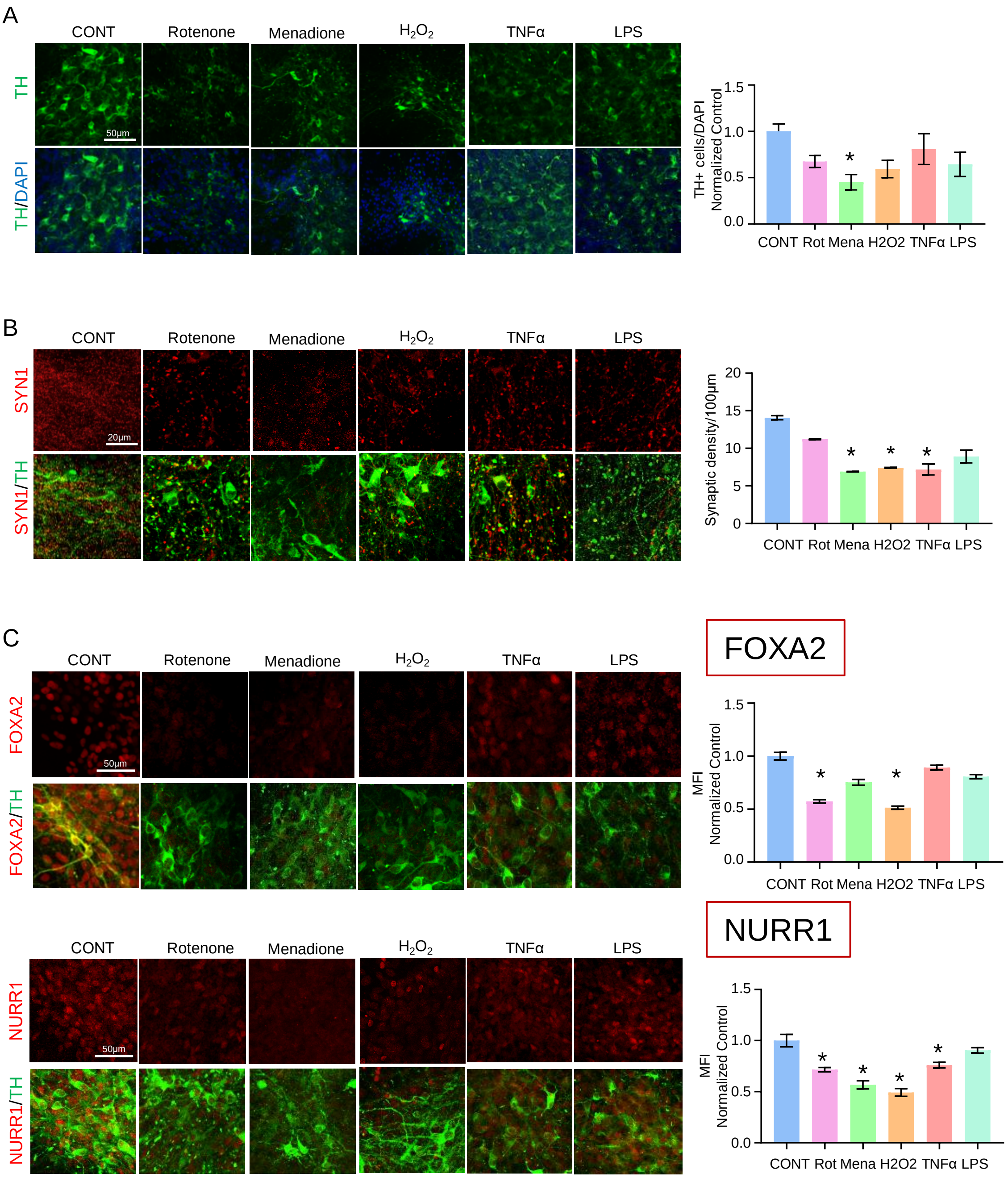

A
CONT
Rotenone
Menadione
H_2O_2
TNFα
LPS
TH
TH/DAPI
50µm
TH+ cells/DAPI
Normalized Control
1.5
1.0
0.5
0.0
*
CONT Rot Mena H2O2 TNFα LPS
B
CONT
Rotenone
Menadione
H_2O_2
TNFα
LPS
SYN1
SYN1/TH
20µm
Synaptic density/100µm
20
15
10
5
0
*
*
*
CONT Rot Mena H2O2 TNFα LPS
C
CONT
Rotenone
Menadione
H_2O_2
TNFα
LPS
FOXA2
FOXA2/TH
50µm
FOXA2
MFI
Normalized Control
1.5
1.0
0.5
0.0
*
*
CONT Rot Mena H2O2 TNFα LPS
CONT
Rotenone
Menadione
H_2O_2
TNFα
LPS
NURR1
NURR1/TH
50µm
NURR1
MFI
Normalized Control
1.5
1.0
0.5
0.0
*
*
*
*
CONT Rot Mena H2O2 TNFα LPS

Figure S5.

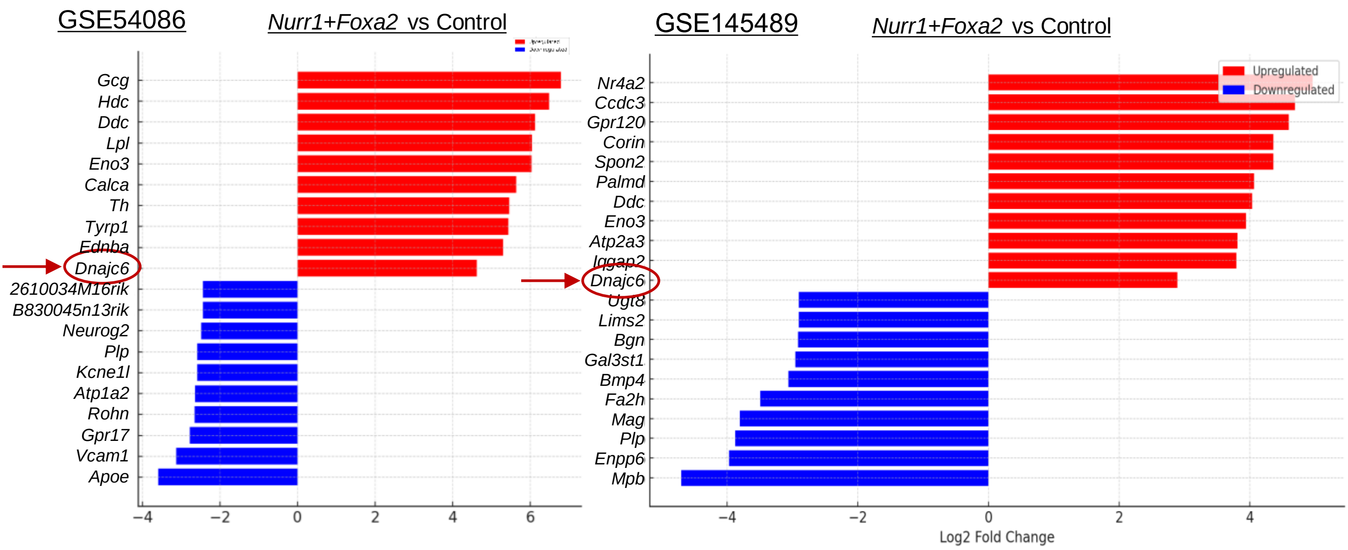
GSE54086
Nurr1+Foxa2 vs Control
Gcg
Hdc
Ddc
Lpl
Eno3
Calca
Th
Tyrp1
Ednba
Dnajc6
2610034M16rik
B830045n13rik
Neurog2
Plp
Kcne1l
Atp1a2
Rohn
Gpr17
Vcam1
Apoe
−4
−2
0
2
4
6
GSE145489
Nurr1+Foxa2 vs Control
Upregulated
Downregulated
Nr4a2
Ccdc3
Gpr120
Corin
Spon2
Palmd
Ddc
Eno3
Atp2a3
Iqgap2
Dnajc6
Ugt8
Lims2
Bgn
Gal3st1
Bmp4
Fa2h
Mag
Plp
Enpp6
Mpb
−4
−2
0
2
4
Log2 Fold Change

Figure S6.

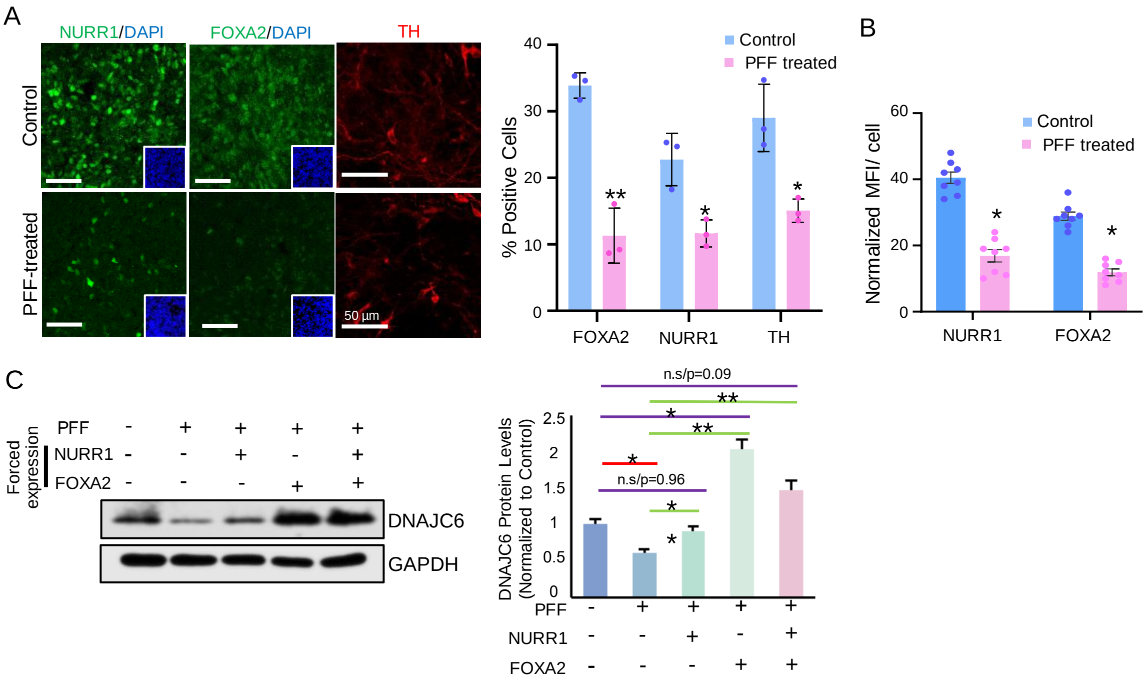
A
NURR1/DAPI
FOXA2/DAPI
TH
Control
PFF-treated
50 µm
% Positive Cells
Control
PFF treated
FOXA2
NURR1
TH
B
Normalized MFI/ cell
Control
PFF treated
NURR1
FOXA2
C
Forced expression
PFF
NURR1
FOXA2
DNAJC6
GAPDH
DNAJC6 Protein Levels (Normalized to Control)
n.s/p=0.09
n.s/p=0.96

Figure S7.

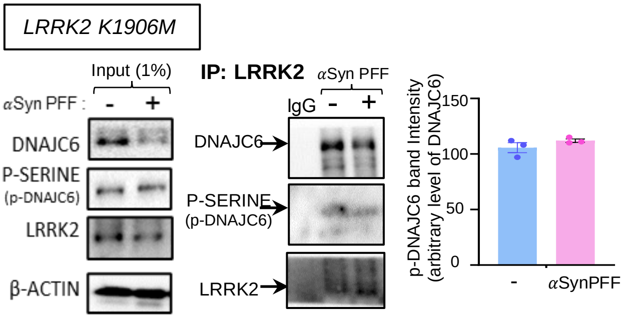
LRRK2 K1906M
Input (1%)
αSyn PFF :
-
+
DNAJC6
P-SERINE
(p-DNAJC6)
LRRK2
β-ACTIN
IP: LRRK2
αSyn PFF
IgG
-
+
DNAJC6
P-SERINE
(p-DNAJC6)
LRRK2
p-DNAJC6 band Intensity
(arbitrary level of DNAJC6)
150
100
50
0
-
αSynPFF

Figure S8

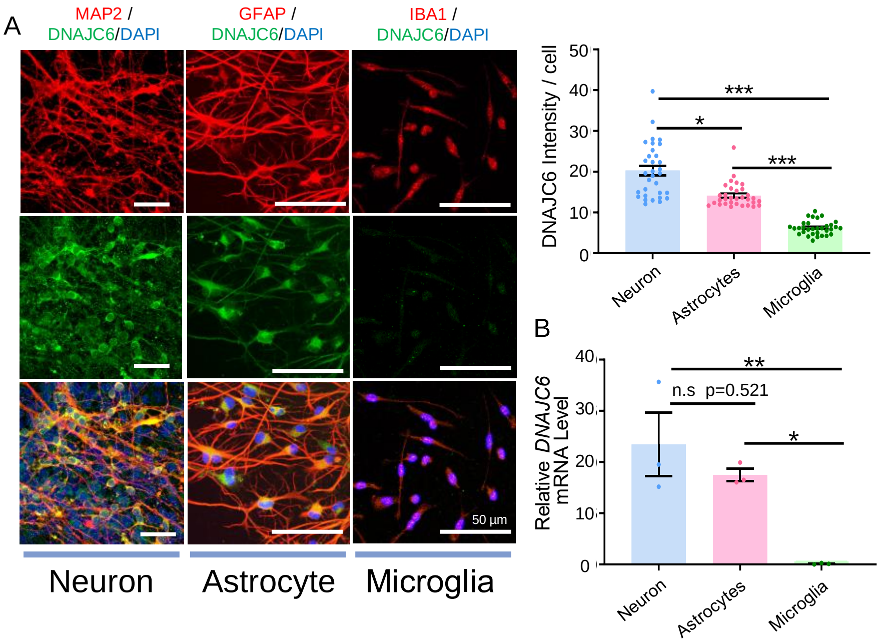

A
MAP2 / DNAJC6/DAPI
GFAP / DNAJC6/DAPI
IBA1 / DNAJC6/DAPI
50 µm
Neuron
Astrocyte
Microglia
DNAJC6 Intensity / cell
50
40
30
20
10
0
***
*
***
Neuron
Astrocytes
Microglia
B
Relative DNAJC6 mRNA Level
40
30
20
10
0
**
n.s p=0.521
*
Neuron
Astrocytes
Microglia

Figure S9.

A

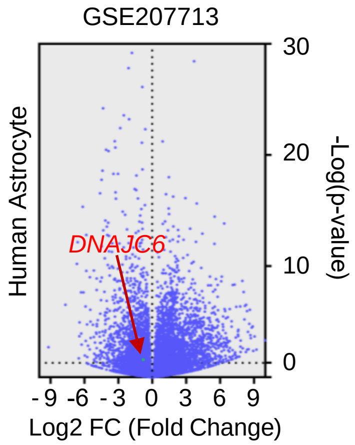
GSE207713
Human Astrocyte
DNAJC6
30
20
10
0
-Log(p-value)
- 9 -6 - 3 0 3 6 9
Log2 FC (Fold Change)

B

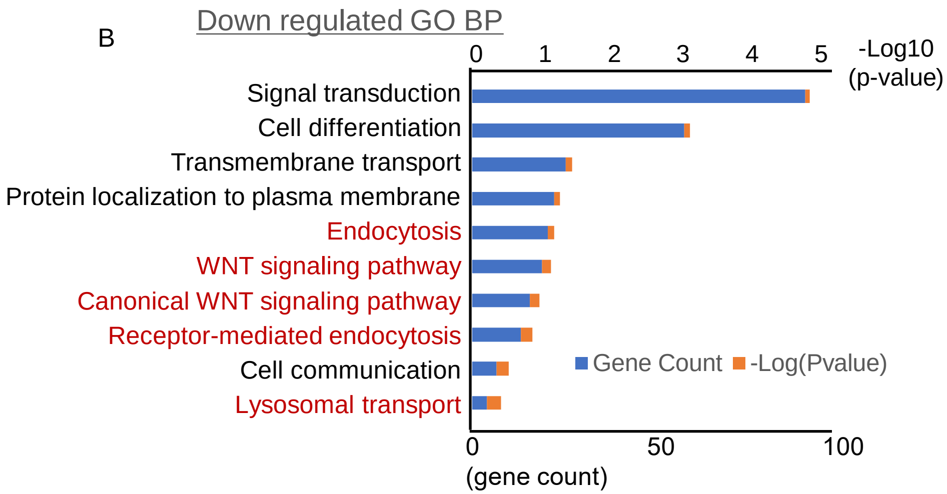
Down regulated GO BP
0 1 2 3 4 5
-Log10 (p-value)
Signal transduction
Cell differentiation
Transmembrane transport
Protein localization to plasma membrane
Endocytosis
WNT signaling pathway
Canonical WNT signaling pathway
Receptor-mediated endocytosis
Cell communication
Lysosomal transport
Gene Count
-Log(Pvalue)
0 50 100
(gene count)

Figure S10.

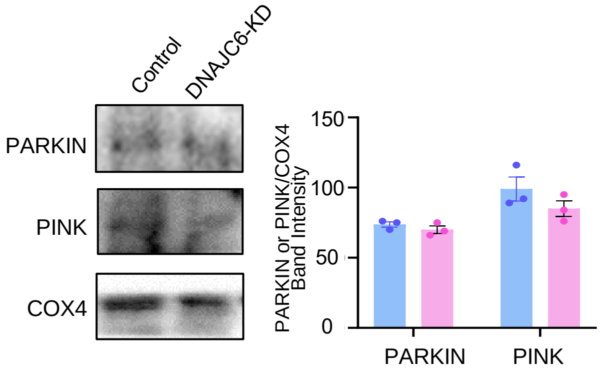

Control
DNAJC6-KD
PARKIN
PINK
COX4
PARKIN or PINK/COX4
Band Intensity
150
100
50
0
PARKIN
PINK

Figure S11.

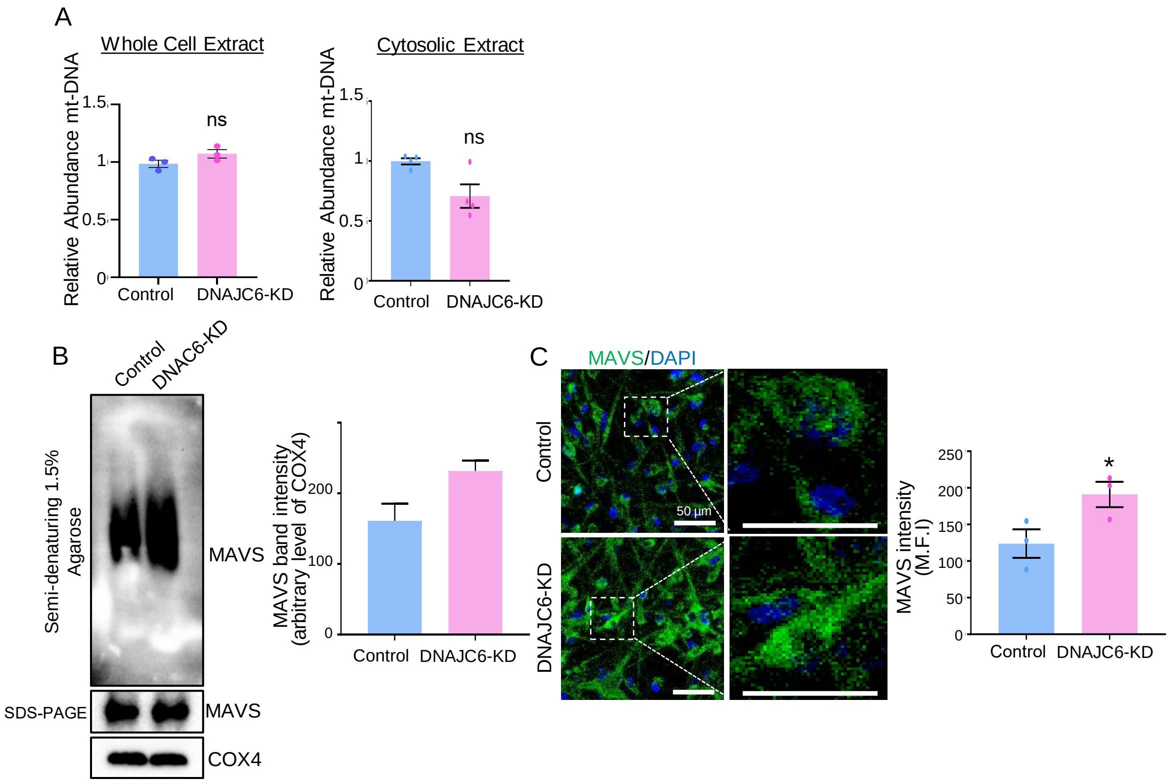
A
Whole Cell Extract
Cytosolic Extract
Relative Abundance mt-DNA
1.5
1
0.5
0
ns
Control
DNAJC6-KD
B
Control
DNAC6-KD
Semi-denaturing 1.5% Agarose
MAVS
SDS-PAGE
MAVS
COX4
MAVS band intensity (arbitrary level of COX4)
200
100
0
C
MAVS/DAPI
50 µm
MAVS intensity (M.F.I)
250
200
150
100
50
*

Figure S12.

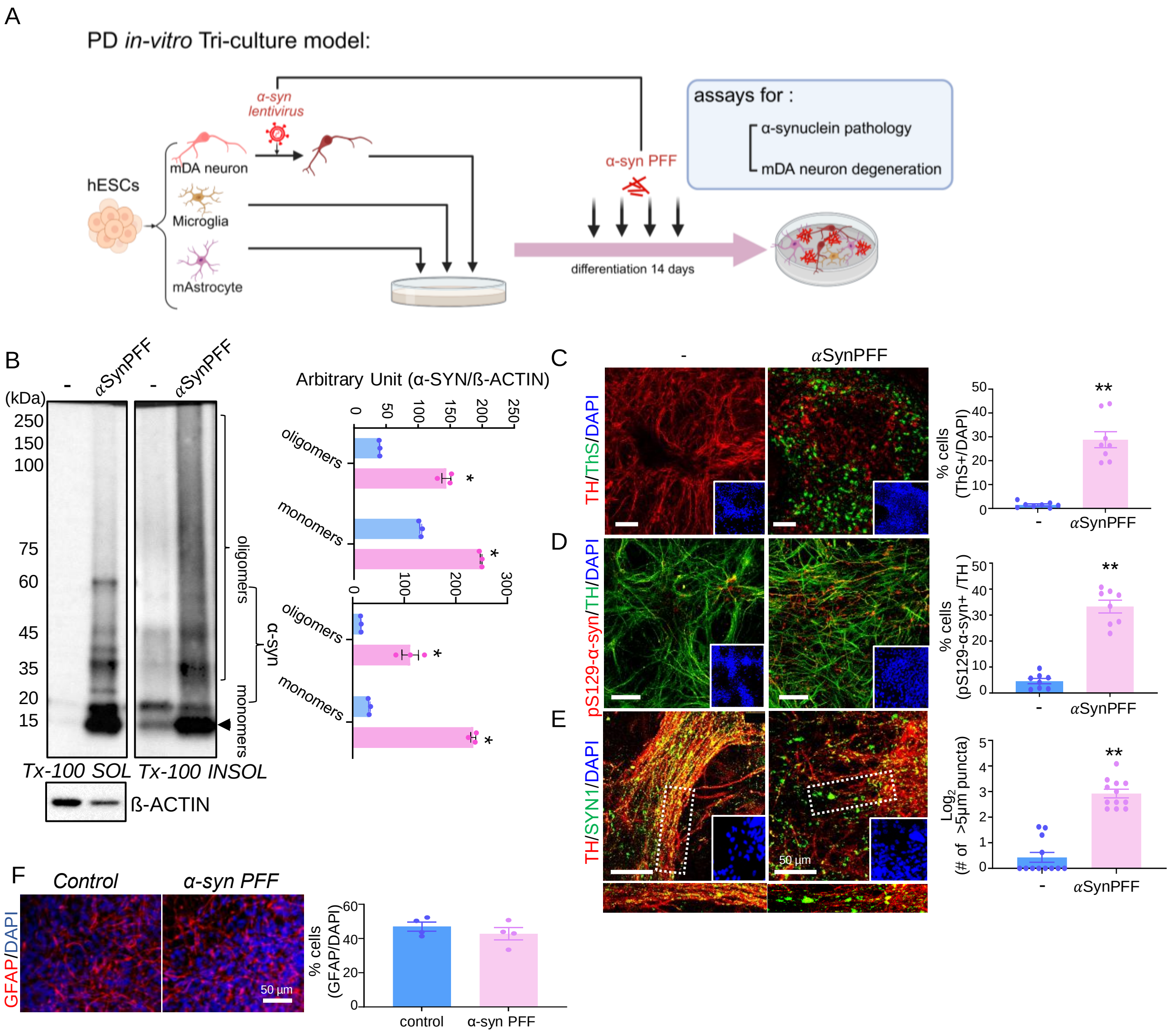
A
PD in-vitro Tri-culture model:
hESCs
mDA neuron
Microglia
mAstrocyte
α-syn lentivirus
α-syn PFF
differentiation 14 days
assays for :
α-synuclein pathology
mDA neuron degeneration
B
αSynPFF
(kDa)
250
150
100
75
60
45
35
20
15
oligomers
monomers
α-syn
Tx-100 SOL
Tx-100 INSOL
ß-ACTIN
Arbitrary Unit (α-SYN/ß-ACTIN)
C
αSynPFF
TH/ThS/DAPI
% cells (ThS+/DAPI)
D
pS129-α-syn/TH/DAPI
% cells (pS129-α-syn+ /TH)
E
TH/SYN1/DAPI
50 µm
Log2 (# of >5µm puncta)
F
Control
α-syn PFF
GFAP/DAPI
50 µm
% cells (GFAP/DAPI)
control
α-syn PFF

Figure S13.

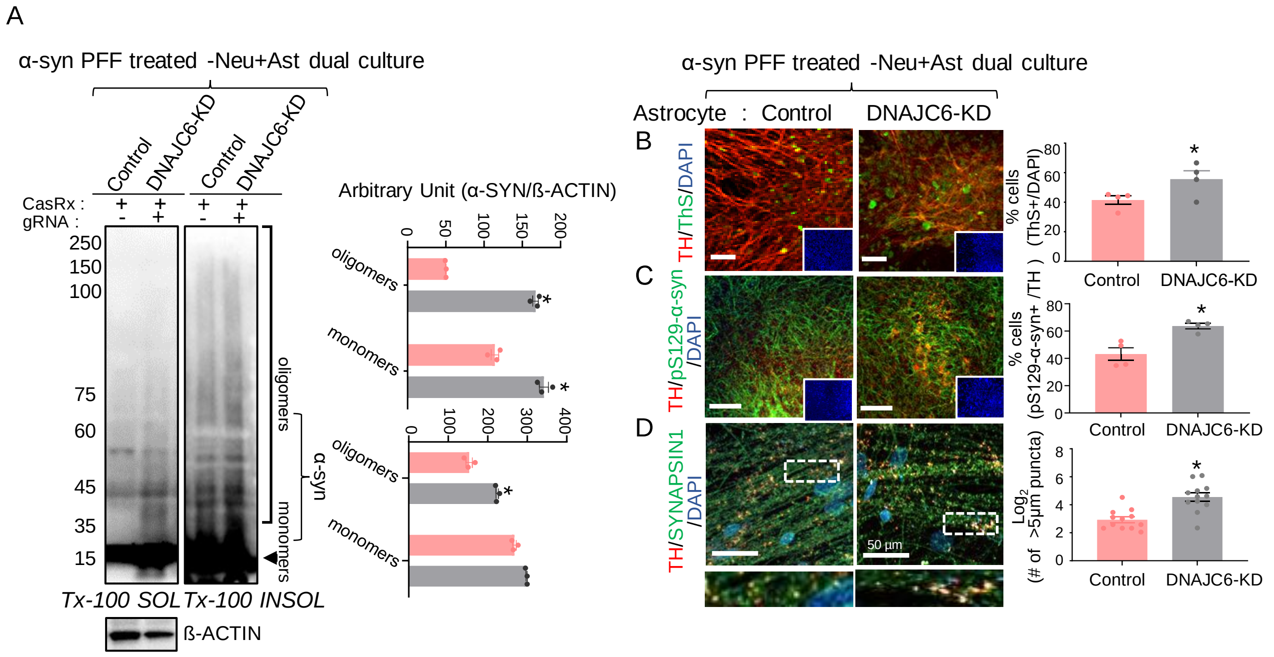
A
α-syn PFF treated -Neu+Ast dual culture
Control
DNAJC6-KD
Control
DNAJC6-KD
CasRx :
gRNA :
250
150
100
75
60
45
35
15
oligomers
monomers
α-syn
Tx-100 SOL
Tx-100 INSOL
ß-ACTIN
Arbitrary Unit (α-SYN/ß-ACTIN)
oligomers
monomers
α-syn PFF treated -Neu+Ast dual culture
Astrocyte : Control DNAJC6-KD
B
C
D
TH/ThS/DAPI
TH/pS129-α-syn/DAPI
TH/SYNAPSIN1/DAPI
50 µm
% cells (ThS+/DAPI)
% cells (pS129-α-syn+ /TH)
Log2 (# of >5µm puncta)
Control
DNAJC6-KD

Figure S14.

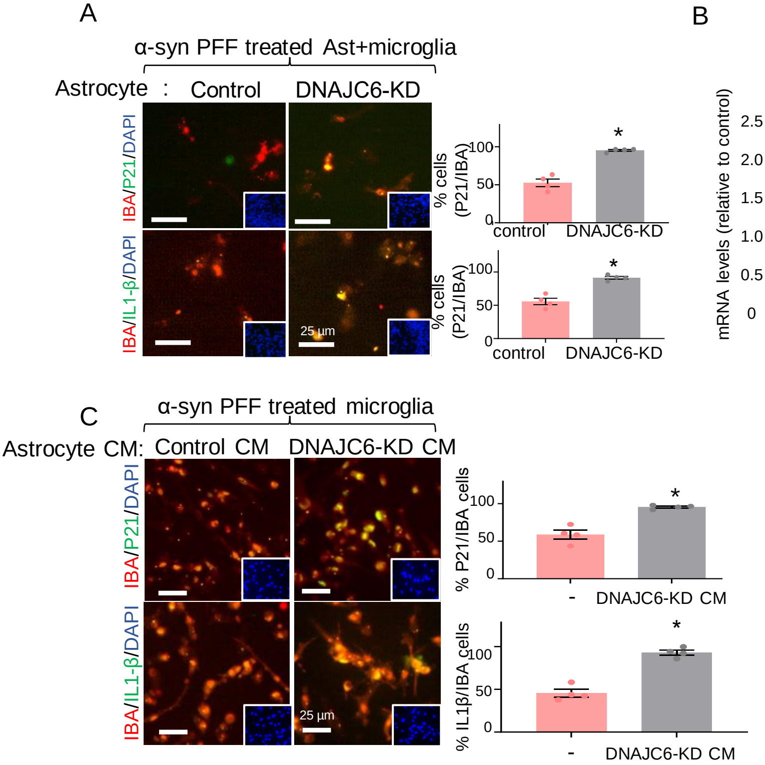

A
α-syn PFF treated Ast+microglia
Astrocyte : Control DNAJC6-KD
IBA/P21/DAPI
IBA/IL1-β/DAPI
25 µm
% cells (P21/IBA)
100
50
0
control DNAJC6-KD
*
B
α-syn PFF treated Ast+microglia
mRNA levels (relative to control)
2.5
2.0
1.5
1.0
0.5
0
Control
DNAJC6-KDx
IL-1β
iNOS
IL-6
TNFα
C
α-syn PFF treated microglia
Astrocyte CM: Control CM DNAJC6-KD CM
% P21/IBA cells
% IL1β/IBA cells
-
DNAJC6-KD CM

Figure S15.

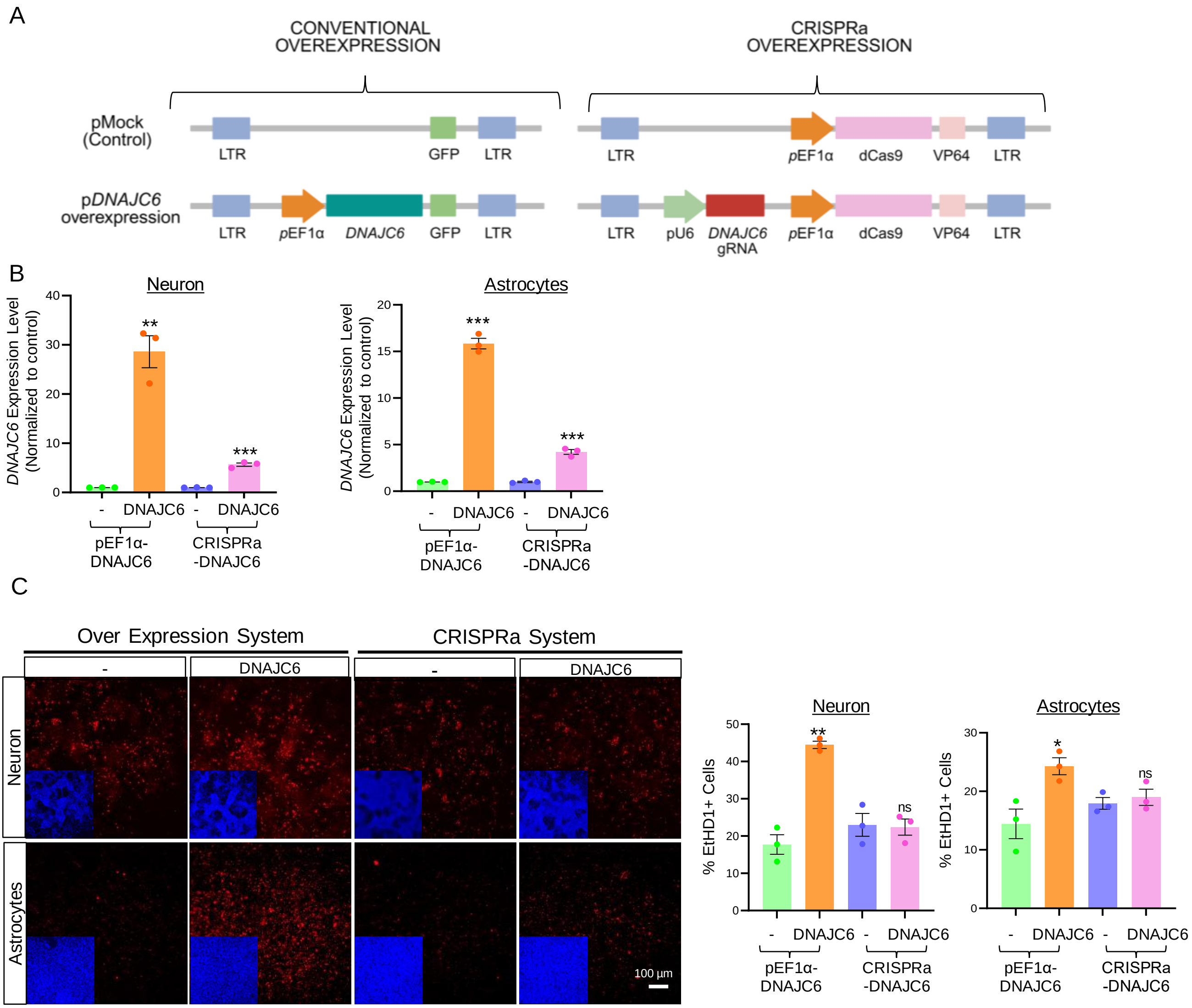
A
CONVENTIONAL OVEREXPRESSION
CRISPRa OVEREXPRESSION
pMock (Control)
LTR
GFP
LTR
LTR
pEF1α
dCas9
VP64
LTR
pDNAJC6 overexpression
LTR
pEF1α
DNAJC6
GFP
LTR
LTR
pU6
DNAJC6 gRNA
pEF1α
dCas9
VP64
LTR
B
Neuron
DNAJC6 Expression Level (Normalized to control)
40
30
20
10
0
**
***
-
DNAJC6
-
DNAJC6
pEF1α-DNAJC6
CRISPRa -DNAJC6
Astrocytes
DNAJC6 Expression Level (Normalized to control)
20
15
10
5
0
***
***
-
DNAJC6
-
DNAJC6
pEF1α-DNAJC6
CRISPRa -DNAJC6
C
Over Expression System
CRISPRa System
-
DNAJC6
-
DNAJC6
Neuron
Astrocytes
100 µm
Neuron
% EtHD1+ Cells
50
40
30
20
10
0
**
ns
-
DNAJC6
-
DNAJC6
pEF1α-DNAJC6
CRISPRa -DNAJC6
Astrocytes
% EtHD1+ Cells
30
20
10
0
*
ns
-
DNAJC6
-
DNAJC6
pEF1α-DNAJC6
CRISPRa -DNAJC6

Figure S16.

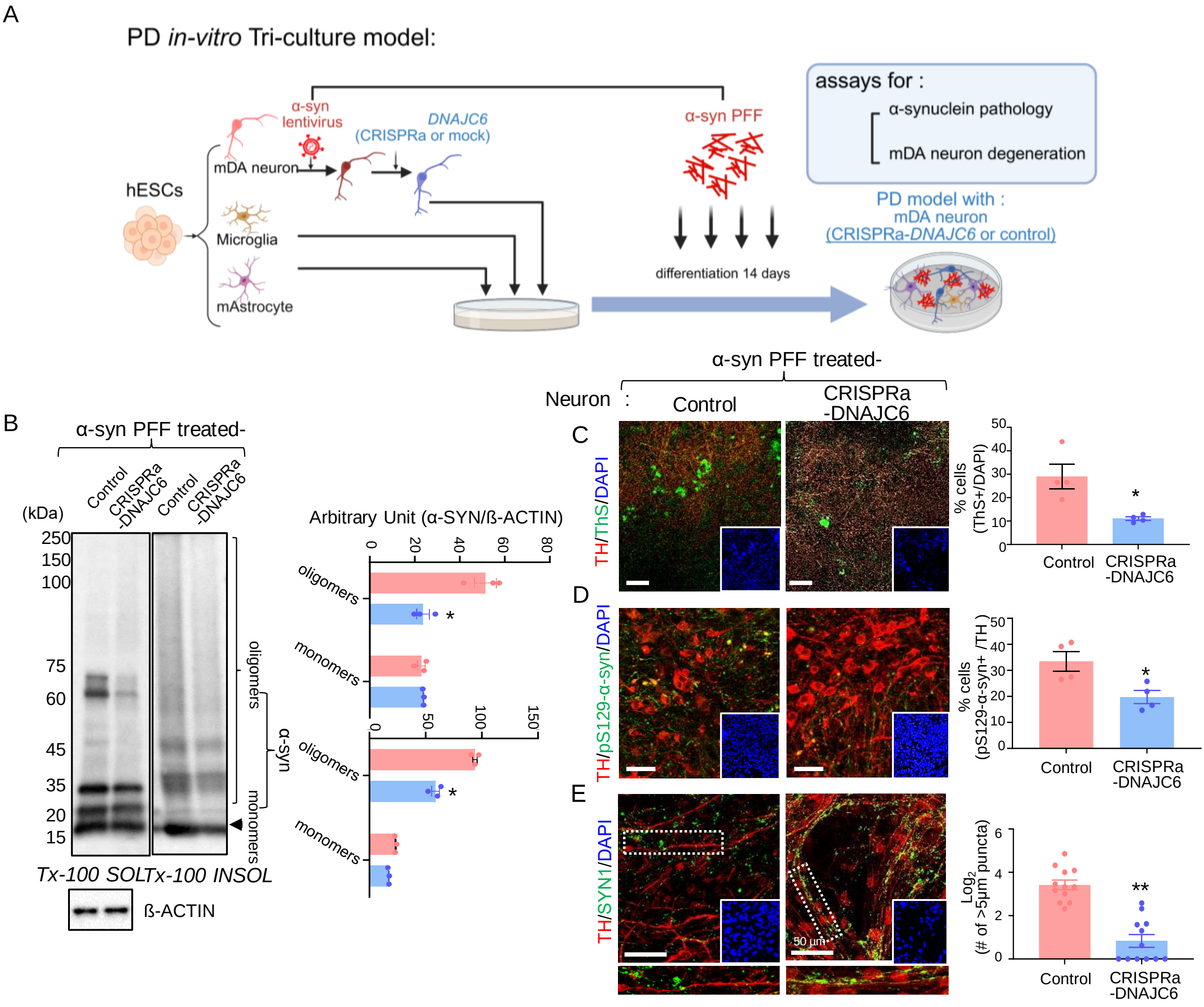
A
PD in-vitro Tri-culture model:
hESCs
mDA neuron
Microglia
mAstrocyte
α-syn lentivirus
DNAJC6 (CRISPRa or mock)
α-syn PFF
differentiation 14 days
assays for :
α-synuclein pathology
mDA neuron degeneration
PD model with : mDA neuron (CRISPRa-DNAJC6 or control)
B
α-syn PFF treated-
Control
CRISPRa -DNAJC6
(kDa)
250
150
100
75
60
45
35
20
15
oligomers
monomers
α-syn
Tx-100 SOL
Tx-100 INSOL
ß-ACTIN
Arbitrary Unit (α-SYN/ß-ACTIN)
0
20
40
60
80
50
100
150
oligomers
monomers
*
α-syn PFF treated-
Neuron :
Control
CRISPRa -DNAJC6
C
TH/ThS/DAPI
% cells (ThS+/DAPI)
D
TH/pS129-α-syn/DAPI
% cells (pS129-α-syn+ /TH)
E
TH/SYN1/DAPI
50 μm
Log2 (# of >5μm puncta)
**

Figure S17.

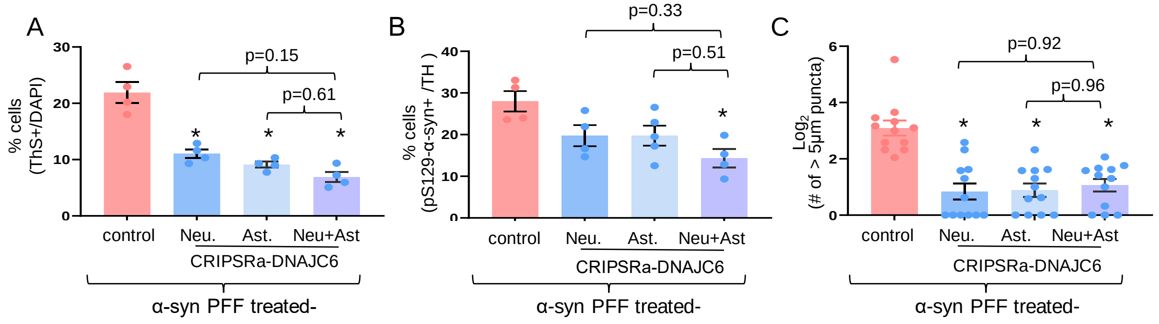

A
% cells (ThS+/DAPI)
30
20
10
0
p=0.15
p=0.61
*
*
*
control
Neu.
Ast.
Neu+Ast
CRIPSRa-DNAJC6
α-syn PFF treated-
B
% cells (pS129-α-syn+ /TH)
40
30
20
10
0
p=0.33
p=0.51
*
control
Neu.
Ast.
Neu+Ast
CRIPSRa-DNAJC6
α-syn PFF treated-
C
Log2 (# of > 5μm puncta)
6
4
2
0
p=0.92
p=0.96
*
*
*
control
Neu.
Ast.
Neu+Ast
CRIPSRa-DNAJC6
α-syn PFF treated-

Figure S18.

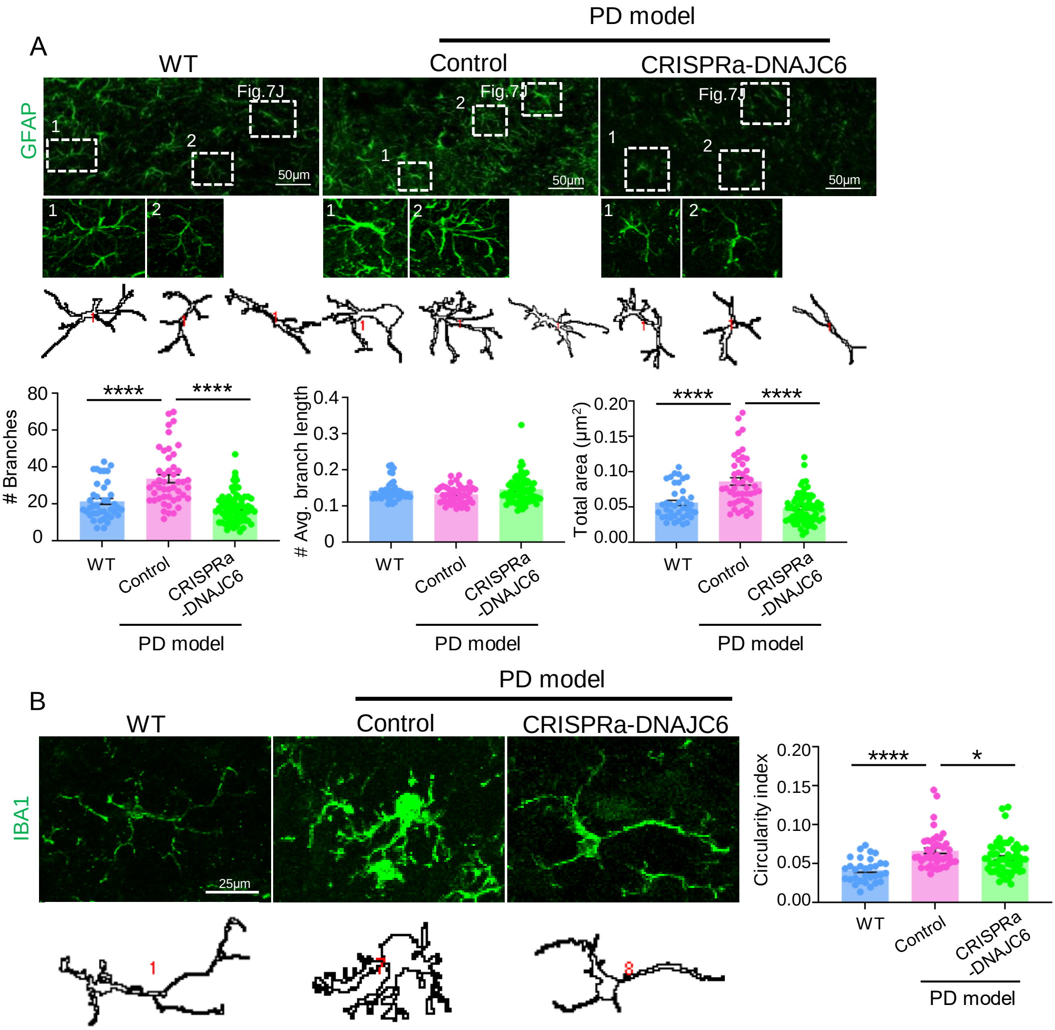
PD model
A
WT
Control
CRISPRa-DNAJC6
GFAP
Fig.7J
50μm
Branches
Avg. branch length
Total area (μm²)
****
WT
Control
CRISPRa -DNAJC6
PD model
B
IBA1
25μm
Circularity index
*

Figure S19.

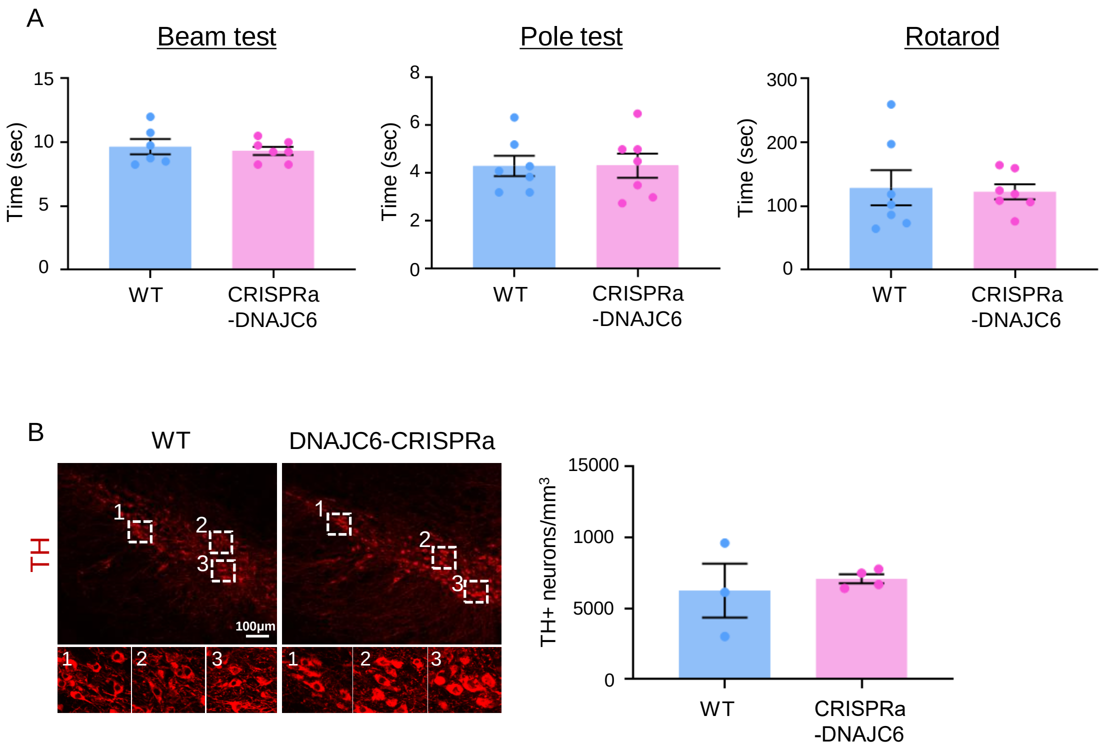

A
Beam test
Time (sec)
15
10
5
0
WT
CRISPRa
-DNAJC6
Pole test
Time (sec)
8
6
4
2
0
WT
CRISPRa
-DNAJC6
Rotarod
Time (sec)
300
200
100
0
WT
CRISPRa
-DNAJC6
B
WT
DNAJC6-CRISPRa
TH
1
2
3
100μm
TH+ neurons/mm³
15000
1000
5000
0
WT
CRISPRa
-DNAJC6

Figure S20.

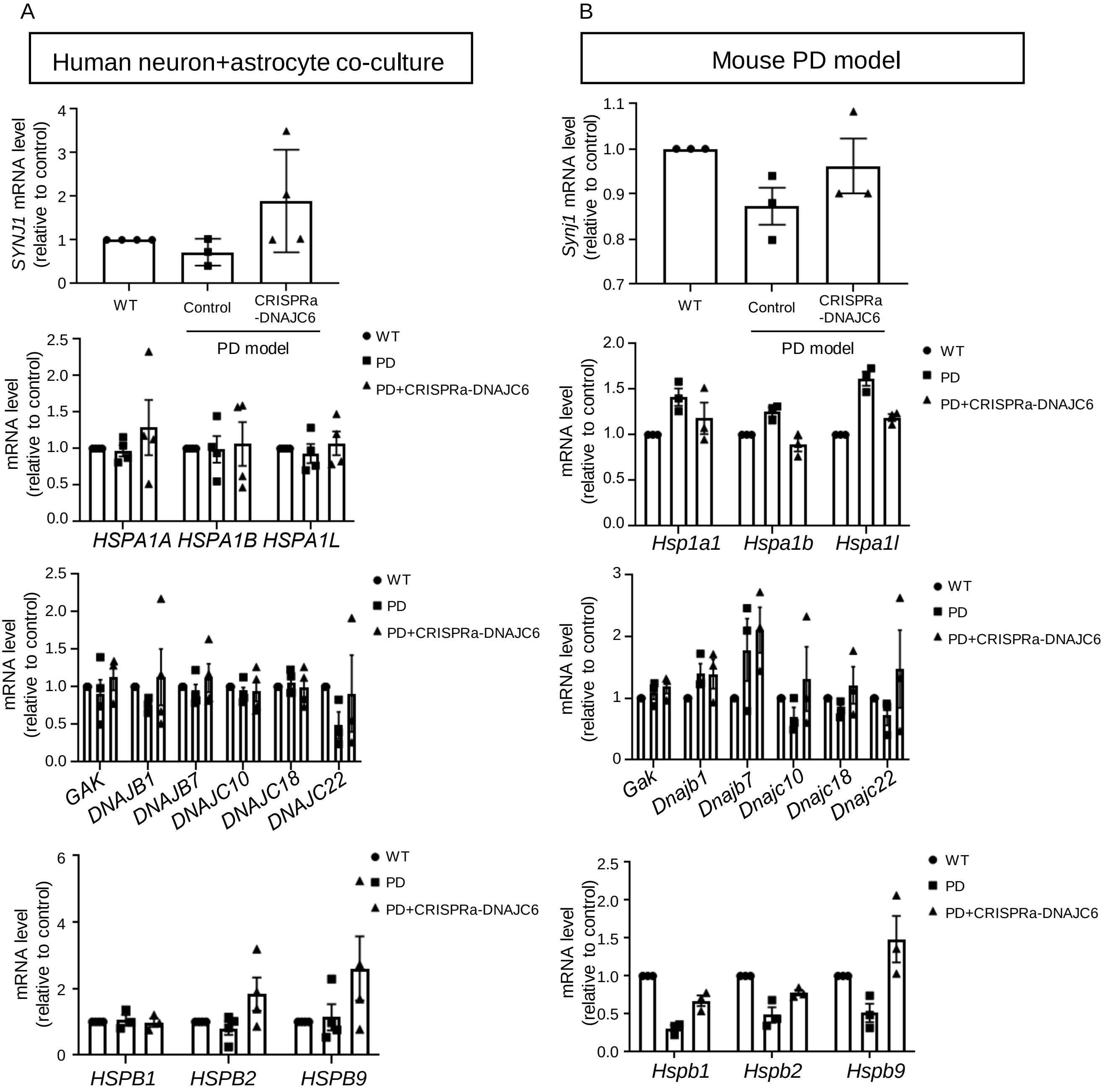

A
B
Human neuron+astrocyte co-culture
Mouse PD model
SYNJ1 mRNA level (relative to control)
Synj1 mRNA level (relative to control)
WT
Control
CRISPRa -DNAJC6
PD model
mRNA level (relative to control)
WT
PD
PD+CRISPRa-DNAJC6
HSPA1A HSPA1B HSPA1L
Hsp1a1 Hspa1b Hspa1l
GAK DNAJB1 DNAJB7 DNAJC10 DNAJC18 DNAJC22
Gak Dnajb1 Dnajb7 Dnajc10 Dnajc18 Dnajc22
HSPB1 HSPB2 HSPB9
Hspb1 Hspb2 Hspb9
