# Supplementary material for: Dysregulation of astrocytic DNAJC6 contributes to sporadic Parkinson’s disease pathogenesis
Source: J Clin Invest. 2026 Apr 9;136(11):e194989. doi: 10.1172/JCI194989 (PMC13221221; doi:10.1172/JCI194989)

Figure 1G

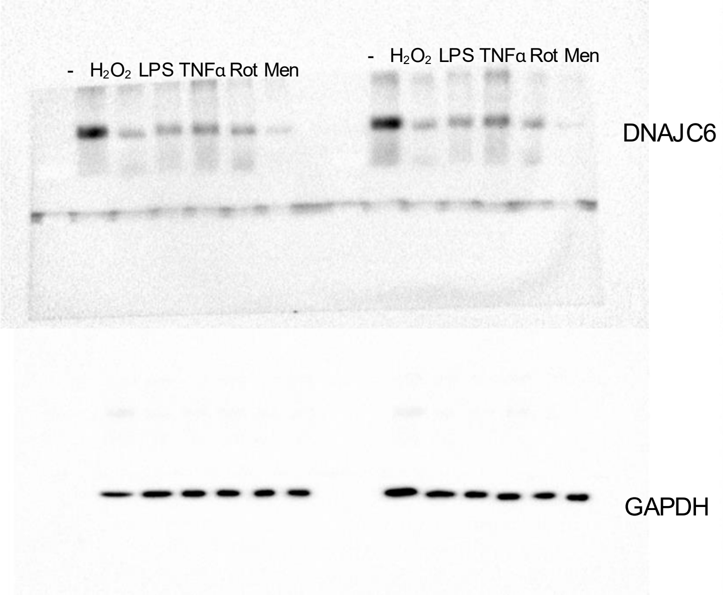

Figure 1H

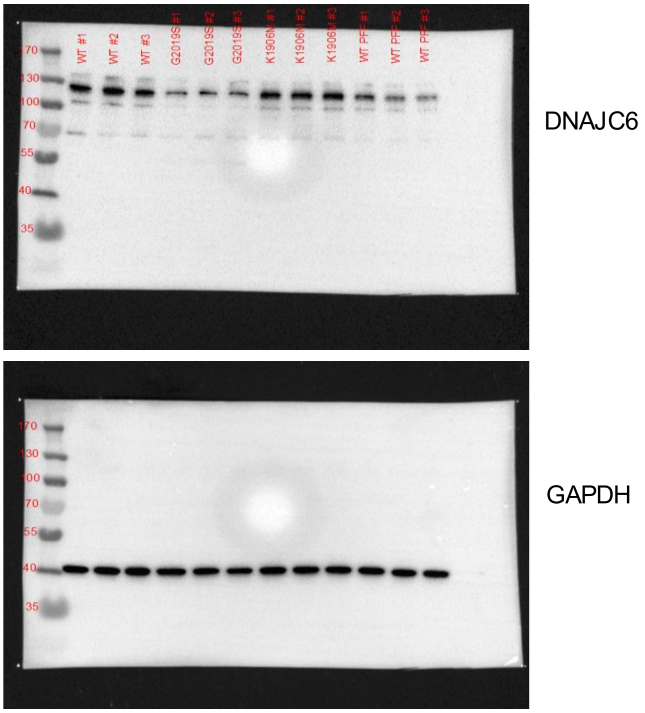

Figure 1I

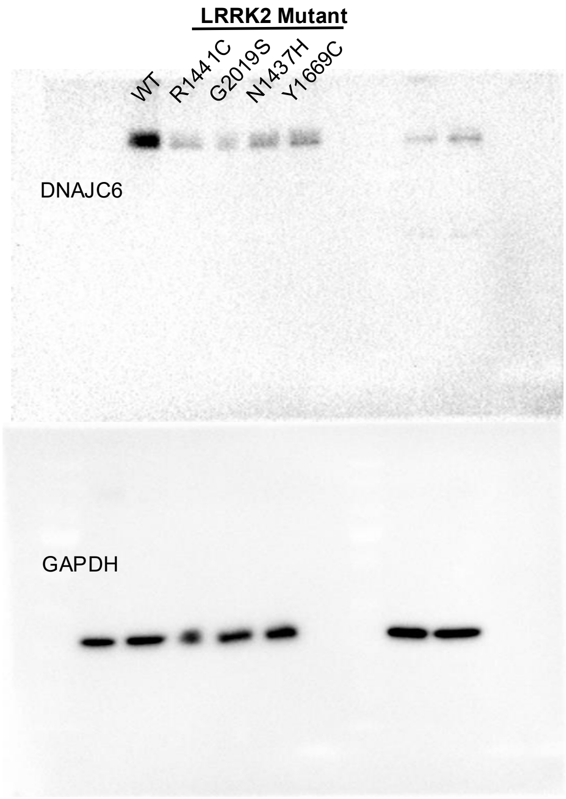

Figure 2I

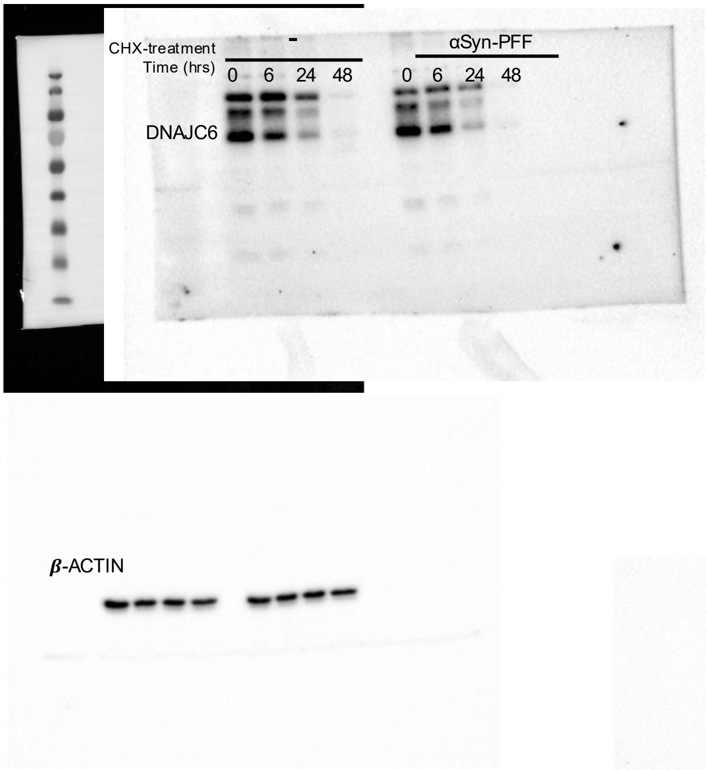

Figure 2J

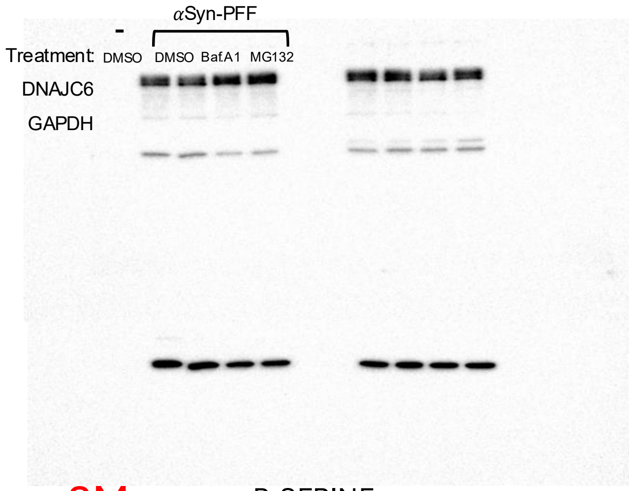

Figure 2M

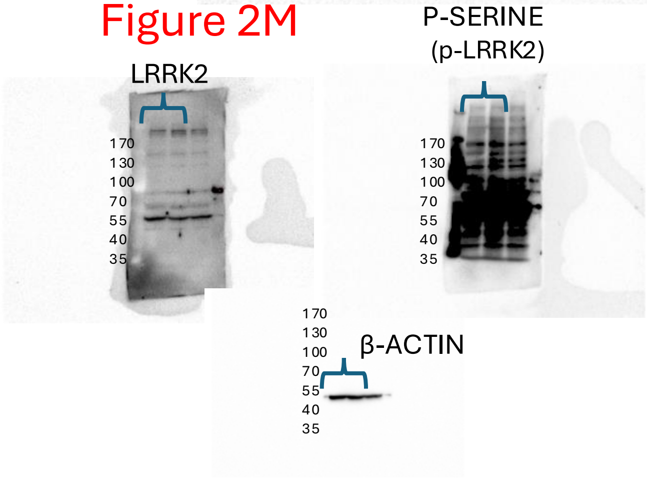

Figure 2K

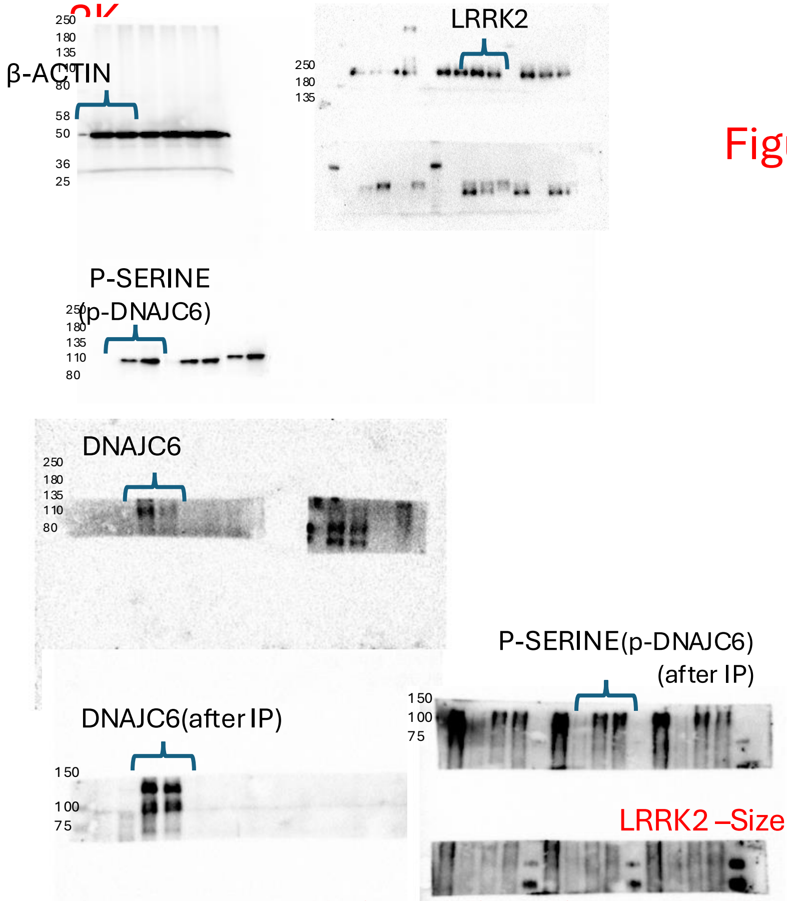

LRRK2 –Size marker

Horizontal flipped image of LRRK2 blot in Figure 3E

Figure 2N

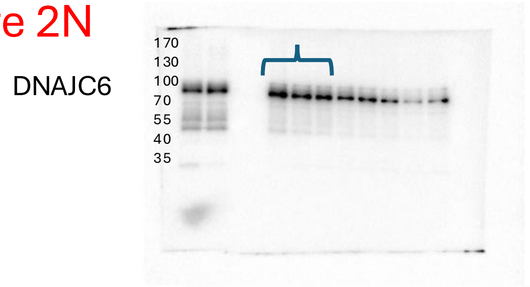

Figure 2O

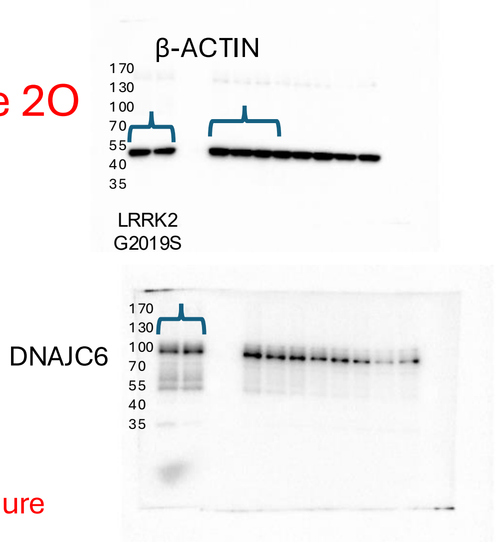

Figure 3A

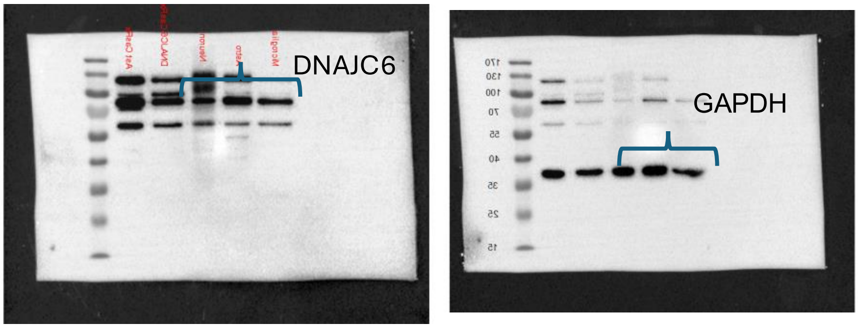

Figure 3C

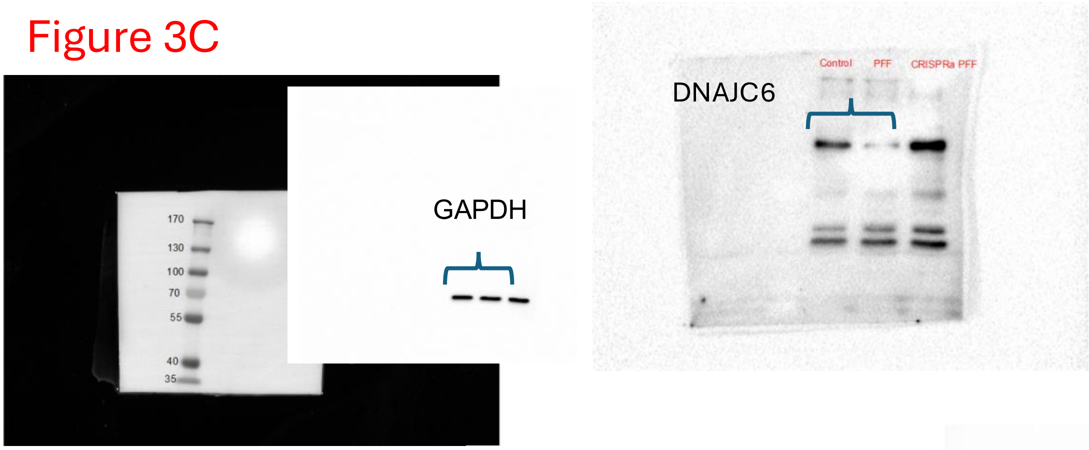

Figure 3D

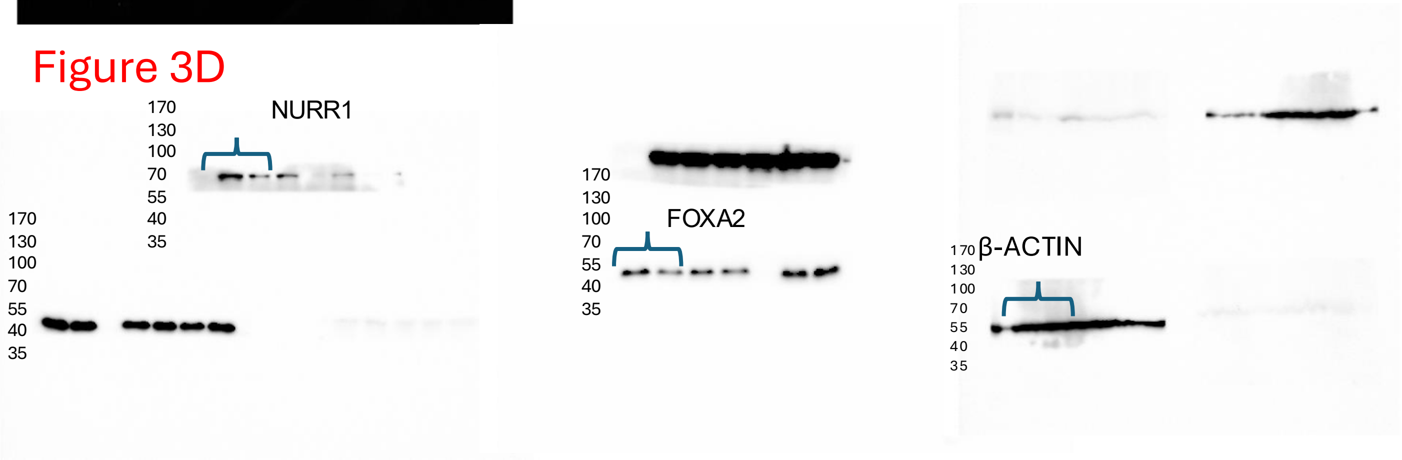

Figure 3E

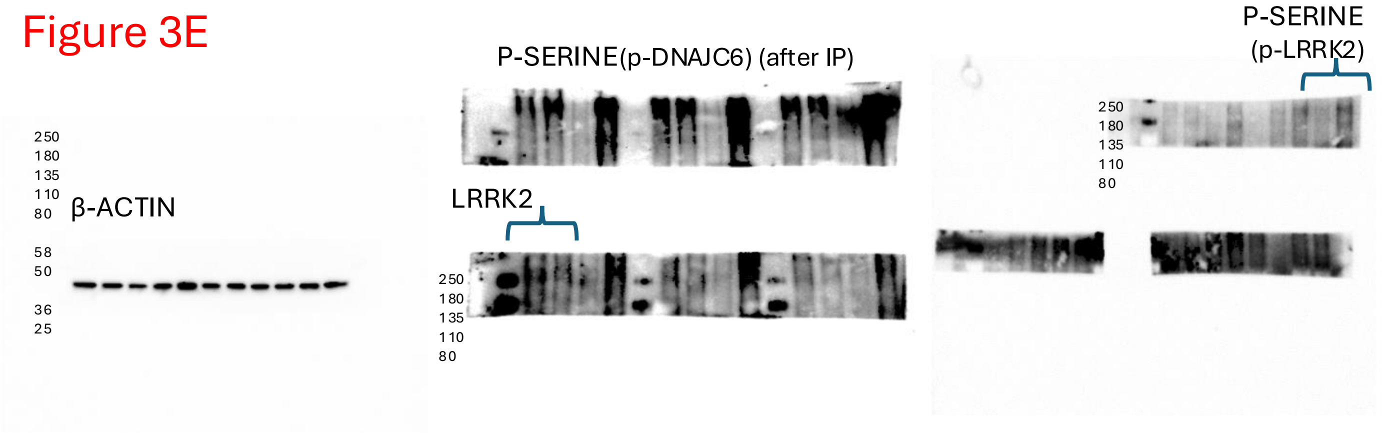

Figure 4C

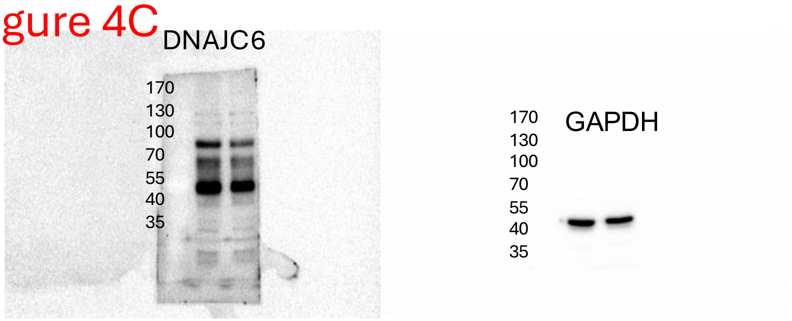

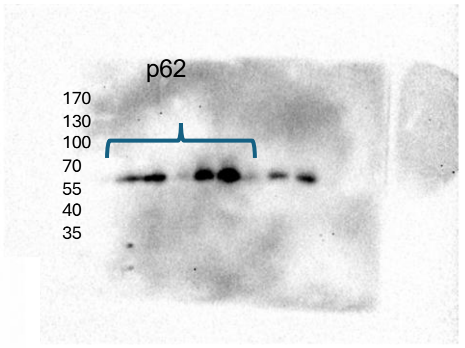

Figure 4H

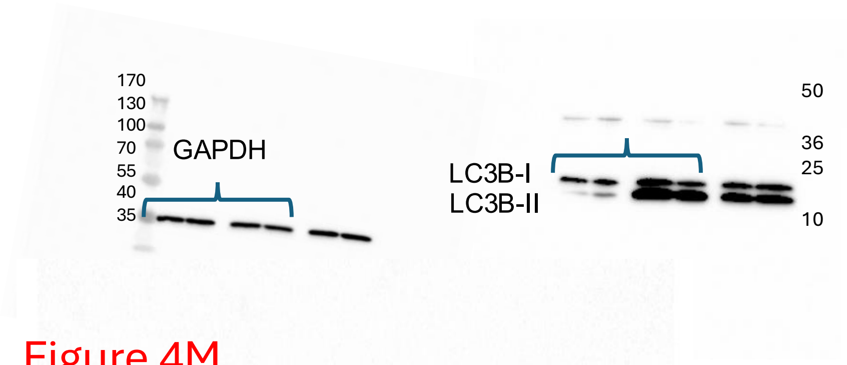

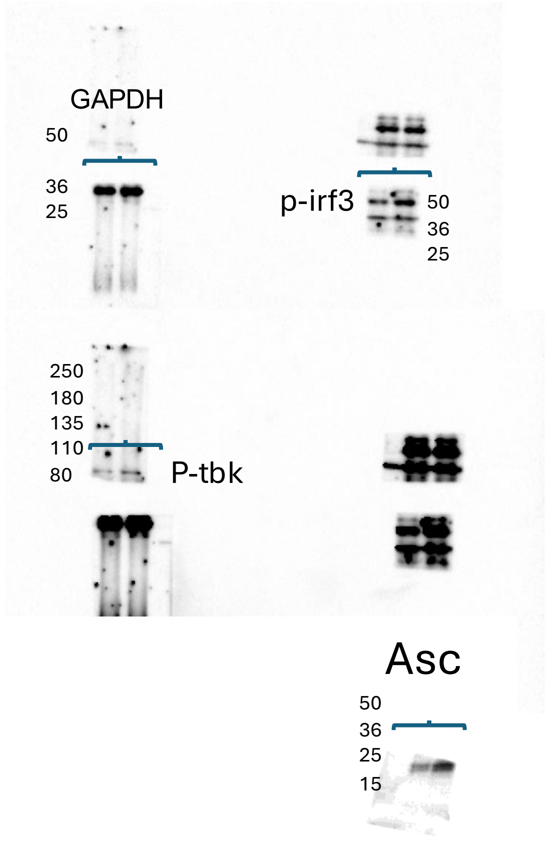

Figure 4M

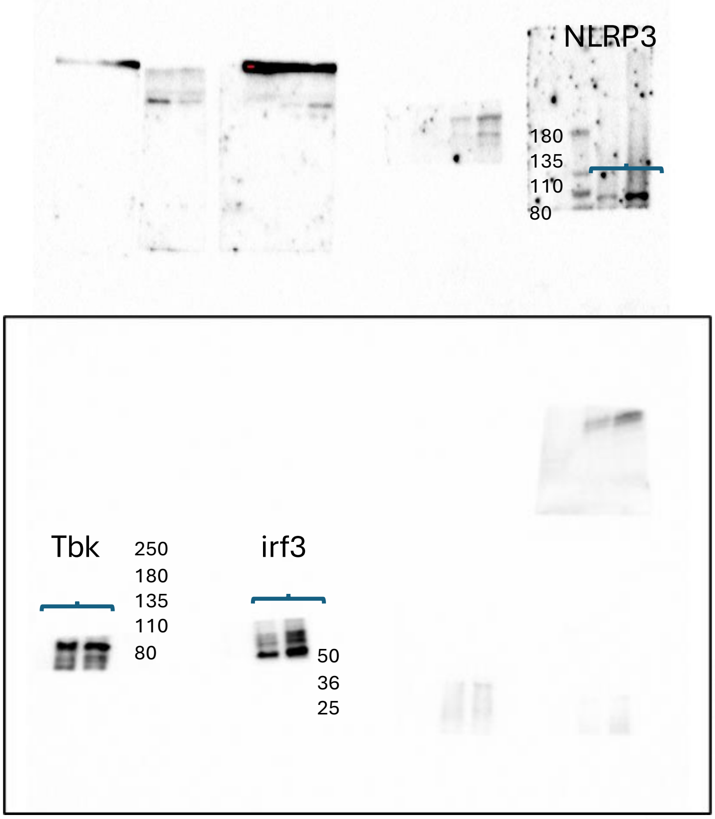

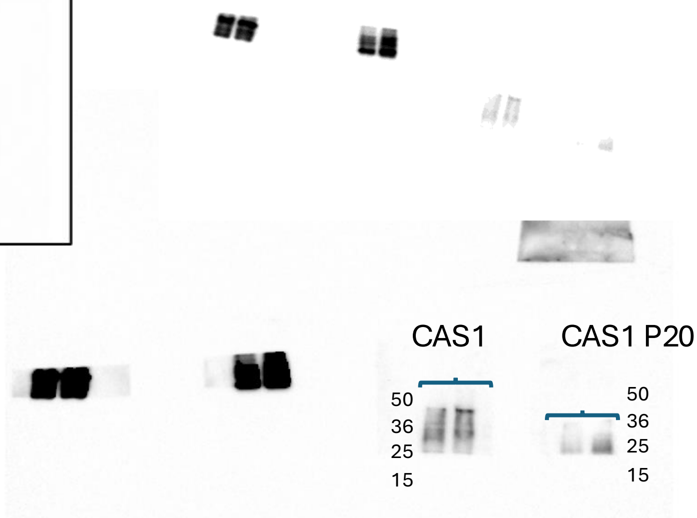

Figure 5B

α-syn

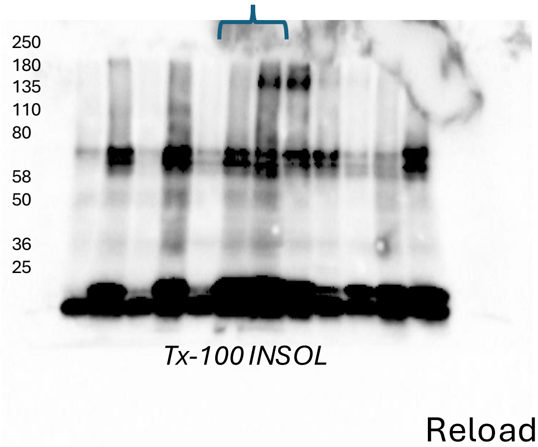

α-syn

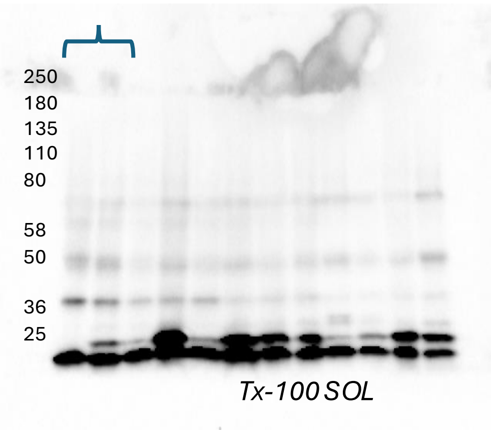

Reload

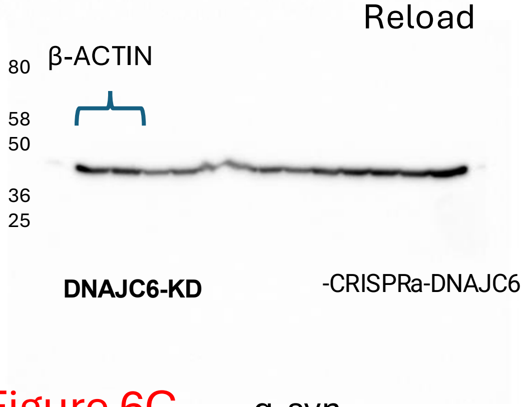

Figure 6C

α-syn

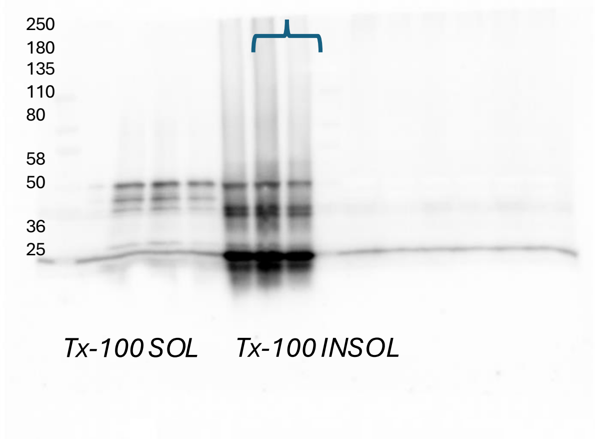

Reload

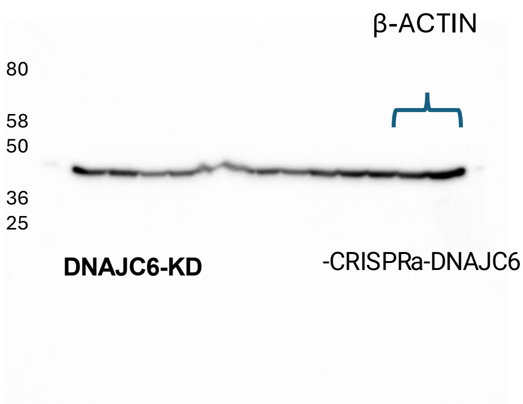

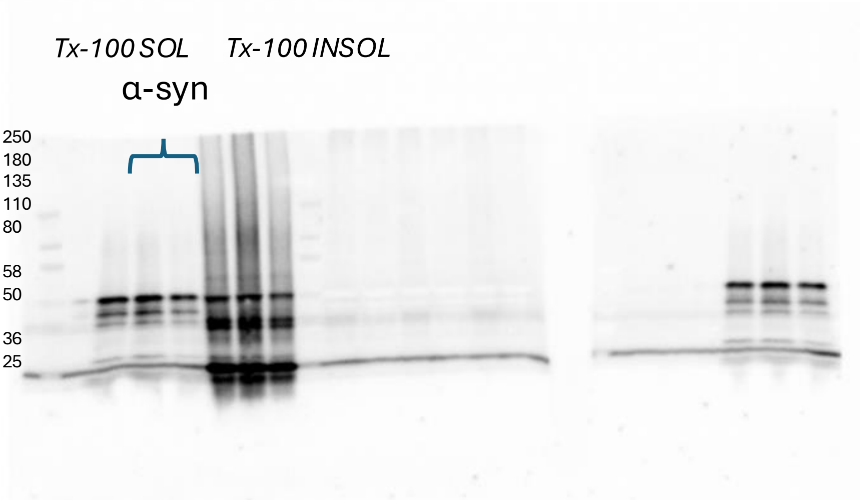

Figure 7

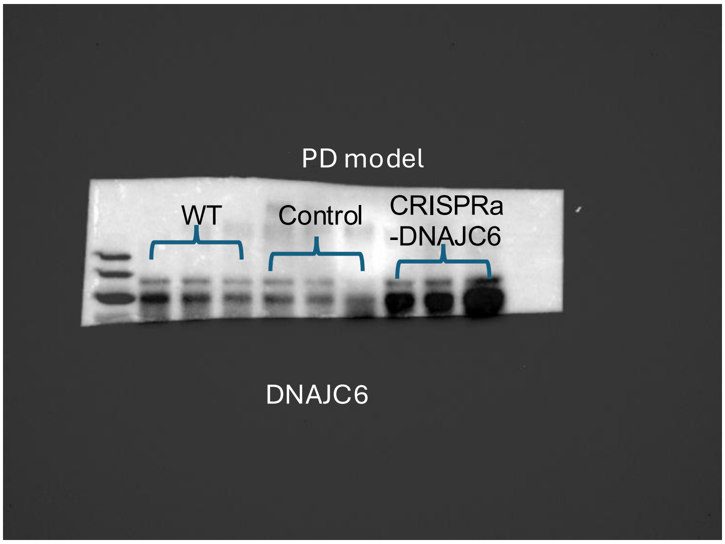
PD model
WT
Control
CRISPRa
-DNAJC6
DNAJC6

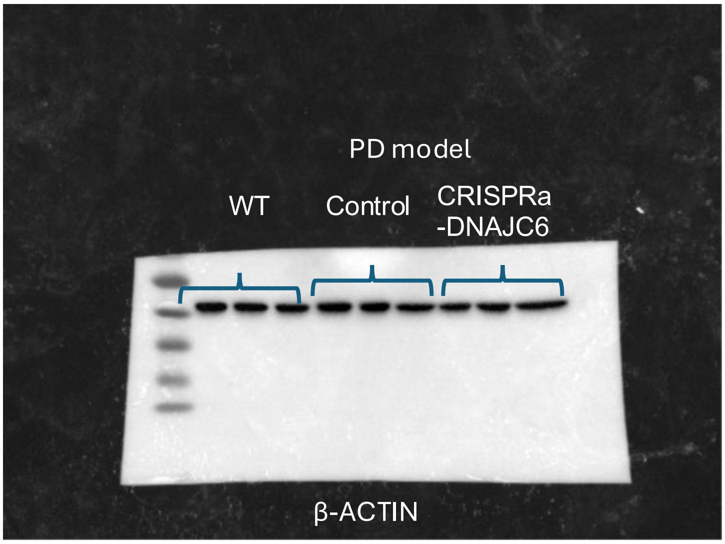
PD model
WT
Control
CRISPRa
-DNAJC6
β-ACTIN

Figure S6.

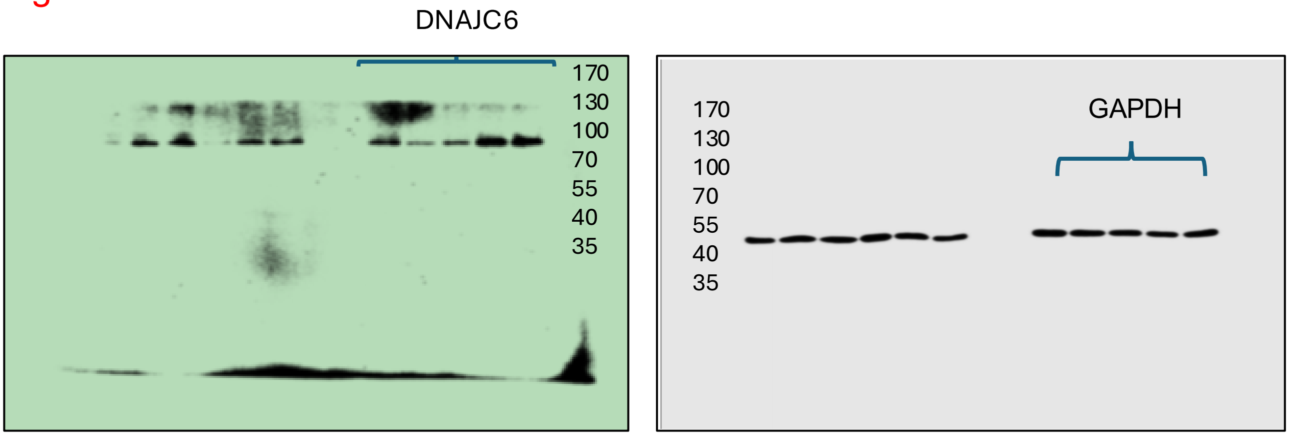

Figure S7.

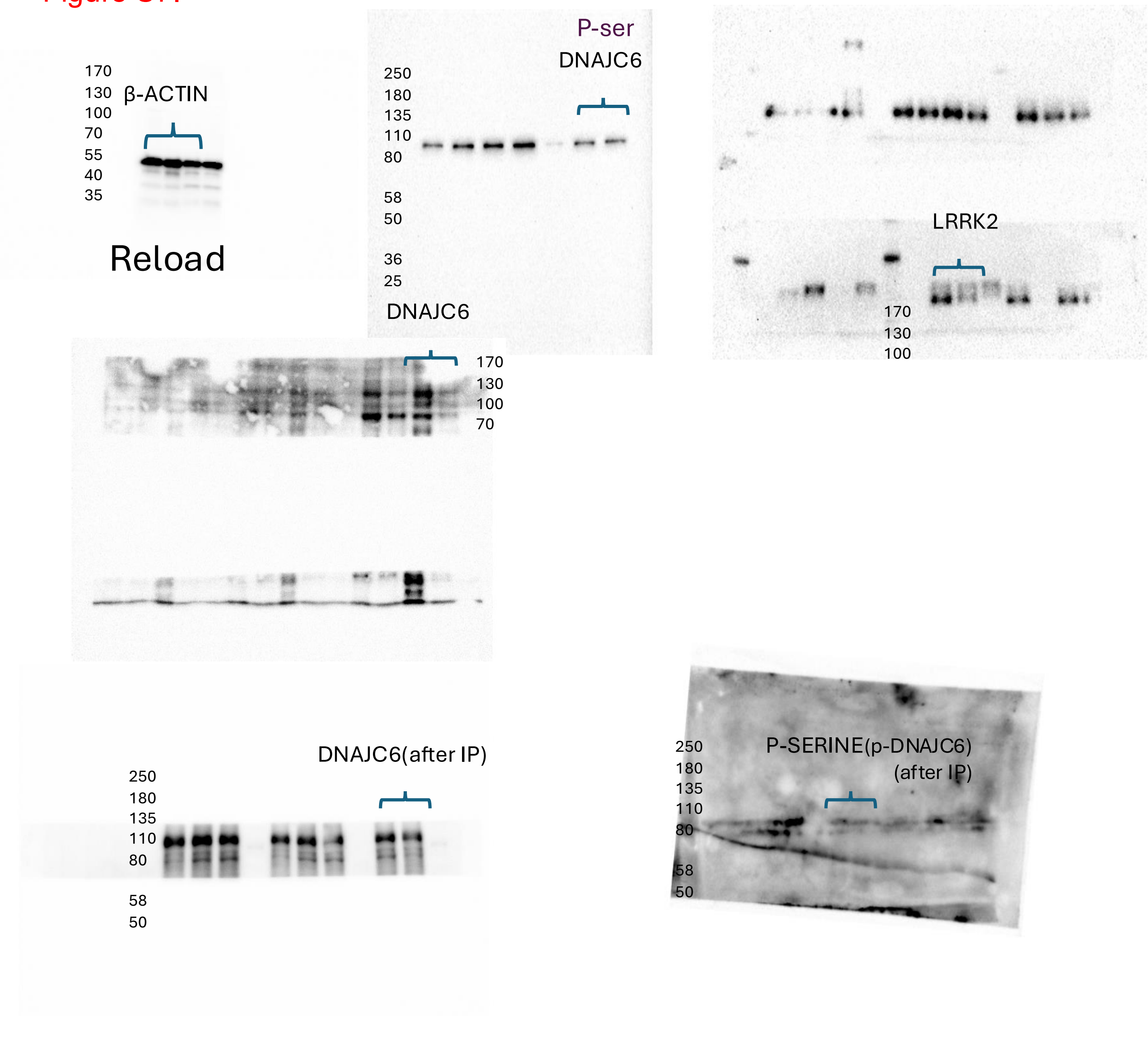

Figure S10.

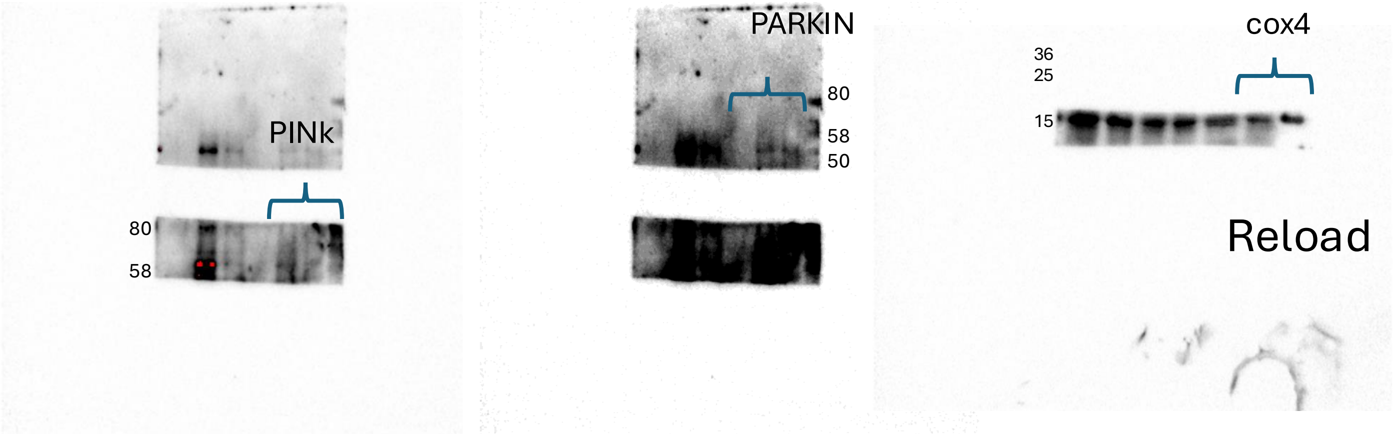

Figure S11.

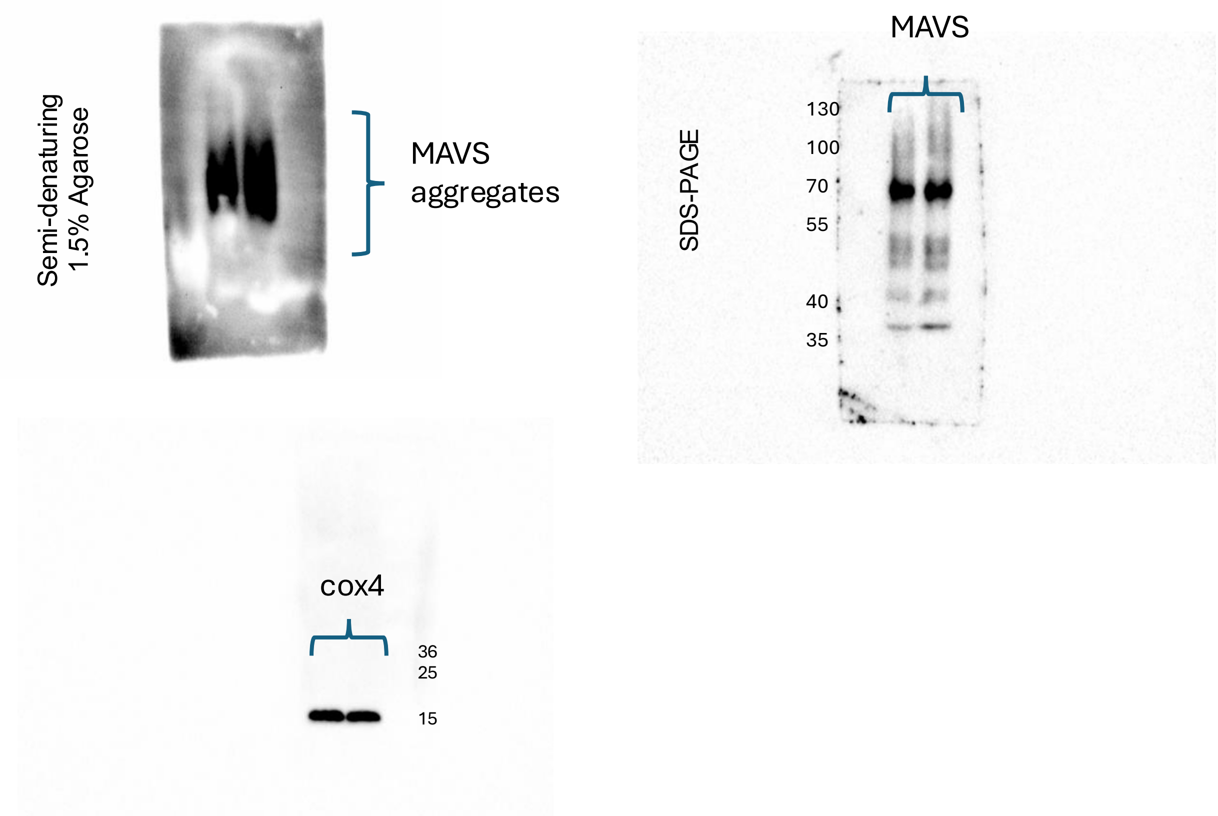

Figure S12B

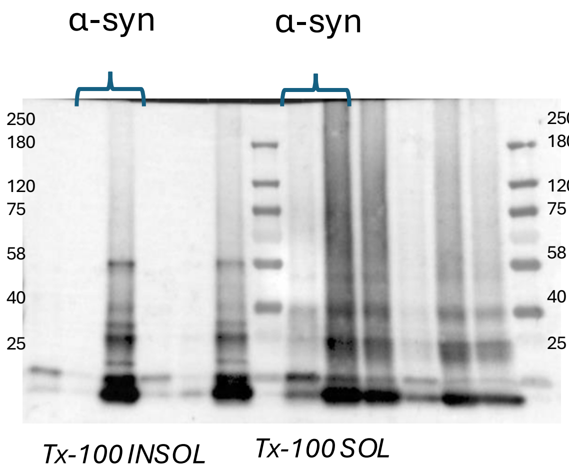

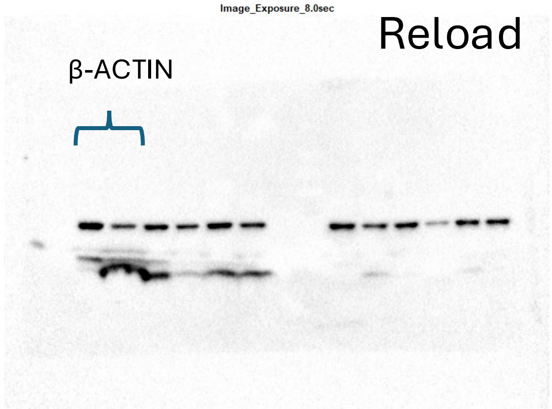

Figure S13A

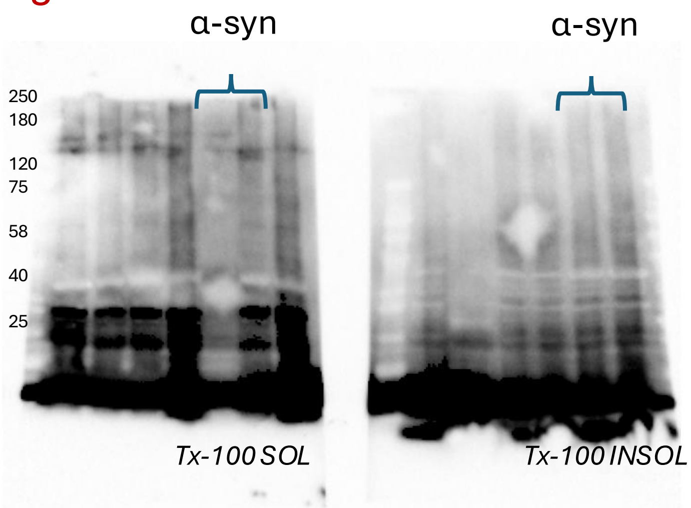

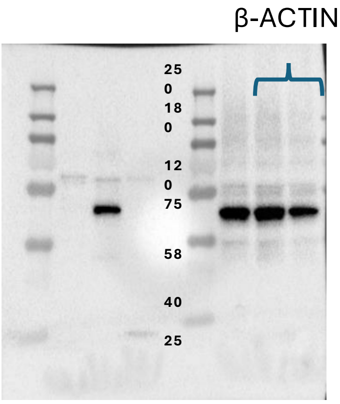

Figure S16B

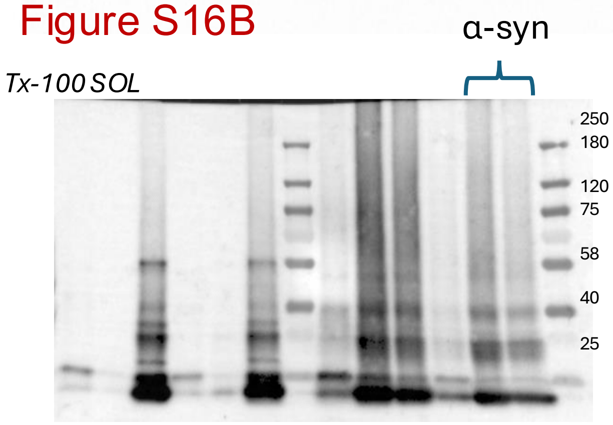

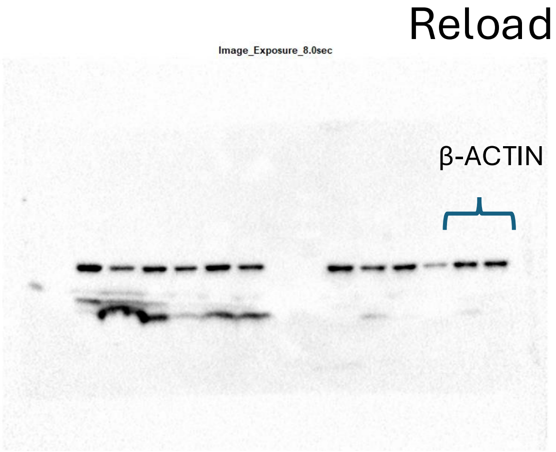

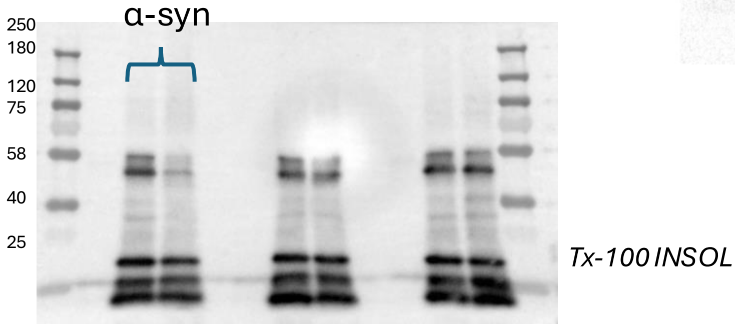

Supplement: Unedited blot and gel images [file jci-136-194989-s009.pdf]
